# Development of novel bioassays to detect soluble and aggregated Huntingtin proteins on three technology platforms

**DOI:** 10.1093/braincomms/fcaa231

**Published:** 2021-01-05

**Authors:** Christian Landles, Rebecca E Milton, Alexandre Jean, Stuart McLarnon, Sean J McAteer, Bridget A Taxy, Georgina F Osborne, Chuangchuang Zhang, Wenzhen Duan, David Howland, Gillian P Bates

**Affiliations:** 1 Department of Neurodegenerative Disease, Huntington’s Disease Centre, UK Dementia Research Institute at UCL, Queen Square Institute of Neurology, University College London, London, UK; 2 Perkin Elmer Inc., Seer Green, UK; 3 Department of Psychiatry and Behavioral Sciences, Johns Hopkins University School of Medicine, Baltimore, MD, USA; 4 Department of Neuroscience, Johns Hopkins University School of Medicine, Baltimore, MD, USA; 5 CHDI Management/CHDI Foundation Inc., New York, NY, USA

**Keywords:** Huntington’s disease, huntingtin bioassay, polyglutamine, huntingtin aggregation, zQ175 knock-in mouse model

## Abstract

Huntington’s disease is caused by a CAG / polyglutamine repeat expansion. Mutated CAG repeats undergo somatic instability, resulting in tracts of several hundred CAGs in the brain; and genetic modifiers of Huntington’s disease have indicated that somatic instability is a major driver of age of onset and disease progression. As the CAG repeat expands, the likelihood that exon 1 does not splice to exon 2 increases, resulting in two transcripts that encode full-length huntingtin protein, as well as the highly pathogenic and aggregation-prone exon 1 huntingtin protein. Strategies that target the huntingtin gene or transcripts are a major focus of therapeutic development. It is essential that the levels of all isoforms of huntingtin protein can be tracked, to better understand the molecular pathogenesis, and to assess the impact of huntingtin protein-lowering approaches in preclinical studies and clinical trials. Huntingtin protein bioassays for soluble and aggregated forms of huntingtin protein are in widespread use on the homogeneous time-resolved fluorescence and Meso Scale Discovery platforms, but these do not distinguish between exon 1 huntingtin protein and full-length huntingtin protein. In addition, they are frequently used to quantify huntingtin protein levels in the context of highly expanded polyglutamine tracts, for which appropriate protein standards do not currently exist. Here, we set out to develop novel huntingtin protein bioassays to ensure that all soluble huntingtin protein isoforms could be distinguished. We utilized the zQ175 Huntington’s disease mouse model that has ∼190 CAGs, a CAG repeat size for which protein standards are not available. Initially, 30 combinations of six antibodies were tested on three technology platforms: homogeneous time-resolved fluorescence, amplified luminescent proximity homogeneous assay and Meso Scale Discovery, and a triage strategy was employed to select the best assays. We found that, without a polyglutamine-length-matched standard, the vast majority of soluble mutant huntingtin protein assays cannot be used for quantitative purposes, as the highly expanded polyglutamine tract decreased assay performance. The combination of our novel assays, with those already in existence, provides a tool-kit to track: total soluble mutant huntingtin protein, soluble exon 1 huntingtin protein, soluble mutant huntingtin protein (excluding the exon 1 huntingtin protein) and total soluble full-length huntingtin protein (mutant and wild type). Several novel aggregation assays were also developed that track with disease progression. These selected assays can be used to compare the levels of huntingtin protein isoforms in a wide variety of mouse models of Huntington’s disease and to determine how these change in response to genetic or therapeutic manipulations.

## Teaser

Landles *et al.* report that they have developed novel bioassays to provide a tool kit for the detection of soluble and aggregated isoforms of the HTT protein in tissue lysates from mouse models of Huntington’s disease on three bioassay platforms.

## Graphical Abstract

**Graphical Abstract:**
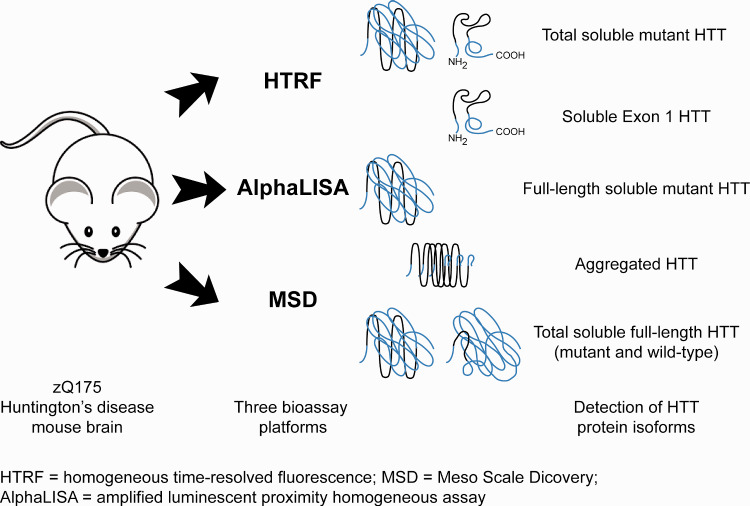

## Introduction

Huntington’s disease is an inherited neurodegenerative disorder that manifests with cognitive, motor and psychiatric abnormalities (Bates *et al.*, 2015). It is caused by a cytosine adenine guanine (CAG) repeat expansion in exon 1 of the huntingtin gene (*HTT*) that is translated to an abnormally long polyglutamine (polyQ) tract in the huntingtin protein (HTT) (HDCRG, 1993). Expansions of 40 or more CAGs are fully penetrant mutations, individuals with repeats of 35 CAGs or less remain unaffected, whereas as those with between 36 and 39 CAGs have an increasing risk of developing Huntington’s disease within a normal lifespan (Rubinsztein *et al.*, 1996). CAG repeat expansions greater than ∼65 CAGs result in disease onset in childhood or adolescence (Telenius *et al.*, 1993). Mutant HTT can self-assemble in a concentration, time, polyQ length and N-terminal HTT-fragment length-dependent manner (Scherzinger *et al.*, 1999). It forms inclusion bodies in the brains of Huntington’s disease patients (DiFiglia *et al.*, 1997; Gutekunst *et al.*, 1999) and mouse models of Huntington’s disease (Davies *et al.*, 1997; Li *et al.*, 1999), which are complex structures that sequester many other proteins (Hosp *et al.*, 2017). The neuropathology of Huntington’s disease is characterized by neuronal cell loss in the cortex, striatum and other brain regions (Vonsattel and DiFiglia, 1998; Nana *et al.*, 2014).

It is essential that one can measure levels of soluble and aggregated HTT isoforms, to track how these change in relation to disease onset and progression, and how they respond to potential therapeutic interventions. Considerable progress in establishing antibody-based assays for this purpose has been made over the past 10 years. These have included homogeneous time-resolved fluorescence assays (HTRF, also termed TR-FRET) that detect soluble mutant HTT (Weiss *et al.*, 2009), both soluble mutant and wild-type HTT (Weiss *et al.*, 2011) and aggregated HTT (Baldo *et al.*, 2012) in cultured cells and tissues from mouse models of Huntington’s disease. Assays that measure soluble (Macdonald *et al.*, 2014) and aggregated (Reindl *et al.*, 2019) HTT species have also been established on the Meso Scale Discovery (MSD) platform, and soluble mutant HTT on the amplified luminescent proximity homogeneous assay (AlphaLISA) platform (Baldo *et al.*, 2018). More sensitive single-molecule counting assays, that can measure soluble mutant HTT in CSF, have been developed (Wild *et al.*, 2015) and used to provide a pharmacodynamic read out in clinical trials that evaluate the HTT lowering efficiency of an antisense oligonucleotide targeting the *HTT* transcript (Tabrizi *et al.*, 2019b).

Assays developed on the HTRF, AlphaLISA and MSD platforms utilize pairs of antibodies that bind to the same protein analyte. For HTRF, the donor antibody is bound to a fluorophore, which when excited transfers energy to an acceptor fluorophore-bound antibody which in turn emits long-lived fluorescence (Degorce *et al.*, 2009). For AlphaLISA, a biotinylated antibody is bound to a streptavidin-coated alpha donor bead, which transfers a reactive form of oxygen to an antibody-conjugated AlphaLISA acceptor bead (Eglen *et al.*, 2008). In the case of MSD, a capture antibody is bound to an electrode and the sulpho-tagged detection antibody emits light, upon electrochemical stimulation initiated at the electrode surface. In all cases, these are complex assays that depend on the avidity of two antibodies and for which the performance may differ, depending on the matrix in which the analyte is being tested (tissue, blood, cell culture medium, etc.). These assays are only quantitative so long as the concentration of the analyte can be read from a standard curve, which in the case of Huntington’s disease, is not straight forward. Currently, the HTRF and MSD assays are often used to measure soluble and aggregated HTT species with polyQ repeat expansions of >100 glutamines, but with glutamine repeats in this range, the HTT fragments generally used as standards aggregate readily and do not remain soluble (Scherzinger *et al.*, 1997).

In this study, we have applied HTRF, AlphaLISA and MSD assays to detect soluble and aggregated HTT isoforms in brain tissues from zQ175 mice, the most commonly used knock-in mouse model of Huntington’s disease (Heikkinen *et al.*, 2012; Menalled *et al.*, 2012). In this model, exon 1 of mouse *Htt* has been replaced with a mutated version of human exon 1 with a repeat expansion of ∼190 CAGs. In zQ175 mice, exon 1 does not always splice to exon 2, resulting in a small polyadenylated *Httexon1* transcript that encodes the highly pathogenic and aggregation-prone exon 1 HTT protein (Sathasivam *et al.*, 2013; Papadopoulou *et al.*, 2019). To complement the HTRF and MSD assays already available, we set out to develop novel assays specific to ‘soluble exon 1 HTT’, as well as assays that detect soluble mutant HTT, excluding the exon 1 HTT protein. We began by testing six anti-HTT antibodies (2B7, MW1, 4C9, MW8, MAB5490 and MAB2166) in 30 combinations on each of the HTRF, AlphaLISA and MSD platforms, and then used a sequential triage approach to select the most useful antibody pairings. Incorporated within this, the effect of the zQ175 polyQ tract on assay performance was assessed, and we found that for many, including assays commonly in use, this highly expanded polyQ repeat decreased signal intensity.

Here, we describe assays that detect (i) total soluble mutant HTT, (ii) soluble exon 1 HTT, (iii) soluble mutant HTT (excluding exon 1 HTT), (iv) aggregated HTT and (v) total soluble full-length HTT (mutant and wild type). We found that exon 1 HTT protein levels decreased with disease progression in zQ175 mice, but that the level of soluble mutant HTT (excluding exon 1 HTT) remained unchanged. Furthermore, we have identified novel HTT aggregation assays, and provide evidence for the recruitment of HTT fragments longer than exon 1 HTT into aggregates. We failed to identify any soluble mutant HTT assays that could be used for quantitation in the absence of a protein standard that is polyQ-length matched to the HTT analyte. The HTRF, AlphaLISA or MSD assays identified in this study can now be used to determine the relative levels of HTT isoforms in zQ175 mice. They can be applied to other mouse models of Huntington’s disease although we would recommend first optimizing the concentration of antibody and lysate dilution, as these might change, depending on polyQ repeat length and HTT protein concentration.

## Materials and methods

### Mouse breeding and maintenance

All procedures were performed in accordance with the Animals (Scientific Procedures) Act 1986 and were approved by the University College London Ethical Review Process Committee. This study was directed towards tracking changes in HTT isoform levels with disease progression in zQ175 mice; but tissues from *Hdh*Q20, YAC128, N171-82Q and R6/2 mice were also used in the development and optimization of the assays. zQ175 knock-in mice were generated by replacing exon 1 of mouse *Htt* with exon 1 from human *HTT*, carrying a highly expanded CAG repeat (Heikkinen *et al.*, 2012; Menalled *et al.*, 2012) from which the neo-selectable marker has been removed (delta neo) (Franich *et al.*, 2019). These were either bred in-house by backcrossing males to C57BL/6J females (Charles River), or obtained from the CHDI Foundation colony at the Jackson Laboratory (Bar Harbor, Maine) on a C57BL/6J background. YAC128 mice (Slow *et al.*, 2003) were bred in-house by backcrossing males to C57BL/6J females (Charles River). *Hdh*Q20 knock-in mice, in which mouse exon 1 *Htt* has been replaced with human exon 1 *HTT* with 18 CAGs (Wheeler *et al.*, 1999) were obtained from the CHDI Foundation colony at the Jackson Laboratory (Bar Harbor, Maine) on a C57BL/6J background. R6/2 mice were bred in-house by backcrossing R6/2 males to C57BL/6JOlaHsd × CBA/CaOlaHsd F1 females (B6CBAF1/OlaHsd, Envigo, The Netherlands). Transgenic N171-82Q mice (Schilling et al., 1999) on a B6C3F1/J hybrid background were bred by backcrossing N171-82Q males to B6C3F1/J females (Jackson Laboratory, Bar Harbor, Maine) and were obtained from Wenzhen Duan’s colony at Johns University Hopkins, Baltimore, USA. All procedures were approved by the Institutional Animal Care and Use Committee of the Johns Hopkins University.

Within each colony, genetically modified and wild-type mice were group housed with up to five mice per cage, depending on gender, but genotypes were mixed. Mice were housed in individually ventilated cages with Aspen Chips 4 Premium bedding (Datesand) with environmental enrichment which included chew sticks and play tunnel (Datesand). They had unrestricted access to food (Teklad global 18% protein diet, Envigo) and water. The temperature was regulated at 21°C ± 1°C and animals were kept on a 12 h light/dark cycle. The animal facility was barrier-maintained and quarterly non-sacrificial FELASA screens found no evidence of pathogens. Mice were sacrificed by a schedule 1 procedure, brains were rapidly dissected, tissues were snap frozen in liquid nitrogen and stored at −80°C.

### Genotyping and CAG repeat sizing

Genotyping and CAG repeat sizing was performed by PCR of ear-biopsy DNA and all primers were from Invitrogen.

For zQ175 mice, a 20 μl genotyping reaction contained 10–15 ng DNA, GoTaq Flexi buffer, 2.5 mM MgCl_2_, 0.2 mM dNTPs, 1.0 μM 19Fhum [5′-AGGAGCCGCTGCACCGA-3′], 1.0 μM 431R2 [5′-CTCTTCACAACAGTCATGTGCG-3′] and 1.0 U GoTaq2 polymerase (Promega). Cycling conditions were 30 s at 98°C, 34× (15 s at 98°C, 15 s at 64°C, 30 s at 72°C), 5 min at 72°C. The amplified products were 324 bp for wild type and 240 bp for zQ175. For CAG repeat sizing, a 25 μl reaction contained 25–50 ng DNA, 0.2 μM FAM-labelled forward primer [5′-CCTTCGAGTCCCTCAAGTCCTT-3′], 0.2 μM reverse primer [5′-CGGCTGAGGCAGCAGCGGCTGT-3′], AmpliTaq Gold 360 Master Mix (Thermo Fisher Scientific) and GC enhancer (Thermo Fisher Scientific). Cycling conditions were 10 min at 95°C, 34× (30 s at 95°C, 30 s at 63°C and 90 s at 72°C), 7 min at 72°C.

For *Hdh*Q20 mice, genotyping was performed using wild-type and knock-in allele-specific PCR reactions. For the wild-type allele, a 20 μl reaction contained 100 ng DNA, GoTaq Flexi buffer, 1.25 mM MgCl_2_, 0.2 mM dNTPs, 10% DMSO, 0.5 μM *Hdh*Up [5′-CCTGGAAAAGCTGATGAAGG-3′], 0.5 μM *Hdh*Down [5′-TGGACAGGGAACAGTGTTGGC-3′] and 1.0 U GoTaq2 (Promega). Cycling conditions were 1.5 min at 94°, 34× (30 s at 94°C, 30 s at 56°C, 1.5 min at 72°C), 10 min at 72°C. The amplified product was 278 bp. For the knock-in allele, a 10 μl reaction contained 50 ng DNA, 2.0 μM GAC1 [5′-ATGAAGGCCTTCGAGTCCCTCAAGTCCTTC-3′], 2.0 μM HU3 [5′-TGGACAGGGAACAGTGTTGGC-3′] and Dream Taq2 Hotstart master mix (Thermo Fisher Scientific). Cycling conditions were 1.5 min at 94°, 34× (30 s at 94°C, 30 s at 65°C, 1.5 min at 72°C), 10 min at 72°C. The amplified product was 140 bp. For CAG repeat sizing, the same knock-in PCR was set up using GAC1-FAM [5′-ATGAAGGCCTTCGAGTCCCTCAAGTCCTTC-3′].

For YAC128 and R6/2 mice, a 10 μl reaction contained 50–100 ng DNA, 1.0 μM forward primer [5′-CGCAGGCTAGGGCTGTCAATCATGCT-3′], 1.0 μM reverse primer [5′-TCATCAGCTTTTCCAGGGTCGCCAT-3′], 1.0 μM forward internal control primer [5′-AGCCCTACACTAGTGTGTGTTACACA-3′], 1.0 μM reverse internal control primer [5′-CTTGTTGAGAACAAACTCCTGCAGCT-3′] and Dream Taq Hot Start Green PCR Master Mix (Thermo Fisher Scientific). Cycling conditions were 3 min at 95°C, 34× (30 s at 95°C, 30 s at 60°C, 1 min at 72°C), 15 min at 72°C. R6/2 mice were CAG repeat sized by the same protocol as was used as for zQ175 mice.

Transgenic N171-82Q mice were identified by PCR of genomic DNA extracted from mouse tail as described previously (Schilling et al., 1999). A three-way PCR was used for genotyping. Two primers were complementary to the prion protein genomic DNA sequence: PrP-sense [5′-CCTCTTGTGACTATGTGGACTGATGTCGG-3′], PrP-anti-sense [5′-GTGGATACCCCCTCCCCCAGCCTAGACC-3′]. The amplified product of this reaction is 700 bp in length. The anti-sense primer is also complementary to the 3′-untranslated portion of the PrP vector and, in combination with a third sense primer to the HD sequence [5′-GAACTTTCAGCTACCAAGAAAGACCGTGT-3′], generated a transgene-specific product that is 250 bp in length. The CAG repeat size in this mouse line is stable.

Samples for CAG repeat sizing were run on an ABI3730XL Genetic Analyser with MapMarker ROX 1000 (Bioventures) internal size standards and analysed using GeneMapper v5 software (Thermo Fisher Scientific). The mean CAG repeat size for all zQ175 mice was 190.61 ± 4.4 (S.D.), the two R6/2 mice had 206 and 207 CAGs, *Hdh*Q20 mice have 18 CAGs and YAC128 have an interrupted CAG repeat of 123 CAGs with (CAA)_3_CAGCAA at positions 24–28 and 109–113 (Pouladi *et al.*, 2012).

### Antibodies

**Figure 1:**
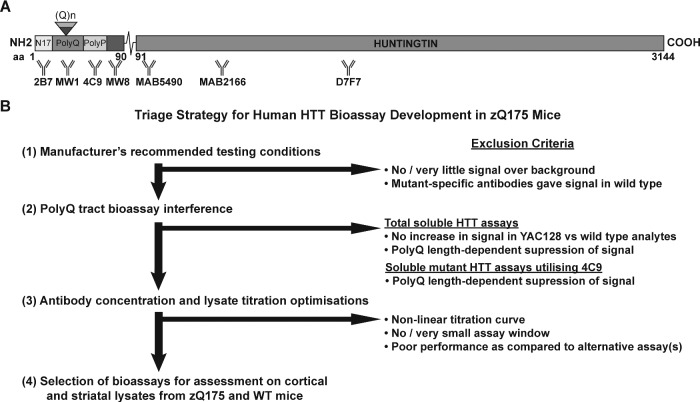
**Triage strategy employed to develop bioassays to detect soluble and aggregated species of HTT.** (**A**) Schematic diagram showing the relative location of HTT antibodies. Four antibodies detect exon 1 HTT: 2B7 maps to within the first 17 amino acids, MW1 detects expanded polyQ tracts, 4C9 detects the proline-rich region that lies between the two proline repeats in human HTT (but not mouse HTT) and MW8 acts as a neo-epitope antibody to the C-terminus of exon 1 HTT. MAB5490 maps to 115–129 amino acids, MAB2166 maps to 443–457 amino acids and D7F7 maps to an epitope containing the proline residue at 1220 amino acids. (**B**) The sequential triage strategy for selecting the optimal assays. *Stage 1*. Antibody pairings, in both orientations, were tested in cortical lysates from zQ175 and wild-type mice at 2, 6 and 12 months of age on the HTRF, AlphaLISA and MSD platforms using the manufacturer’s recommendations. The signals were grouped into those that tracked with: (i) soluble mutant HTT, (ii) aggregated HTT, (iii) soluble wild-type and mutant HTT or (iv) gave no signal, or a very low signal and were excluded from further analysis. *Stage 2*. Antibody combinations that detected ‘total soluble full-length HTT’, and those that used the 4C9 antibody in a ‘soluble mutant HTT assay’, were tested to determine whether the expanded polyQ tract interfered with the signal. The antibody pairings were tested in cortical lysates from heterozygous and homozygous *Hdh*Q20 mice, heterozygous and homozygous zQ175 mice, transgenic YAC128 mice and their wild-type counterparts. For ‘soluble mutant HTT’ assays, antibody combinations that gave lower signals in the zQ175 lysates as compared to the *Hdh*Q20, were excluded. For ‘total soluble full-length HTT’ assays, antibody combinations that gave a lower signal in the zQ175 lysates and did not give an increased signal in YAC128 lysates were excluded. *Stage 3*. The optimum antibody concentrations were established and these were used in 2-fold dilution series to determine whether, in the context of zQ175 cortical lysates, the assay signal would fall within the linear range of the titration curve. Criteria for exclusion were non-linear titration curves, a small assay window, or a poor assay performance compared to alternative assays. *Stage 4*. All optimized assays were finally run on cortical and striatal lysates from zQ175 and wild-type mice at 2, 6 and 12 months of age. WT, wild type.

The antibodies used for HTRF, AlphaLISA and MSD are summarized in Supplementary Table 1, and their epitope locations on the HTT protein are shown in Fig. 1A.

### Protein lysate preparation

For individual brain tissues, a 10% (w/v) total protein homogenate was prepared in ice-cold bioassay buffer [phosphate-buffered saline (PBS), 1% Triton-X-100] with complete protease inhibitor cocktail tablets (Roche), by homogenizing three times for 30 s in Lysing matrix D tubes at 6.5 m/s (MP Biomedicals) in a Fast-Prep-24™ instrument (MP Biomedicals), and up to 10 μl lysate was aliquoted per well in triplicate for bioassay analysis. For aggregation assays, 10 μl crude lysate was used, whereas for soluble assays, 10 μl of supernatant was used after brief centrifugation at 3500×*g* for 10 min. For assay optimization, *n* = 4/genotype were generally used for the zQ175 and *Hdh*Q20 samples, *n* = 3/genotype for the N171-82Q samples and *n* = 2–4/genotype for the YAC128 samples. For the analysis of selected assays in zQ175 cortex and striatum at 2, 6 and 12 months of age, *n* = 4/genotype were used.

### Homogenous time-resolved FRET

Cortical or striatal homogenates to a final volume of 10 μl were pipetted in triplicate into a 384-well (pure white, low volume, conical) proxiplate (Greiner Bio-One). For standard starting HTRF assay conditions, a ratio of 1 ng donor (terbium cryptate) to 20 ng acceptor (Alexa-488 for 4C9 and d2 for all other antibodies) was added per well in 5 μl HTRF detection buffer (50 mM NaH_2_PO_4_, 0.2 M KF, 0.1% bovine serum albumin and 0.05% Tween-20) with complete protease inhibitor cocktail tablets (Roche). Plates were incubated for 1.5 h on an orbital shaker (250 rpm) at room temperature, before the 15 μl reaction was read using an EnVision (Perkin Elmer) plate reader. For optimization of antibody concentration, the terbium donor concentration was maintained at 1 ng/well, whilst titrating in anti-huntingtin d2 or Alexa-488 acceptor at either 1-, 2-, 5-, 10, 20- or 40 ng per well. Unless otherwise stated, lysate titrations were performed by diluting zQ175 homogenates with homogenates from age-matched wild-type mice. For d2 acceptor detection, HTRF parameters were mirror: LANCE/DELFIA 412, excitation filter: UV2 (TRF) 320 (111), emission filter: APC 665 (205), 2nd emission filter: Europium 615 (203), excitation: 100%, delay: 60μs, window time: 150μs, number of flashes: 100, 2nd detector flashes: 100, time between flashes: 2000μs. For Alexa-488 acceptor detection, HTRF parameters were mirror: LANCE/DELFIA 412, excitation filter: UV2 (TRF) 320 (111), emission filter: TRF 520 (275), 2nd emission filter: TRF 495 (276), excitation: 100%, delay: 150μs, window time: 800μs, number of flashes: 100, 2nd detector flashes: 100, time between flashes: 2000μs.

### Amplified luminescent proximity homogenous assay-linked Immunosorbant assay

Brain homogenates to a final volume of 10 μl were pipetted in triplicate into a 384-well (pure white, square) Alphaplate (Perkin Elmer). For standard starting AlphaLISA assay conditions, the following were added sequentially in AlphaLISA immunoassay buffer and incubated on an orbital shaker (250 rpm) at room temperature: 5 μl anti-huntingtin-conjugated acceptor beads (1 h), 5 μl antibody biotinylated anti-huntingtin antibody (1 h) and 5 μl of streptavidin Alpha donor beads (1 h). The final manufacturer’s recommended concentrations in this 25 μl assay per well were acceptor beads: 20 mg/ml, biotinylated antibody: 3 nM, Alpha–Streptavidin donor beads: 20 mg/ml. For optimization of antibody concentration, the acceptor bead concentration was maintained at 20 mg/ml per well, whilst titrating in biotinylated anti-huntingtin at either 0.1-, 0.3-, 1-, 3-, 10-, 30- or 100 nM per well. Unless otherwise stated, lysate titrations were performed by diluting zQ175 homogenates with homogenates from age-matched wild-type mice. For detection, plates were read on an EnVision (Perkin Elmer) plate reader, using the custom EnVision AlphaLISA assay protocol. AlphaLISA parameters were mirror: AlphaScreen 444, excitation: laser, emission filter: 570 (244), excitation time: 180 ms, total measurement time: 550 ms. To test for endogenous-free biotin interference, cortical lysate from 12-month-old zQ175 mice was subjected to a 7-point, 2-fold serial dilution from 10 to 0.16 μl with either age-matched wild-type lysate or with lysis buffer alone. Titrated samples were then assayed with the AlphaLISA TruHits kit (AL900D, Perkin Elmer), or AlphaLISA TruHits Biotin-Free kit (AL901D, Perkin Elmer) exactly according to the manufacturer’s recommendations, using the same custom EnVision AlphaLISA assay protocol.

### Meso Scale Discovery conjugation assay

Brain homogenates to a final volume of 10 μl were pipetted in triplicate into 96-well custom-printed MSD plates. MSD plates were run exactly according to the manufacturer’s recommended conditions. Briefly, wells were incubated by blocking for 1 h in PBS–bovine serum albumin buffer (PBS, 3% bovine serum albumin), then huntingtin proteins were captured for 2 h at the default MSD anti-huntingtin capture concentration: ∼2 mg/ml ± 15%, and detected for 1 h using anti-huntingtin Sulpho-tag: 1 μg/ml, on an orbital shaker (300 rpm) at room temperature. Between each step, wells were washed three times in PBS-Tween buffer (PBS, 0.05% Tween-20). For optimization of antibody concentration, the anti-huntingtin capture was maintained at the MSD default ∼2 mg/ml ± 15% per well, whilst titrating in Sulpho-tagged anti-huntingtin at either 0.15-, 0.45-, 1.5-, or 4.5 μg/ml per well. Unless otherwise stated, lysate titrations were performed by diluting zQ175 homogenates with homogenates from age-matched wild-type mice. For detection, 150 μl gold label read buffer was added per well, and read on an MSD plate reader (Meso Scale Discovery).

### Statistical analysis

Data were screened for outliers using Grubb’s Test (GraphPad Prism v8), and only one outlier was removed before between-group comparisons. Statistical analysis was performed with GraphPad Prism (v8) using either a one‐way or two‐way ANOVA with Bonferroni *post hoc* test. Graphs were prepared using GraphPad Prism (v8).

## Data availability

The authors confirm that all the data supporting the findings of this study are available within the article and its supplementary material. Raw data will be shared by the corresponding author on request.

## Results

In order to expand the repertoire of HTT bioassays, we began by assessing pairwise combinations of six antibodies: 2B7, MW1, 4C9, MW8, MAB5490 and MAB2166 (Fig. 1A). Four of these detect epitopes within the exon 1 HTT protein: 2B7 maps to within the first 17 amino acids, MW1 detects expanded polyQ tracts, 4C9 detects the proline-rich region that lies between the two polyproline repeats in human HTT (but not mouse HTT) and MW8 acts as a neo-epitope antibody to the C-terminus of exon 1 HTT on western blots (Landles *et al.*, 2010). MAB5490 and MAB2166 are C-terminal to exon 1 HTT, mapping between 115–129 and 443–457 amino acids, respectively. We applied a sequential triage strategy to identify assays that would detect the following HTT species: (i) all soluble mutant HTT, (ii) soluble exon 1 HTT, (iii) soluble mutant HTT (excluding exon 1 HTT), (iv) aggregated HTT and (v) total soluble full-length HTT (mutant and wild type) (Fig. 1B). This assay development work was performed using the zQ175 knock-in mouse model of Huntington’s disease, in which mouse exon 1 *Htt* has been replaced with a mutated version of human exon 1 *HTT* (Heikkinen *et al.*, 2012; Menalled *et al.*, 2012).

For stage 1 of the triage strategy, we began by testing pairwise antibody duplexes in cortical lysates from zQ175 and wild-type mice at 2, 6 and 12 months of age. Over this age range, the levels of soluble mutant HTT are known to decrease, and aggregated HTT to increase in the cortex of Huntington’s disease knock-in models (Baldo *et al.*, 2012). Assessment of each antibody pair in both orientations amounted to 30 combinations, which were tested on three platforms: HTRF, AlphaLISA and MSD, in each case, using the manufacturer’s recommendations (Supplementary Fig. 1). These results allowed us to broadly group the antibody pairs into four categories (Supplementary Table 2). (i) A signal tracked with soluble mutant HTT if it decreased in zQ175 lysates with age, and was absent in wild type. (ii) A signal tracked with aggregated HTT if it increased in zQ175 lysates with age, and was absent in wild type. (iii) Signals that were present in both the zQ175 and the wild-type lysates could potentially detect total soluble full-length HTT. (iv) Antibody pairings that gave very low signals were considered non-viable. Of the starting 30 combinations, 25 HTRF (Supplementary Fig. 1A, B), 25 AlphaLISA (Supplementary Fig. 1C, D) and 28 MSD (Supplementary Fig. 1E, F) potential assays were taken forward for further assessment.

###  

#### Assays to measure ‘total soluble mutant HTT’

The following criteria were used to identify antibody pairings that detect ‘total soluble mutant HTT’ (exon 1 HTT and soluble HTT): (i) the signal decreased in zQ175 lysates with age and was absent in wild type, (ii) the antibody pairings included either MW1 or 4C9 that were specific to mutant HTT (i.e. MW1, because it did not recognize the 7Q repeat in endogenous mouse HTT and 4C9, because it detects the proline-rich peptide present in human but not mouse HTT), (iii) the antibody pairings did not include MW8 which is specific to exon 1 HTT (see later section) and (iv) the antibody pairings did not include MAB5490 or MAB2166, as these would not detect the exon 1 HTT protein. The six antibody pairs that satisfied these criteria were 2B7 with MW1, 2B7 with 4C9 and MW1 with 4C9, in both orientations, totalling 18 assays on the three platforms. Of these, the HTRF assay MW1-4C9 (Supplementary Fig. 1A) and the AlphaLISA assay 4C9-MW1 (Supplementary Fig. 1C) had already been excluded in stage 1 of the triage process.

**Figure 2:**
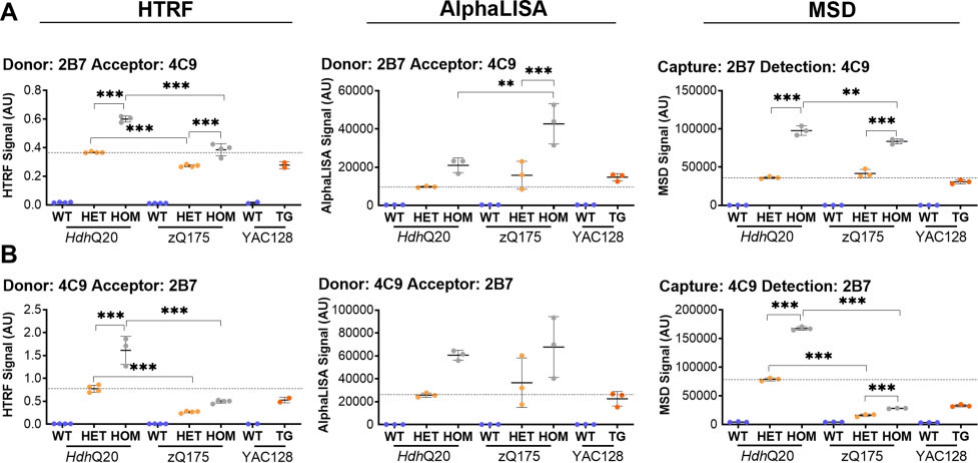
**Investigation of the effect of polyQ length on antibody pairings that include 4C9 for the detection of ‘total soluble mutant HTT’.** Antibody pairings of (**A**) 2B7-4C9 and (**B**) 4C9-2B7 were tested by HTRF, AlphaLISA and MSD using cortical lysates from heterozygous and homozygous *Hdh*Q20, heterozygous and homozygous zQ175 (*n* = 4/genotype) and YAC128 mice (*n* = 2–3/genotype) together with their respective wild-type littermate controls at 2 months of age. If the length of the polyQ repeat had no effect on assay performance, the signals for heterozygous *Hdh*Q20 and zQ175 lysates should be equivalent as should the signal for homozygous *Hdh*Q20 and zQ175 lysates. The length of the polyQ repeat clearly interfered with the signal in the 4C9-2B7 HTRF and MSD combinations and these were excluded from further analysis. Statistical analysis was two-way ANOVA with Bonferroni *post hoc* correction, mean ± SEM. *P ≤ 0.5, **P ≤ 0.01 and ***P ≤ 0.001. The test statistic, degrees of freedom and *P*-values for the ANOVA are listed in Supplementary Table 3. WT, wild type (blue); HET, heterozygote (orange); HOM, homozygote (grey); TG, transgenic (orange). Dotted line, signal in *Hdh*Q20 heterozygous lysates.

Next, stage 2 determined whether the length of the polyQ tract influenced the assay signal. Antibody pairings containing MW1 would generate a signal that varied, depending on the length of the polyQ tract, as the longer the polyQ, the more MW1 binding could occur. However, prior to further optimization, we investigated whether polyQ length might also influence the signal obtained from assays utilizing 2B7 and 4C9. Combinations of these two antibodies were tested in both orientations on all three platforms using the manufacturer’s recommended conditions in cortical lysates from heterozygous and homozygous *Hdh*Q20 knock-in mice, heterozygous and homozygous zQ175 knock-in mice, and transgenic YAC128 mice, together with their respective wild-type controls (Fig. 2). *Hdh*Q20 mice have a human exon 1 *HTT* with 18 CAGs (Wheeler *et al.*, 1999). For both the *Hdh*Q20 and the zQ175 knock-in lines, the signal was 2-fold higher in the homozygous as compared to the heterozygous lysates, as would be expected (Fig. 2). If the length of the polyQ repeat did not affect assay performance, the signal should have been equivalent in the *Hdh*Q20 and zQ175 heterozygotes, and similarly equivalent in the *Hdh*Q20 and zQ175 homozygotes. The 4C9-2B7 HTRF and MSD assays clearly did not fulfil these criteria, as the zQ175 signals were ∼20% of that in *Hdh*Q20, and were excluded at this stage (Fig. 2B). The remaining four assays were taken forward for the optimization of antibody concentration and lysate dilution in stage 3 of the triage process.

The level of soluble mutant HTT is known to decrease with age in the zQ175 brains, as a proportion is recruited into HTT aggregates. Therefore, we used cortical lysates from 2-month-old zQ175 and wild-type mice to optimize assay conditions, as this would present the maximum concentration of soluble mutant HTT available for detection. For each assay, the optimum antibody concentration was first determined (Supplementary Fig. 2). A serial 2-fold dilution of soluble mutant HTT was then performed, by titrating the zQ175 lysate with age-matched wild-type lysate (to keep the matrix constant), to predict under which conditions, a reduction in soluble mutant HTT concentration, from 2 to 12 months of age, would fall within the linear range (Supplementary Fig. 3). For HTRF, 2B7-MW1 and MW1-2B7 were not chosen, as the lysate dilutions for the optimum antibody concentrations did not result in a linear change in fluorescence (ΔF). For AlphaLISA and MSD, the 2B7-MW1 combination was chosen over MW1-2B7, for AlphaLISA, 4C9-2B7 was selected in preference to 2B7-4C9, and for MSD, 4C9-MW1 was selected in preference to MW1-4C9, as they gave wider and more linear assay windows (ΔF).

**Figure 3:**
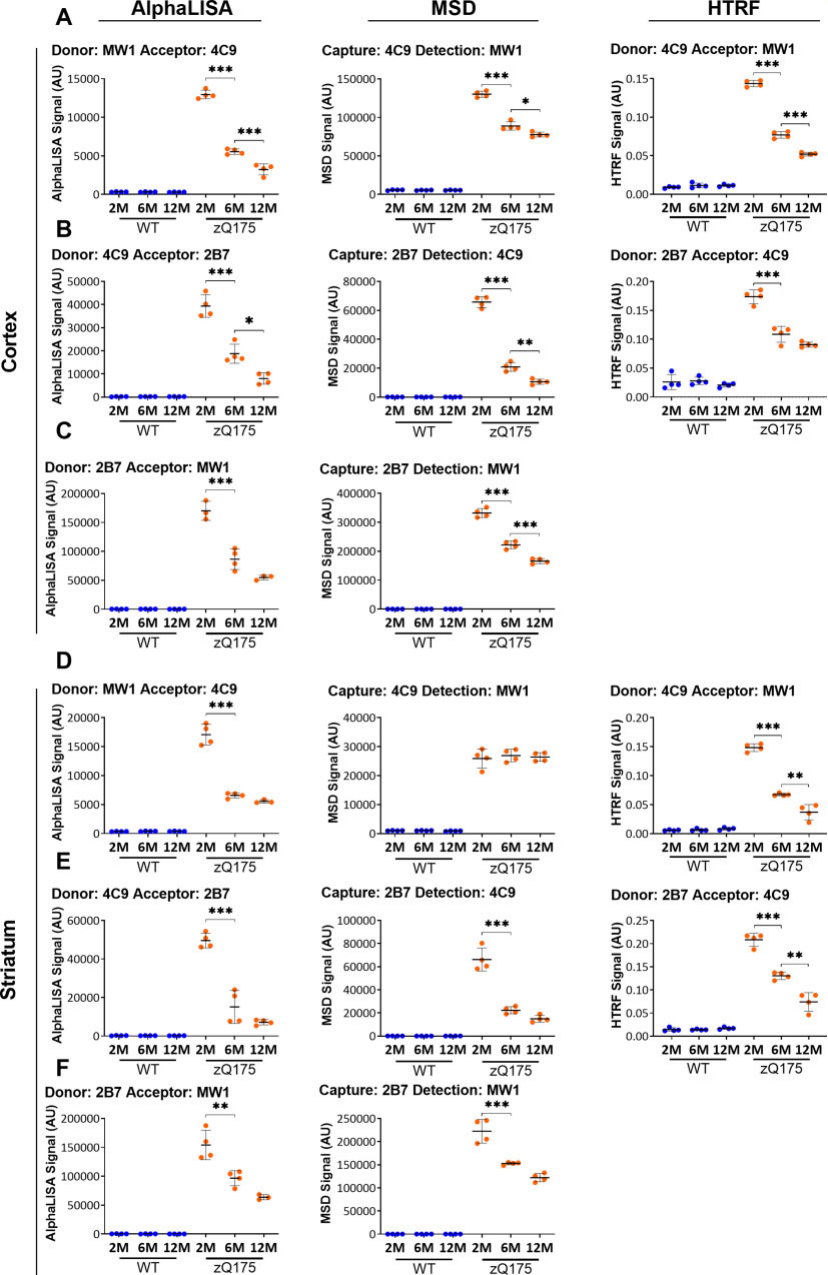
**Assessment of the ‘total soluble mutant HTT’ assays on the HTRF, AlphaLISA and MSD platforms in cortical and striatal lysates from zQ175 mice at 2, 6 and 12 months of age.** Antibody pairings of (**A, D**) 4C9 with MW1, (**B, E**) 4C9 with 2B7 and (**C, F**) 2B7 with MW1 were used to track changes in ‘total soluble mutant HTT’ by AlphaLISA, MSD and HTRF in cortical and striatal lysates from zQ175 and wild-type mice at 2, 6, and 12 months of age (*n* = 4/genotype). The level of ‘total soluble mutant HTT’ decreased with disease progression over this age range. Statistical analysis was one-way ANOVA with Bonferroni *post hoc* correction, mean ± SEM. *P ≤ 0.5, **P ≤ 0.01 and ***P ≤ 0.001. The test statistic, degrees of freedom and *P*-values for the ANOVA are listed in Supplementary Table 4. WT, wild type (blue); heterozygous zQ175 mice (orange).

The remaining eight assays were then used to track ‘total soluble mutant HTT’ levels in zQ175 and wild-type cortical and striatal lysates at 2, 6 and 12 months of age (Fig. 3). The optimum antibody combinations as shown in Supplementary Fig. 2 were used, but for practical reasons, 10 μl of undiluted lysate was used in all cases. A reduction in soluble mutant HTT was detected by all assays, except for 4C9-MW1 in the striatum, a result that we do not understand, but that was obtained in two independent experiments. These HTT bioassays are not quantitative and the changes with disease progression, observed here, cannot be interpreted as a fold-change. The estimated decline in soluble mutant HTT in the same cortical lysates differed between the eight graphs, and in order to interpret the data, the following criteria should be taken into consideration: (i) the linearity of the titration curve for the lysate dilution used, (ii) the proximity to the limit of detection on the titration curve and (iii) the presence of the antibody epitopes on other HTT isoforms that might be changing during disease progression and competing for the same antibody. Therefore, although not perfect, we would recommend using either 4C9-MW1 or 2B7-4C9 for HTRF, 2B7-MW1 for AlphaLISA and 2B7-MW1 or 4C9-MW1 for MSD (Fig. 3). For quantitative purposes, all of these assays would require polyQ-matched protein standards: this always applies to assays utilizing MW1, and we found that the recommended 2B7-4C9 HTRF assay exhibited some polyQ-length interference (Fig. 2).

#### Assays to measure the ‘soluble exon 1 HTT protein’

The MW8 antibody acts as an exon 1 HTT C-terminal neo-epitope antibody on western blots and we had previously published that it also acted as a neo-epitope antibody in an HTRF assay (Landles *et al.*, 2010). Therefore, to develop assays specific to ‘soluble exon 1 HTT’, we selected the antibody pairings, from stage 1 of the triage process that included MW8 and had also tracked with the soluble form of mutant HTT (Supplementary Fig. 1). These assays included combinations of 2B7 with MW8 or MW1 with MW8. The production of exon 1 HTT through incomplete splicing increases with CAG repeat length and does not occur in *Hdh*Q20 mice (Sathasivam *et al.*, 2013). Therefore, it was not possible to investigate the effect of polyQ length on assay performance by comparing signals in *Hdh*Q20 and zQ175 mice and replicating the approach used for the total soluble mutant HTT assays. Therefore, the triage process proceeded directly to stage 3, and the optimal antibody (Supplementary Fig. 4) and lysate concentrations (Supplementary Fig. 5) were determined using cortical lysates from 2-month-old zQ175 and age-matched wild-type mice as described above.

**Figure 4:**
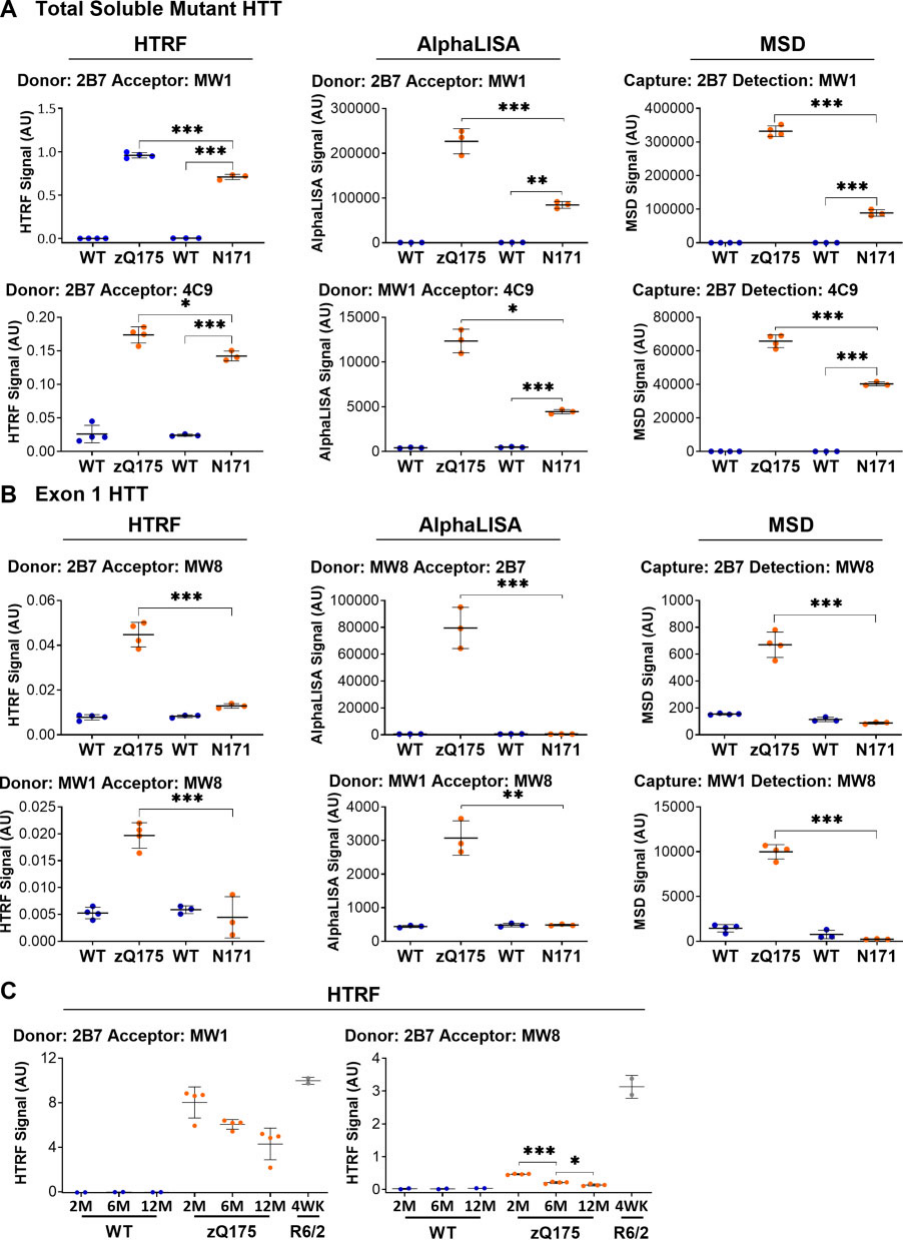
**Confirmation of the specificity of the ‘soluble exon 1 HTT’ assays.** (**A**) A selection of assays that detect ‘total soluble mutant HTT’ were tested in cortical lysates from zQ175 and N171-82Q mice and their respective wild-type controls at 2 months of age on the HTRF, AlphaLISA and MSD platforms. In all cases, a signal could be detected in both the zQ175 and the N171-82Q lysates (*n* = 3–4/genotype). (**B**) The ‘soluble exon 1 HTT’ assays were tested in the same lysates. In all cases, a signal was detected in the zQ175 but not the N171-82Q samples. As the N171-82Q mice are transgenic for a cDNA clone that does not contain intron 1, incomplete splicing cannot occur, and the exon 1 HTT protein cannot be generated. The absence of an exon 1 HTT signal in the N171-82Q is consistent with these assays specifically detecting only the exon 1 HTT protein (*n* = 3–4/genotype). (**C**) The 2B7-MW1 ‘total soluble mutant HTT’ and 2B7-MW8 ‘soluble exon 1 HTT’ HTRF assays were tested in cortical lysates from zQ175 (*n* = 4) and wild-type (*n* = 2) mice at 2, 6 and 12 months of age together with that from 4-week-old R6/2 mice (*n* = 2). Although the level of ‘total soluble mutant HTT’ in zQ175 lysate at 2 months was similar to that of ‘exon 1 HTT’ in R6/2 mice at 4 weeks, the level of ‘exon 1 HTT’ was much lower in the zQ175 cortex. Statistical analysis was two-way ANOVA with Bonferroni *post hoc* correction, mean ± SEM. *P ≤ 0.5, **P ≤ 0.01 and ***P ≤ 0.001. The test statistic, degrees of freedom and *P*-values for the ANOVA are listed in Supplementary Tables 5 and 6. WT, wild type (blue); heterozygous zQ175 mice (orange); N171, N171-82Q (orange); R6/2 mice (grey).

The exon 1 HTT protein is generated by the translation of the *Httexon1* transcript that is produced by an incomplete splicing event between exon 1 and exon 2 of *Htt* in knock-in mouse tissues (Sathasivam *et al.*, 2013). Therefore, this fragment should not be present in the brains of mice that are transgenic for an *Htt* cDNA, which does not contain introns. We utilized the N171-82Q mice (Schilling et al., 1999) that are transgenic for a cDNA construct, to test whether these assays were specific for exon 1 HTT. To ensure that soluble mutant HTT could be detected in the N171-82Q mice, we began by testing two of the ‘total soluble mutant HTT’ assays on each of the three platforms in cortical lysates from zQ175 and N171-82Q mice at 2 months of age. For each of the two assays, we detected a signal in lysates from both mouse models (Fig. 4A). Next, we tested two optimized ‘soluble exon 1 HTT’ assays for each platform. In contrast to the ‘total soluble mutant HTT’ assays, these only gave a signal in the zQ175 cortical lysates, but not in those from the N171-82Q mice (Fig. 4B). Given that exon 1 HTT cannot be generated in the N171-82Q mice by incomplete splicing, this is consistent with these assays being specific for the exon 1 HTT protein. Finally, we compared the levels of total ‘soluble mutant HTT’ and ‘soluble exon 1 HTT’ in cortex from R6/2 mice at 4 weeks of age with that from zQ175 mice at 2, 6 and 12 months, using the HTRF 2B7-MW1 and 2B7-MW8 assays (Fig. 4C). R6/2 mice are transgenic for a genomic fragment that encodes exon 1 HTT, and therefore this comprises all of the mutant HTT protein generated in R6/2 mice. Total ‘soluble mutant HTT’ levels in the cortex of 2-month-old zQ175 mice were relatively comparable to those in 4-week-old R6/2 mice, however, as it would be predicted, the level of ‘soluble exon 1 HTT’ was much lower in the zQ175 cortex, consistent with the slower rate of disease progression in the knock-in mice (Fig. 4C).

**Figure 5:**
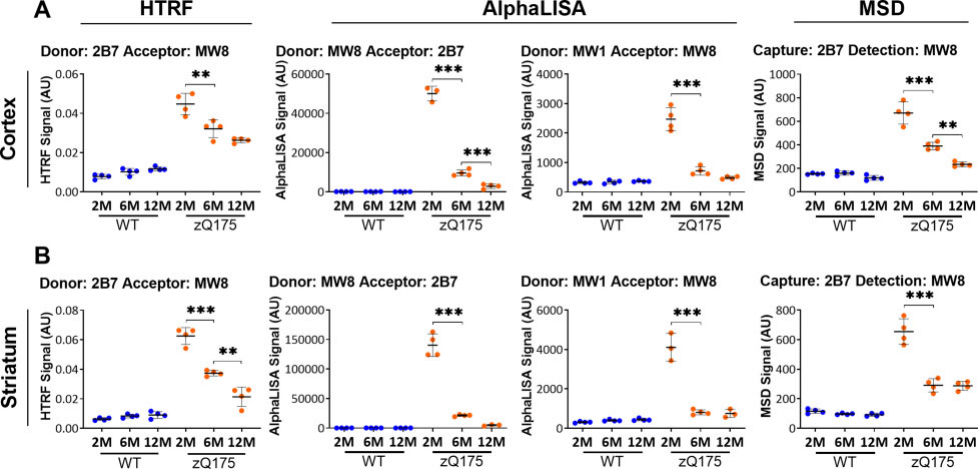
**Assessment of the ‘soluble exon 1 HTT’ assays on the HTRF, AlphaLISA and MSD platforms in cortical and striatal lysates from zQ175 mice at 2, 6 and 12 months of age.** The optimized ‘soluble exon 1 HTT’ assays were tested on the HTRF, AlphaLISA and MSD platforms in (**A**) cortical and (**B**) striatal lysates from zQ175 and wild-type littermates at 2, 6 and 12 months of age (*n* = 4/genotype). The level of exon 1 HTT at 6 and 12 months of age is close to, or below the limit of detection for the AlphaLISA assays and the 2B7-MW8 MSD assay in striatal lysate. Statistical analysis was one-way ANOVA with Bonferroni *post hoc* correction, mean ± SEM. *P ≤ 0.5, **P ≤ 0.01 and ***P ≤ 0.001. The test statistic, degrees of freedom and *P*-values for the ANOVA are listed in Supplementary Table 7. WT, wild type (blue); heterozygous zQ175 mice (orange).

The assays that gave the wider and more linear assay windows (ΔF) (Supplementary Fig. 5) in stage 3 of the triage process were then selected for testing in cortical and striatal lysates from zQ175 and wild-type mice at 2, 6 and 12 months of age (Fig. 5). These were 2B7-MW8 for HTRF, MW8-2B7 and MW1-MW8 and for AlphaLISA, and 2B7-MW8 for MSD. In all cases, the signal decreased with disease progression (Fig. 5).

For the AlphaLISA assay, by 12 months of age we noted that ‘soluble exon 1 HTT’ was below the lower limit of detection (Fig. 5). Therefore, since the AlphaLISA donor beads are coated with streptavidin, which may bind endogenous free-biotin in the tissue sample (as well as the intended biotinylated antibody), we reasoned that our AlphaLISA signal may be artificially reduced due to the presence of endogenous biotin. Therefore, to test for the presence of endogenous free-biotin, the AlphaLISA signals obtained for zQ175 cortical lysate from 12-month-old mice were compared using the AlphaLISA TruHits and AlphaLISA TruHits Biotin-Fee kits. The anti-biotin–streptavidin donor-beads used in the True-Hits Kit have been replaced with anti-digoxigenin-Fab donor beads that bind digoxigenin-labelled antibodies in the True-Hits Biotin-free Kit. This comparison demonstrated that brain lysates did contain endogenous free-biotin, and that the AlphaLISA signals should be restored through the use of the digoxigenin-labelled antibodies (Supplementary Fig. 6).

#### Assays to measure ‘soluble mutant HTT’, excluding exon 1 HTT

The MW1 and 4C9 antibodies are specific to mutant HTT in zQ175 mice and do not detect the wild-type protein. Therefore, antibody combinations utilizing either MW1 or 4C9, paired with antibodies detecting HTT epitopes that are C-terminal to exon 1 HTT, should provide assays to detect the levels of ‘soluble mutant HTT’ that exclude the ‘exon 1 HTT protein’. For stage 2 of the triage process, we determined whether the length of the polyQ tract interfered with assay performance for combinations of 4C9 with MAB5490 or 4C9 with MAB2166, in both orientations, on all three platforms (Supplementary Fig. 7). As before, antibody combinations were tested in cortical lysates from heterozygous and homozygous *Hdh*Q20 knock-in mice, heterozygous and homozygous zQ175 knock-in mice, and YAC128 mice, together with their respective wild-type controls. Due to incomplete splicing, we would expect that the level of full-length HTT generated from the mutant allele in the zQ175 might be lower than in *Hdh*Q20 mice. However, the dramatic extent to which the signal was decreased in the zQ175 samples, led us to conclude that all 12 assays exhibited polyQ-length interference and they were excluded from further analysis (Supplementary Fig. 7).

**Figure 6:**
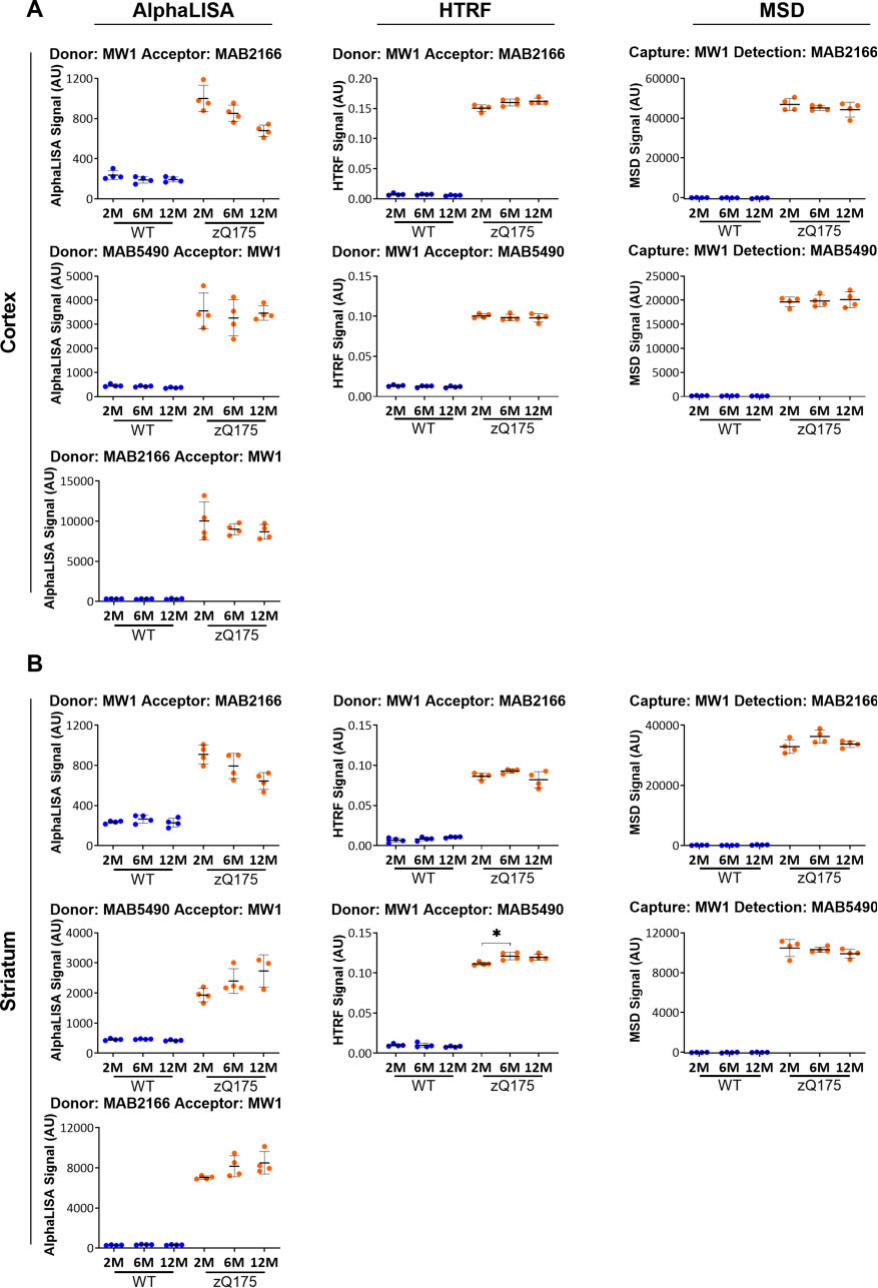
**Assessment of the ‘soluble mutant HTT’ (excluding exon 1 HTT) assays on the HTRF, AlphaLISA and MSD platforms in cortical and striatal lysates from zQ175 mice at 2, 6 and 12 months of age.** Assays utilizing antibody pairings of MW1 with either MAB2166 or MAB5490 on the AlphaLISA, HTRF and MSD platforms were used to track changes in ‘soluble mutant HTT’ (excluding exon 1 HTT) in (**A**) cortical and (**B**) striatal lysates from zQ175 and wild-type mice at 2, 6, and 12 months of age (*n* = 4/genotype). The level of ‘soluble mutant HTT’ (excluding exon 1 HTT) remained unchanged during disease progression over this age range. Statistical analysis was one-way ANOVA with Bonferroni *post hoc* correction, mean ± SEM. *P ≤ 0.5, **P ≤ 0.01 and ***P ≤ 0.001. The test statistic, degrees of freedom and *P*-values for the ANOVA are listed in Supplementary Table 8. WT, wild type (blue); heterozygous zQ175 mice (orange).

We proceeded to optimize the antibody combinations between MW1 and MAB5490 or MW1 and MAB2166, in both orientations and the optimal antibody (Supplementary Fig. 8) and lysate concentrations (Supplementary Fig. 9) were determined in cortical lysates from 2-month-old zQ175 and age-matched wild-type mice as described above. These assays were then tested in cortical and striatal lysates from zQ175 and wild-type mice at 2, 6 and 12 months of age using the optimal antibody concentration and 10 μl lysate (Fig. 6 and Supplementary Fig. 10). In all cases, a signal could be seen in the zQ175 lysates and not in wild type as it would be expected for assays that detect soluble mutant HTT. When MW1 was the donor antibody for the HTRF assays or the capture antibody for the MSD assays, there was no change in the level of ‘soluble mutant HTT’ (excluding soluble exon 1 HTT) between 2 and 12 months of age in the zQ175 cortex, a result that was supported by all of the AlphaLISA assays (Fig. 6). MW1 specifically detects soluble HTT, and in these HTRF and MSD assays, MW1 binds to soluble mutant HTT, and the use of MAB5490 or MAB2166 as the acceptor or detector, determines the proportion of soluble mutant HTT proteins that extend beyond exon 1 HTT. In contrast, the use of either MAB5490 or MAB2166 as the donor or capture antibodies for HTRF or MSD gave a different pattern, mostly likely because of competition with aggregated HTT (Supplementary Fig. 10). We recommend using the MW1-MAB2166 or MW1-MAB5490 assays for HTRF (at a lower lysate concentration than used here), MAB2166-MW1 for AlphaLISA and either MW1-MAB2166 or MW1-MAB5490 for MSD. All of these assays utilize MW1, and, therefore, would require polyQ-matched protein standards for quantitative purposes. We interpret these data to indicate that the level of soluble mutant HTT (excluding exon 1 HTT) does not change during the course of disease from 2 to 12 months of age in zQ175 cortex or striatum.

#### Assays to measure aggregated HTT

**Figure 7:**
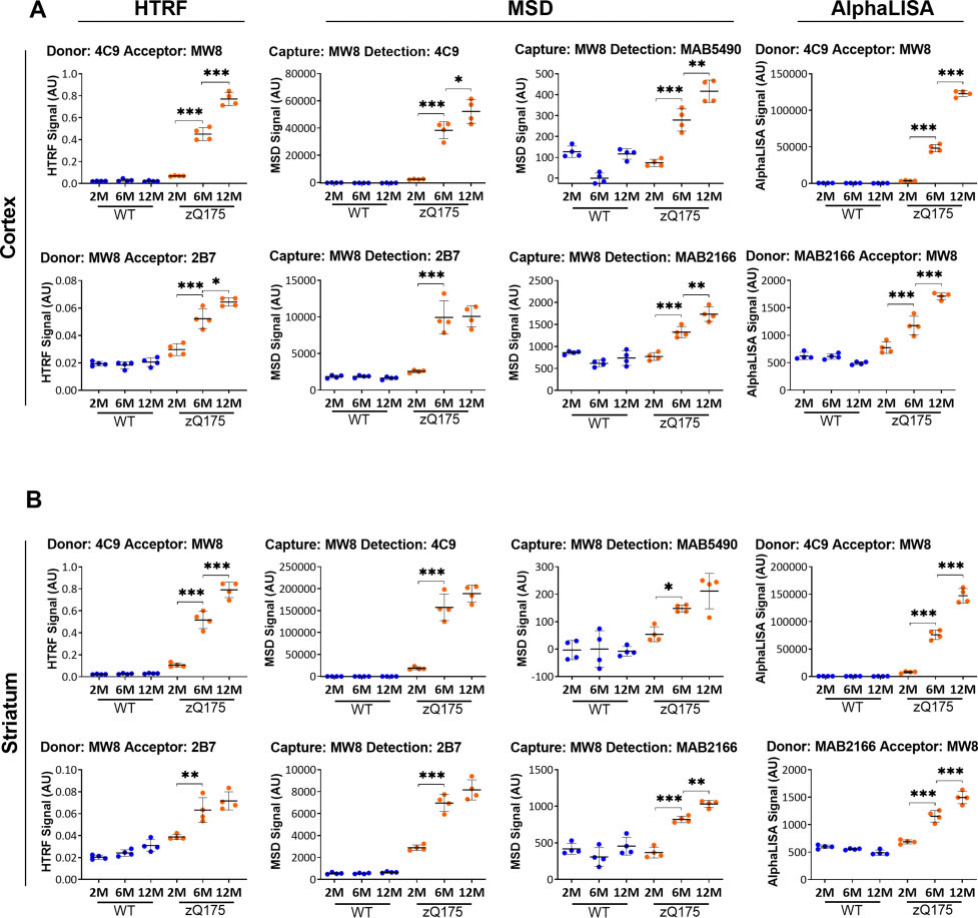
**Assessment of the ‘HTT aggregation’ assays on the HTRF, AlphaLISA and MSD platforms in cortical and striatal lysates from zQ175 mice at 2, 6 and 12 months of age.** Assays utilizing antibody combinations of MW8 with either 2B7, 4C9, MAB2166 or MAB5490 on the AlphaLISA, HTRF and MSD platforms were used to track changes in ‘aggregated HTT’ in (**A**) cortical and (**B**) striatal lysates from zQ175 and wild-type mice at 2, 6, and 12 months of age (*n* = 4/genotype). The level of ‘aggregated HTT’ increased during disease progression over this age range. Statistical analysis was one-way ANOVA with Bonferroni *post hoc* correction, mean ± SEM. *P ≤ 0.5, **P ≤ 0.01 and ***P ≤ 0.001. The test statistic, degrees of freedom and *P*-values for the ANOVA are listed in Supplementary Table 9. WT, wild type (blue); heterozygous zQ175 mice (orange).

The MW8-4C9 MSD assay for detecting aggregated HTT in tissue lysates is well established (Reindl *et al.*, 2019), and in our preliminary analysis, both the MW8-4C9 and the 4C9-MW8 pairings tracked with the accumulation of aggregated HTT in zQ175 mice between 2 and 12 months of age on all three platforms (Supplementary Fig. 1). In addition to these, we identified several other antibody pairings for which the signal was absent from wild-type mice, and increased from 2 to 12 months in zQ175 mice, the most sensitive of which appeared to be: MW8-2B7 for HTRF, MAB5490-MW8 and MAB2166-MW8 for AlphaLISA, and MW8-2B7, MW8-MAB5490 and MW8-MAB2166 for MSD (Supplementary Fig. 1 and Supplementary Table 2). The optimum antibody concentration was determined for these assays (Supplementary Fig. 11). Cortical lysates from 12-month-old zQ175 mice, that would contain the highest concentration of aggregated HTT over the age range under investigation, were subjected to 2-fold serial dilutions with wild-type lysates, to predict under which conditions, an increase in aggregated HTT from 2 to 12 months of age would fall within the linear range (Supplementary Fig. 12). The MW8 and 4C9 antibody pairing with the greatest near-linear assay window was selected for each platform. These assays were then tested, together with the other potential aggregation assays in cortical and striatal lysates from zQ175 and wild-type mice at 2, 6 and 12 months of age using the optimal antibody concentration and 10 μl lysate (Fig. 7). In all cases, specific assays tracked HTT aggregation, with comparable patterns in the change in signal with age, between cortex and striatum. We propose that the MW8-2B7 assays (HTRF and MSD) detected an aggregated form of HTT that occurs before the 2B7 epitope becomes inaccessible. The assays that utilized the MAB5490 or MAB2166 antibodies provided evidence for the recruitment of HTT fragments longer than exon 1 HTT into aggregates. Protein standards to quantify the aggregated HTT species that form *in vivo* do not exist.

#### Assays to measure ‘total soluble full-length HTT’ (mutant and wild type)

All assays developed so far have detected soluble or aggregated forms of mutant HTT. We next assessed the performance of assays to detect the levels of ‘total soluble full-length HTT’ (mutant and wild-type combined). Potential antibody pairings were 2B7 with either MAB5490 or MAB2166, or MAB5490 with MAB2166. Dependent on the location of the antibody epitopes, these antibodies pairings will detect full-length HTT as well as some proteolytic fragments. For stage 2 of the triage process, we first determined whether the length of the polyQ tract might interfere with assay performance. Antibody combinations of 2B7 with MAB5490, 2B7 with MAB2166 and MAB5490 with MAB2166 were tested in both orientations on all three platforms in cortical lysates from heterozygous and homozygous *Hdh*Q20 knock-in mice, heterozygous and homozygous zQ175 knock-in mice, and YAC128 mice, together with their respective wild-type controls at 2 months of age (Supplementary Fig. 13).

If these assays measure total soluble full-length HTT levels, the signal in the YAC128 mice should be greater than in wild type, as YAC128 contain three copies of the huntingtin gene (two endogenous mouse and one transgenic human). The signal should be comparable between wild-type, heterozygous *Hdh*Q20 and homozygous *Hdh*Q20 lysates, but may be decreased in the zQ175 lysates due to incomplete splicing. All 12 assays that used pairings with 2B7 either showed no increase in the YAC128 signal and/or showed a dramatically decreased signal in the zQ175 samples, that we attributed to polyQ-length interference, rather than resulting from a true reduction in zQ175 mutant HTT levels. This was also true for the MAB5490-MAB2166 MSD assay (Supplementary Fig. 13). Therefore, only five assays were taken forward to stage 3 of the triage process: MAB5490-MAB2166 and MAB2166-MAB5490 for both HTRF and AlphaLISA and MAB2166-MAB5490 for MSD, because the signal in the YAC128 sample was increased compared to wild-type and/or the reduction in the zQ175 signals as compared to *Hdh*Q20 was not as pronounced as for other assays (Supplementary Fig. 13).

We reasoned that assays that used antibody pairs that are located more C-terminal within the HTT protein, might be less likely to be influenced by the length of the polyQ repeat. Therefore, we assessed assays that used the D7F7 antibody, which detects an epitope located around the proline residue at amino acid 1220, in combination with 2B7, MAB5490 and MAB2166 in both orientations on the HTRF and MSD platforms. As before, these assays were subjected to stage 2 of the triage process, and tested in cortical lysates from heterozygous and homozygous *Hdh*Q20 knock-in mice, heterozygous and homozygous zQ175 knock-in mice, and YAC128 mice, together with their respective wild-type controls at 2 months of age (Supplementary Fig. 14). The assays selected to take forward for further optimization were MAB5490-D7F7, D7F7-MAB5490 and MAB2166-D7F7 for HTRF and D7F7-MAB5490 for MSD, based on the previous criteria.

Our strategy for the optimization of all of the soluble and aggregated mutant HTT assays had generated a mutant HTT titration curve by diluting the zQ175 lysate with lysate from age-matched wild-type animals. It is not possible to use this approach for assays designed to measure total soluble full-length HTT (mutant and wild type). Therefore, we tested the effect of titrating the concentration of total HTT by diluting lysates from 12-month-old zQ175 mice and age-matched controls in lysis buffer and running the 4C9-MW8 HTT aggregation assay on all three platforms (Supplementary Fig. 15). For HTRF and MSD, titration with lysis buffer had little effect on the titration curve when compared to dilution in wild-type lysate. However, it had a profound effect on the AlphaLISA signals, demonstrating that it would not be possible to establish a titration curve for ‘total soluble full-length HTT’ for the AlphaLISA assays (Supplementary Fig. 15).

**Figure 8:**
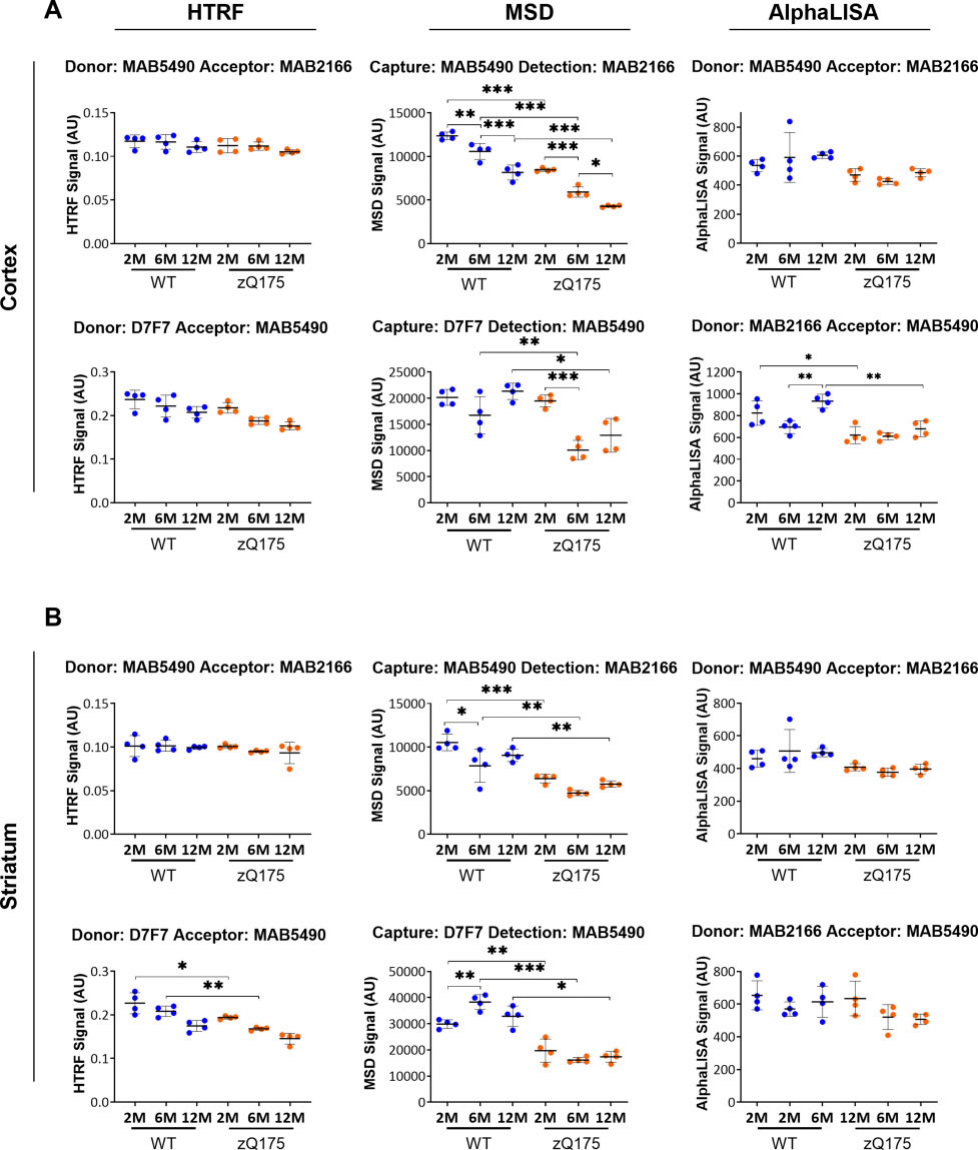
**Assessment of the ‘total soluble full-length HTT’ (mutant and wild type) assays on the HTRF, AlphaLISA and MSD platforms in cortical and striatal lysates from zQ175 mice at 2, 6 and 12 months of age.** The MAB5490-MAB2166 assays on all three platforms, the HTRF and MSD D7F7-MAB5490 and AlphaLISA MAB2166-MAB5490 assays were used to track ‘total soluble full-length HTT’ in (**A**) cortical and (**B**) striatal lysates from zQ175 and wild-type mice at 2, 6, and 12 months of age (*n* = 4/genotype). The HTRF and AlphaLISA assays indicated that the level of ‘total soluble full-length HTT’ was similar between wild-type and zQ175 mice, and did not change from between 2 and 12 months of age. Both of the MSD assays were subject to polyQ-length interference, and should not be used to compare total soluble HTT levels between zQ175 and wild-type mice. Statistical analysis was one-way ANOVA with Bonferroni *post hoc* correction, mean ± SEM. *P ≤ 0.5, **P ≤ 0.01 and ***P ≤ 0.001. The test statistic, degrees of freedom and *P*-values for the ANOVA are listed in Supplementary Table 10. WT, wild type (blue); heterozygous zQ175 mice (orange).

The optimal antibody concentrations were obtained for the ‘total soluble full-length HTT’ assays (Supplementary Fig. 16) and HTT was titrated by serial dilution in lysis buffer for the HTRF and MSD assays (Supplementary Fig. 17). The HTRF assays MAB2166-MAB5490, D7F7-MAB5490 and MAB2166-D7F7 were rejected based on the titration curves. The remaining HTRF and MSD assays, as well as MAB5490-MAB2166 and MAB2166-MAB5490 for AlphaLISA, were tested in cortical and striatal lysates from zQ175 and wild-type mice at 2, 6 and 12 months of age using the optimal antibody concentration and 10 μl of lysate (Fig. 8). We recommend using either of the two HTRF or two AlphaLISA assays. The two MSD assays: D7F7-MAB5490 and MAB5490-MAB2166 suffered from polyQ-length interference, as it had been predicted from the initial experiments (Supplementary Figs. 13 and 14), and should not be used for quantification.

## Discussion

Strategies aimed at lowering HTT protein levels, by targeting either the huntingtin gene or the transcript, have become a major focus of therapeutic development for Huntington’s disease (Tabrizi *et al.*, 2019*a*), with antisense oligonucleotides having progressed to a phase 3 clinical trial (Tabrizi *et al*., 2019*b*). Therefore, it is essential that we can measure soluble and aggregated forms of HTT, both for target validation and for pre-clinical studies in models of disease, and for pharmacodynamic readouts in clinical trials. Some HTRF and MSD assays are already widely used (Baldo *et al.*, 2012; Reindl *et al.*, 2019), but these do not distinguish all soluble HTT species. In the presence of an expanded CAG repeat, the incomplete splicing of exon 1 to exon 2 of *HTT* mRNA results in a small transcript that encodes the highly pathogenic and aggregation-prone exon 1 HTT protein, the levels of which increase with increasing CAG repeat length (Sathasivam *et al.*, 2013). Current assays that utilize 2B7-MW1 (and 2B7-4C9 in the context of the zQ175 mouse) to measure soluble mutant HTT do not distinguish between exon 1 HTT and full-length HTT. Given the pathogenic nature of exon 1 HTT, it is important that these isoforms can be tracked separately, and here we describe soluble HTT assays that are specific for (i) total soluble mutant HTT, (ii) soluble mutant exon 1 HTT, (iii) soluble mutant HTT (excluding exon 1 HTT) and (iv) total soluble full-length HTT (mutant the wild-type combined).

In all cases, the HTRF, AlphaLISA and MSD assays are quantitative only if it is possible to correlate the signal with a known protein standard. For soluble HTT assays, this is not straight forward. Fragments of the HTT protein are frequently used to generate a standard curve; however, it is not possible to maintain the solubility of smaller N-terminal fragments with the highly expanded polyQ repeats that are often present in our disease models (frequently > 100Q), because they aggregate so readily in solution (Scherzinger *et al.*, 1999). Because of this limitation, standard curves are frequently prepared with N-terminal fragments of HTT that carry much smaller polyQ expansions than they are present in the sample of interest (Reindl *et al.*, 2019). Here, we showed that many assays that detect ‘total soluble mutant HTT’ or ‘soluble mutant HTT’ (excluding exon 1HTT) suffer from polyQ-length interference, to the extent that the signal obtained in zQ175 mice (190Q) was dramatically less than for *Hdh*Q20 mice (20Q) at 2 months of age (Fig. 2 and Supplementary Fig. 7). This is likely to be further exacerbated by somatic instability, which is pronounced in the 12-month zQ175 striatum, with the consequence that the polyQ expansion in the tissue under investigation is longer than that initially measured in ear biopsies. Therefore, the commonly used HTT standards cannot be reliably used to determine the mass of a HTT analyte that carries a highly expanded polyQ repeat. However, these assays could be used to compare the levels of mutant HTT between mice with comparable CAG repeat expansions, or when assessing the reduction in mutant HTT in response to a HTT-lowering intervention.

We were not able to identify any ‘total soluble full-length HTT’ MSD assays that were not subject to profound polyQ-length interference, even when the two antibodies in question detected peptides located at ∼450 (MAB2166) and ∼1220 (D7F7) amino acids C-terminal to the polyQ repeat (Supplementary Figs. 13 and 14). Therefore, in a heterozygous situation, these assays could not be used for quantitative purposes, as a protein standard could not simultaneously control for two HTT proteins with different polyQ lengths. Their use for relative comparisons of total full-length HTT levels between samples might also be complicated, if the ratio in the levels of the wild-type and mutant HTT proteins is not equivalent between the samples under investigation.

The quantification of mutant HTT levels in CSF from Huntington’s disease patients is also a complex issue. The HTRF, AlphaLISA and MSD assays described here are not sufficiently sensitive to measure HTT in CSF. However, single-molecule counting assays have been developed that utilize the 2B7-MW1 antibody pair and these have estimated that there are femtomolar levels of mutant HTT in CSF, based on a HTT standard of 548 amino acids with 46Q (Wild *et al.*, 2015). The length of this polyQ tract is likely to be similar to the polyQ repeat found in Huntington’s disease patient’s blood. However, repeats of several hundred CAGs have been found in the brains of Huntington’s disease patients due to somatic CAG repeat expansion (Kennedy *et al.*, 2003); and the importance of this was recently brought into focus by the identification of genes that are known to modulate somatic instability, as genetic modifiers that drive the age of onset and disease progression for Huntington’s disease (Moss *et al.*, 2017; GeM-HD, 2019). Given that the 2B7-MW1 signal increases with increasing polyQ length, a higher signal could correspond to more HTT, or to longer polyQ tracts, which mutant HTT proteins in CSF might contain. An attempt to resolve this problem used two MSD assays by which the equivalent of 2B7-MW1 was normalized to an assay that detected full-length HTT (Aviolat *et al.*, 2019). This detailed study provided a means of calculating the average polyQ length in a sample, but it is likely that full-length HTT assays would be subject to polyQ-length interference, which may then affect the interpretation of these results for very long polyQ tracts.

The MW8 antibody detects a conformation-sensitive epitope. We have previously shown that, on western blots, MW8 acted as a neo-epitope antibody to the C-terminus of soluble exon 1 HTT, and did not detect the same peptide when part of a longer soluble HTT fragment or the full-length HTT protein (Landles *et al.*, 2010). However, MW8 did immunoprecipitate full-length HTT from Huntington’s disease knock-in mouse brain lysates (Landles *et al.*, 2010). We show here that when MW8 is used as the acceptor or detection antibody, and combined with either 2B7 or MW1 as the donor or capture antibody, it also acted as a neo-epitope antibody for the C-terminus of exon 1 HTT, and provided an assay for soluble exon 1 HTT on all three platforms. In keeping with the other soluble HTT assays, it is possible that 2B7-MW8 might also be subject to polyQ-length interference, although it was not possible to test this by comparing signals in *Hdh*Q20 and zQ175 lysates, as the level of incomplete splicing, and therefore of the exon 1 HTT protein, increases with increasing CAG repeat length (Sathasivam *et al.*, 2013).

Bioassays to detect aggregated HTT have previously been developed, and include the MW8-4C9 MSD assay (Reindl *et al.*, 2019) as well as duplexes in which an antibody is paired with itself (e.g. 4C9-4C9 or MW8-MW8) (Baldo *et al.*, 2012). We have shown that assays that pair 4C9 with MW8 in both orientations effectively track with aggregation of human HTT on all three platforms. Because the 4C9 antibody is human-specific, complementary assays will need to be developed for mouse HTT aggregates. In addition, our comprehensive investigation of antibody pairings has identified additional assays for which the signal increased from 2 to 12 months of age in zQ175 mice and might be predicted to be tracking the HTT aggregation process. Generally, when MW8 was used as the donor/capture antibody, it resulted in an assay that tracked with aggregation (Supplementary Table 2). The MW8-MW1 antibody pairing failed to provide an assay on all three platforms, consistent with the epitope for MW1 being lost during the aggregation process due to the formation of the cross-β-sheet structure between glutamine tracts (Scherzinger *et al.*, 1997). The MW8-2B7 assay generated a signal that accumulated with age in zQ175 mice on both the HTRF and the MSD platforms. The 2B7 antibody does not readily bind to aggregated HTT (Baldo *et al.*, 2012). Therefore, the MW8-2B7 assays may detect a HTT species early in the aggregation pathway, after the polyQ repeat has become inaccessible, but whereas the 2B7 epitope is still exposed. This HTT aggregation species mostly accumulated between 2 and 6 months of age, and would be consistent with this hypothesis. Assays that utilize 2B7, 4C9 or the MW8 antibodies detect HTT aggregates containing exon 1 HTT, and by immunohistochemistry, inclusions have not been detected with antibodies more C-terminal to exon 1 HTT in mouse models (REM, unpublished data) or brains of patients with Huntington’s disease (Schilling *et al.*, 2007). However, MW8 in some pairings with MAB5490 or MAB2166 on the AlphaLISA and/or MSD platforms clearly generated signals that tracked with HTT aggregation and may provide evidence of HTT fragments longer than exon 1 HTT being recruited into aggregates. However, the extent to which this occurs does not impact on the steady-state levels of full-length mutant HTT (Fig. 6).

We have performed a comprehensive analysis to develop novel assays to track soluble and aggregated HTT species, and, in the absence of polyQ-matched standards, to determine how these might be used in combination with existing assays. This analysis was developed to track HTT in tissues from the zQ175 mouse, with ∼190 glutamines, aged from 2 to 12 months. Optimization of potential assays was performed in 2-month cortical lysate for assays that detected soluble HTT, and in 12 month lysates for those that detected aggregated HTT, the ages at which, for this study, the maximum concentrations of these HTT species would be present. The optimal antibody concentrations were first determined, followed by serial dilutions of the zQ175 lysate with wild-type lysate, to provide a titration curve over a range of mutant HTT concentrations. Titration in lysis buffer had to be used for the ‘total soluble HTT’ assays, an approach not possible for AlphaLISA, for which changing the lysate matrix dramatically changed the performance of the assay (Supplementary Fig. 15). Assays were selected based on the linearity of the titration curve, the signal-to-noise ratio and assay window. This strategy can now be used to optimize selected assays for tracking soluble or aggregated HTT in other mouse models of Huntington’s disease, or cell-based systems, for which a polyQ-matched protein standard does not exist. It is worth noting that for AlphaLISA assays, the use of digoxigenin-labelled antibodies (as an alternative to biotinylated antibodies) should be used for tissue lysates. In this study, we used four mice per genotype because so many assays were being evaluated simultaneously, but it would recommend increasing this to six per genotype for specific HTT studies.

## Conclusion

In summary, we present assays that detect (i) total soluble mutant HTT, (ii) soluble exon 1 HTT, (iii) soluble mutant HTT (excluding exon 1 HTT), (iv) aggregated HTT and (v) total soluble full-length HTT (mutant and wild type) (Table 1). None of these assays can be used to quantify the amount of a HTT species, in the absence of a polyQ-length-matched HTT standard. We tracked soluble and aggregated HTT in cortical and striatal lysates from the zQ175 mouse model of Huntington’s disease. The level of exon 1 HTT (2B7-MW8) decreased with disease progression as it was recruited into HTT aggregates. The MW8-2B7 assays most likely detect a HTT species that forms early in the aggregation process, HTT aggregation accumulates from 2 to 12 months of age (4C9-MW8) and recruits longer HTT fragments (MW8-MAB5490, MW8-MAB2166). The level of soluble mutant HTT (excluding exon 1 HTT), remained unchanged, suggesting that, of total mutant HTT, it is exon 1 HTT that is mostly recruited into aggregates. However, these are complex assays that depend on the avidity of two antibodies, which are likely to detect epitopes in more than one soluble or aggregated isoform, the relative levels of which are changing with disease progression. They may also detect epitopes that can be masked by HTT-binding proteins, and interactions that may change during the disease process. Therefore, many factors need to be considered when interpreting the data generated by these bioassays.

**Table 1:** Selected assays for tracking soluble and aggregated species of HTT

Analyte	HTRF	AlphaLISA	MSD
Total soluble mutant HTT	4C9-MW1 2B7-4C9	2B7-MW1	2B7-MW1 4C9-MW1
Soluble exon 1 HTT	2B7-MW8	MW8-2B7 MW1-MW8	2B7-MW8
Soluble mutant HTT (excluding exon 1 HTT)	MW1-MAB5490 MW1-MAB2166	MAB2166-MW1	MW1-MAB2166 MW1-MAB5490
Aggregated HTT	4C9-MW8 MW8-2B7	4C9-MW8 MAB2166-MW8	MW8-4C9 MW8-2B7 MW8-MAB5490 MW8-MAB2166
Total soluble full-length HTT (mutant and wild type)	MAB5490-MAB2166 D7F7-MAB5490	MAB5490-MAB2166 MAB2166-MAB5490	None

## Supplementary material

Supplementary material is available at *Brain Communications* online.

## Supplementary Material

fcaa231_Supplementary_Data

## Abbreviations

AlphaLISA =: amplified luminescent proximity homogeneous assay
CAG =: cytosine adenine guanine
DMSO =: dimethyl sulphoxide
ΔF =: change in fluorescence
HTRF =: homogeneous time-resolved fluorescence
MSD =: Meso Scale Discovery
PBS =: phosphate-buffered saline
PolyQ =: polyglutamine
Q =: glutamine
