# Supplementary material for: Development of novel bioassays to detect soluble and aggregated Huntingtin proteins on three technology platforms
Source: Brain Commun. 2021 Jan 5;3(1):fcaa231. doi: 10.1093/braincomms/fcaa231 (PMC7878250; doi:10.1093/braincomms/fcaa231)
Supplement: fcaa231_Supplementary_Data [file fcaa231_supplementary_data.pdf]

Running Title: Bioassays for soluble and aggregated huntingtin proteins

SUPPLEMENTARY MATERIAL

Development of novel bioassays to detect soluble and aggregated huntingtin proteins on three technology platforms

Christian Landles[1], Rebecca E. Milton[1], Alexandre Jean[2], Stuart McLarnon[2],

Sean J. McAteer[1], Bridget A. Taxy[1], Georgina F. Osborne[1], Chuangchuang Zhang[3],

Wenzhen Duan[3], David Howland[4] and Gillian P. Bates[1]

[1]Dept. Neurodegenerative Disease, Queen Square Institute of Neurology, UCL, London, UK.

[2]Perkin Elmer Inc., Seer Green, UK.

[3]Dept. Psychiatry and Behavioral Sciences, Dept. Neuroscience, Johns Hopkins University School of Medicine, Baltimore, USA.

[4]CHDI Management / CHDI Foundation Inc., New York, USA.

Correspondence to: Gillian Bates (PhD)
Huntington's Disease Centre, Department of Neurodegenerative Disease and UK Dementia Research Institute at UCL,
Queen Square Institute of Neurology, UCL,
Queen Square,
London, WC1N 3BG, UK.
Email: gillian.bates@ucl.ac.uk

Supplementary Table 1. Summary of Antibodies.

Name	Immunogen	Epitope	Species	Reference / Source
2B7	HTT peptide: aa 1-17		Mouse Monoclonal	(Weiss *et al.*, 2009) CHDI Foundation
MW1	HTT Exon1 (67Q)	PolyQ	Mouse Monoclonal	(Ko *et al.*, 2001) CHDI Foundation
4C9	HTT peptide: aa 51-71		Mouse Monoclonal	(Landles *et al.*, 2010) CHDI Foundation
MW8	HTT Exon1 (67Q)	aa 83-90	Mouse Monoclonal	(Ko *et al.*, 2001) CHDI Foundation
MAB5490	Recombinant HTT: aa 115-129		Mouse Monoclonal	Sigma-Aldrich, MAB5490
MAB2166	HTT fusion protein: aa 181-810	aa 443-457	Mouse Monoclonal	Sigma-Aldrich, MAB2166
D7F7	HTT peptide: residues surrounding Pro1220		Rabbit Monoclonal	Cell Signaling Technology #5656

References

Ko J, Ou S, Patterson PH. New anti-huntingtin monoclonal antibodies: implications for huntingtin conformation and its binding proteins. Brain Res Bull 2001; 56(3-4): 319-29.

Landles C, Sathasivam K, Weiss A, Woodman B, Moffitt H, Finkbeiner S*, et al.* Proteolysis of mutant huntingtin produces an exon 1 fragment that accumulates as an aggregated protein in neuronal nuclei in Huntington disease. J Biol Chem 2010; 285(12): 8808-23.

Weiss A, Abramowski D, Bibel M, Bodner R, Chopra V, DiFiglia M*, et al.* Single-step detection of mutant huntingtin in animal and human tissues: A bioassay for Huntington's disease. Anal Biochem 2009; 395(1): 8-15.

Supplementary Table 2. HTT species potentially detected by each antibody pair tested by HTRF, AlphaLISA, and MSD.

Antibody Donor / Capture	Antibody Acceptor / Detection	HTRF HTT Protein Species Detected	AlphaLISA HTT Protein Species Detected	MSD HTT Protein Species Detected
2B7	MW1	Soluble mutant	Soluble mutant	Soluble mutant
	4C9	Soluble mutant	Soluble mutant	Soluble mutant
	MW8	Soluble mutant	Soluble mutant	Soluble mutant
	MAB5490	Total full-length	Total full-length	Total full-length
	MAB2166	Total full-length	Total full-length	Total full-length
MW1	2B7	Soluble mutant	Soluble mutant	Soluble mutant
	4C9	No assay	Soluble mutant	Soluble mutant
	MW8	Soluble mutant	Soluble mutant	Soluble mutant
	MAB5490	Soluble mutant	No assay	Soluble mutant
	MAB2166	Soluble mutant	Soluble mutant	Soluble mutant
4C9	2B7	Soluble mutant	Soluble mutant	Soluble mutant
	MW1	Soluble mutant	No assay	Soluble mutant
	MW8	Aggregated	Aggregated	Aggregated
	MAB5490	Soluble mutant	Total full-length	Soluble mutant
	MAB2166	Soluble mutant	Soluble mutant	Soluble mutant
MW8	2B7	Aggregated	Soluble mutant	Aggregated
	MW1	No assay	No assay	No assay
	4C9	Aggregated	Aggregated	Aggregated
	MAB5490	No assay	No assay	Aggregated
	MAB2166	Aggregated	No assay	Aggregated
MAB5490	2B7	Total full-length	Total full-length	Total full-length
	MW1	Soluble mutant	Soluble mutant	Soluble mutant
	4C9	Soluble mutant	Soluble mutant	Soluble mutant
	MW8	No assay	Aggregated	No assay
	MAB2166	Total full-length	Total full-length	Total full-length
MAB2166	2B7	Total full-length	Total full-length	Total full-length
	MW1	Soluble mutant	Soluble mutant	Soluble mutant
	4C9	Soluble mutant	Soluble mutant	Soluble mutant
	MW8	No assay	Aggregated	Aggregated
	MAB5490	Total full-length	Total full-length	Total full-length

Supplementary Figure 1. Assessment of 30 pairwise combinations of six antibodies to detect soluble or aggregated HTT by HTRF, AlphaLISA or MSD in cortical lysates from zQ175 mice. All 30 pairwise combinations of antibodies: 2B7, MW1, 4C9, MW8, MAB5490 and MAB2166 were tested in both orientations using the HTRF, AlphaLISA and MSD platforms to assess HTT levels in cortical lysates from zQ175 and WT mice at 2, 6 and 12 months of age, using the manufacturer's recommended conditions (n = 3 - 4 / genotype). **(A)** The five antibody combinations with either 2B7, MW1 or 4C9 as the FRET donor on the HTRF platform. **(B)** The five antibody combinations with either MW8, MAB5490 or MAB2166 as the FRET donor on the HTRF platform. **(C)** The five antibody combinations with either 2B7, MW1 or 4C9 as the donor beads on the AlphaLISA platform. **(D)** The five antibody combinations with either MW8, MAB5490 or MAB2166 as the donor beads on the AlphaLISA platform. **(E)** The five antibody combinations with either 2B7, MW1 or 4C9 as the capture antibody on the MSD platform. **(F)** The five antibody combinations with either MW8, MAB5490 or MAB2166 as the capture antibody on the MSD platform. Statistical analysis was one-way ANOVA with Bonferroni *post hoc* correction, mean ± SEM. *$p \leq 0.5$, ** $p \leq 0.01$, ***$p \leq 0.001$. The test statistic, degrees of freedom and p values for the ANOVA are provided in Supplementary Tables 11 and 12. WT = wild-type.

Supplementary Figure 1 A

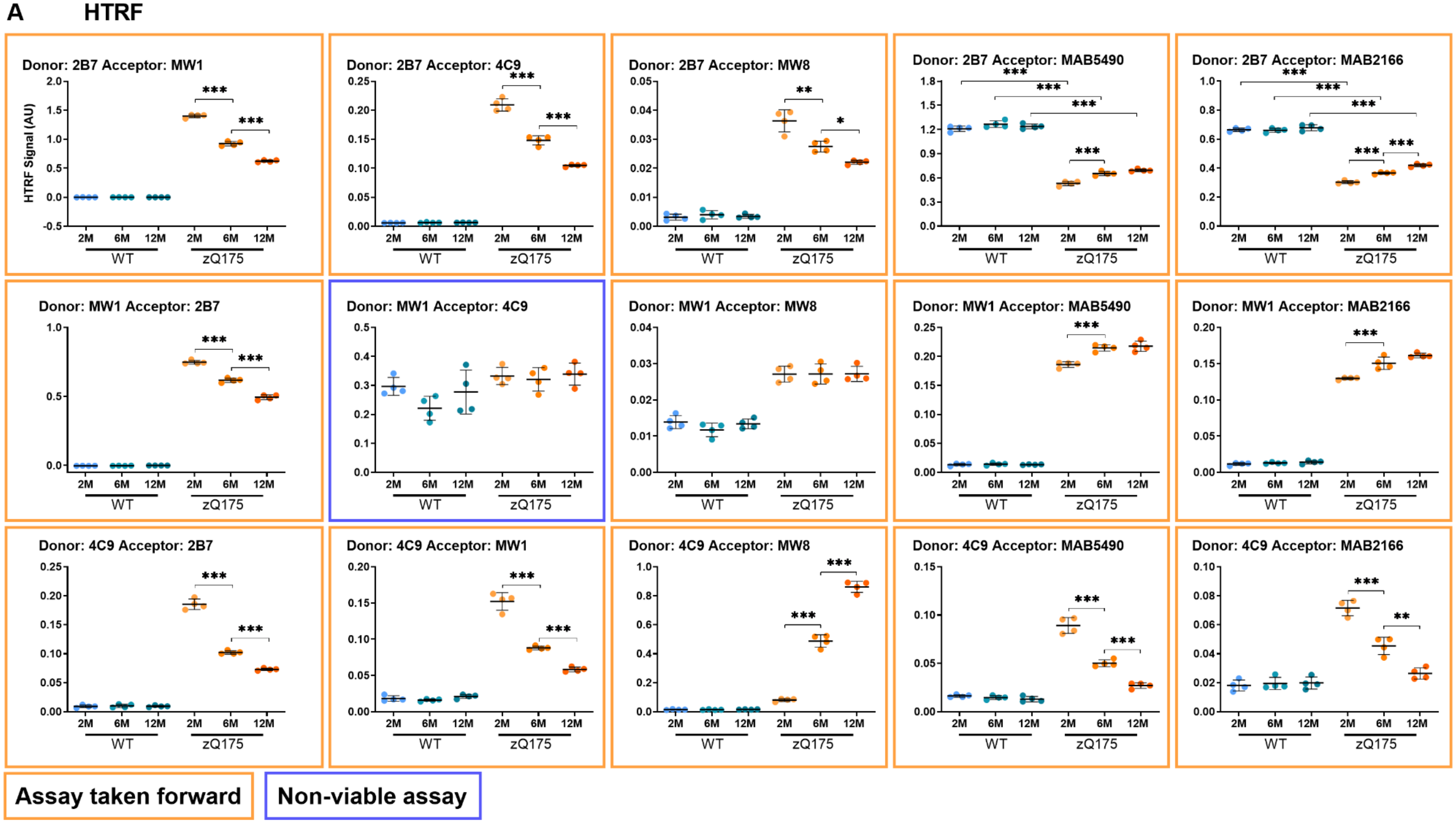

The MW1-4C9 antibody pairing is non-viable. MW1 is mutant specific and should not give the same signal in WT and zQ175 mice.

Supplementary Figure 1 B

B HTRF

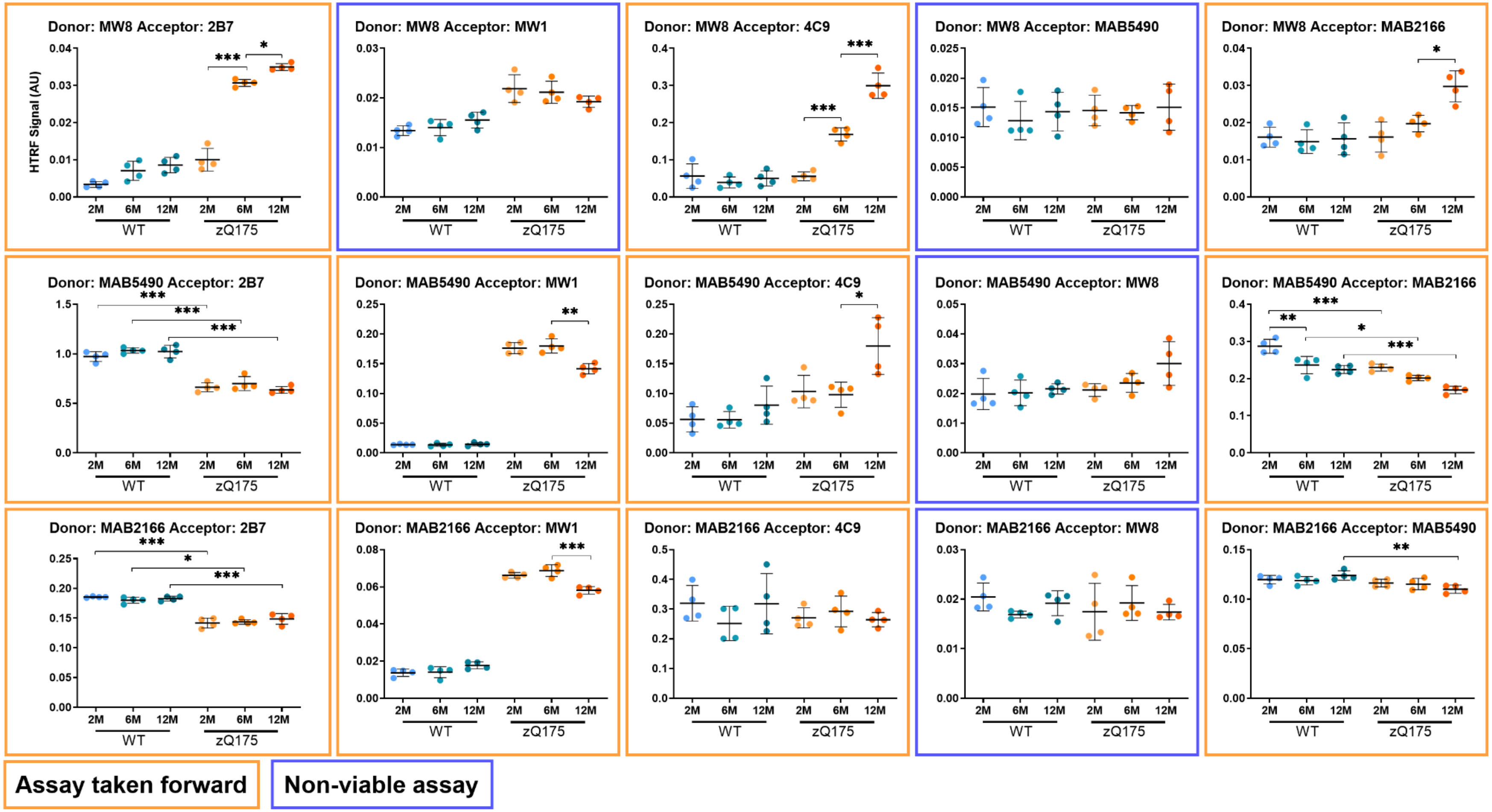

The MW8-MAB5490, MAB5490-MW8 and MAB2166-MW8 antibody pairings were considered non-viable due to the low signal to noise ratio as compared to alternative assays.

Supplementary Figure 1 C

C AlphaLISA

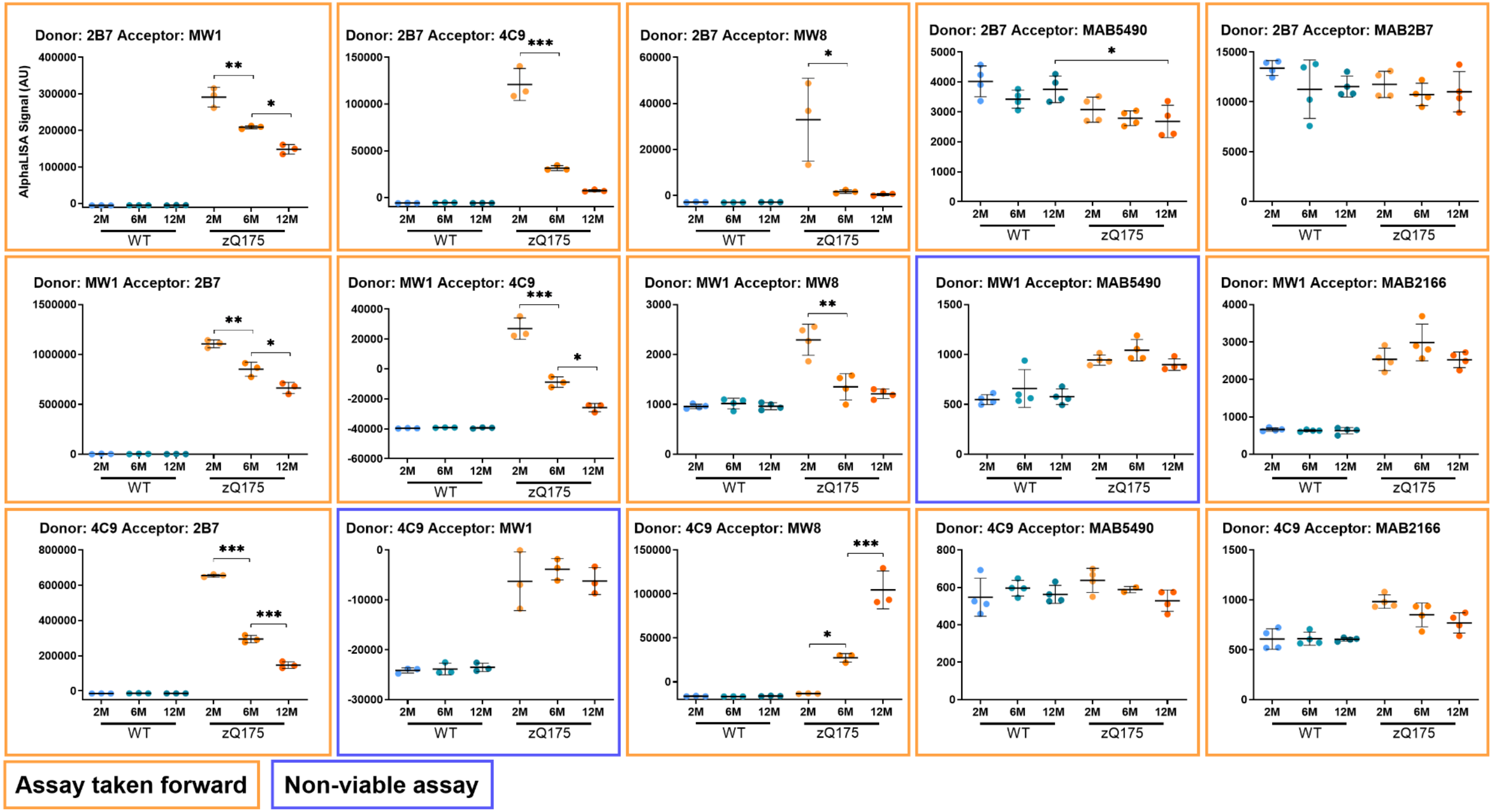

The MW8-MW1, MW8-MAB5490 and MW8-MAB2166 antibody pairings were considered non-viable due to the low signal to noise ratio as compared to alternative assays.

Supplementary Figure 1D

D AlphaLISA

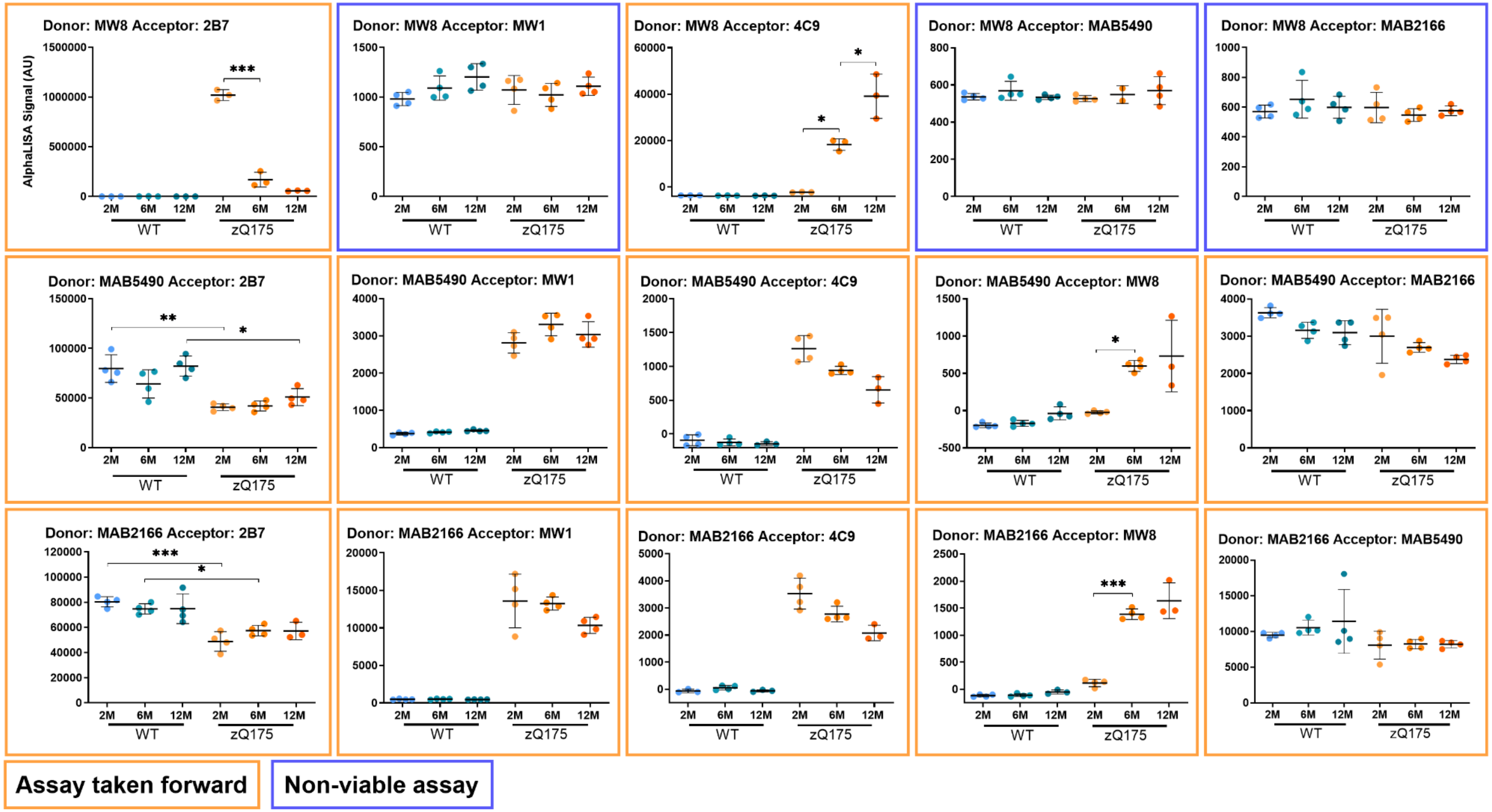

The MW8-MW1, MW8-MAB5490 and MW8-MAB2166 antibody pairings were considered non-viable due to the low signal to noise ratio as compared to alternative assays.

Supplementary Figure 1E

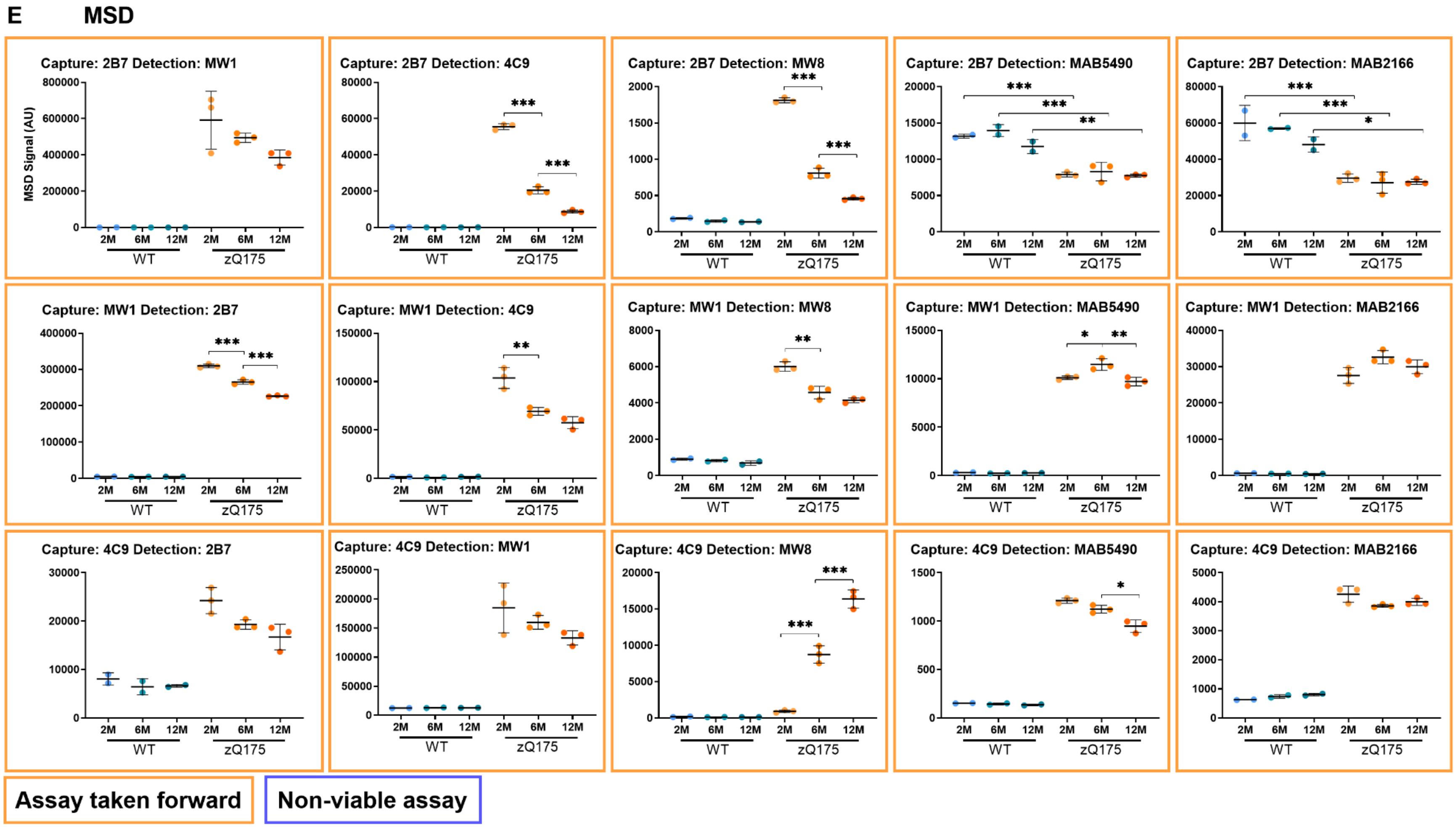

Supplementary Figure 1F

F MSD

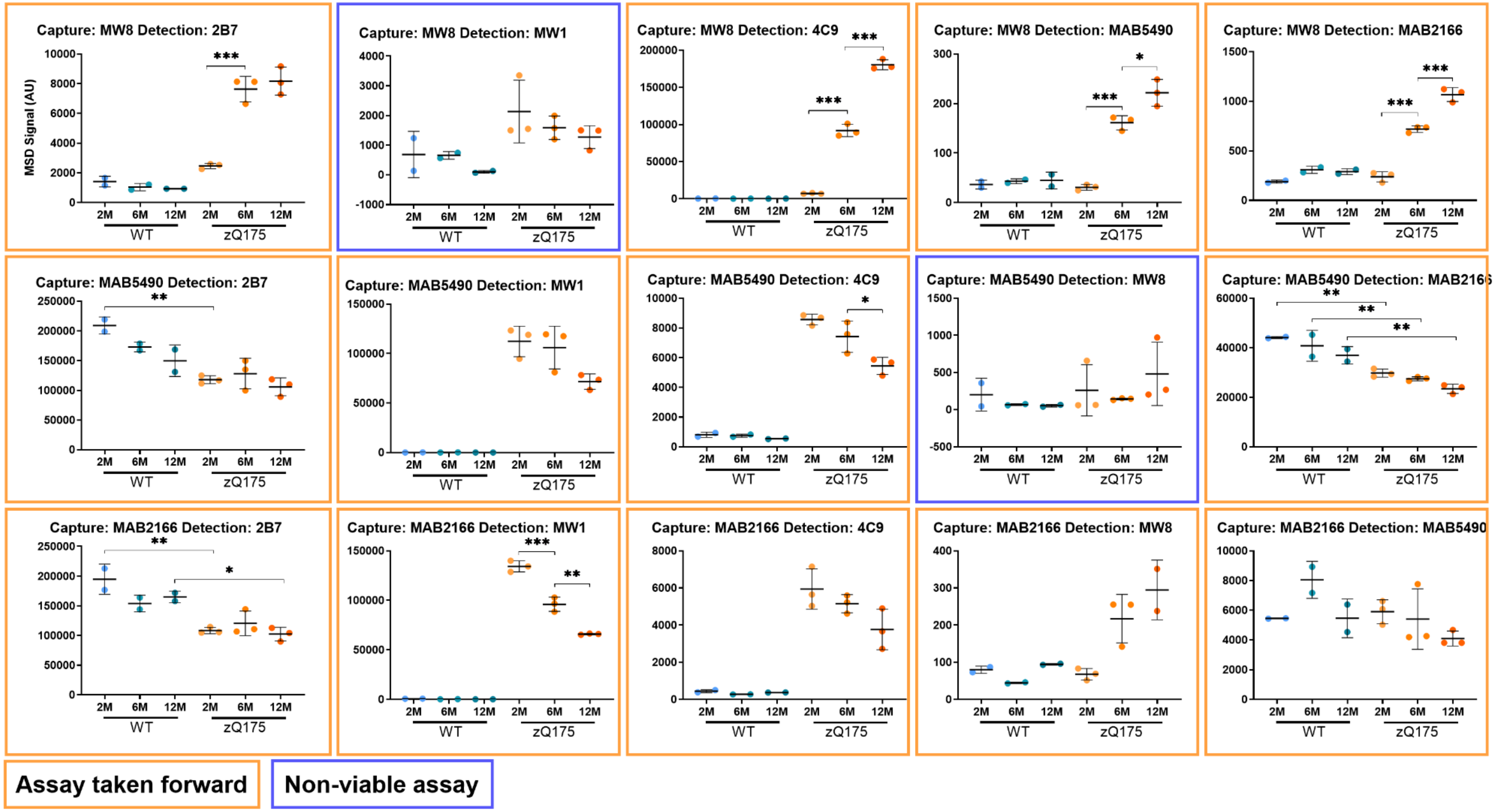

The MW8-MW1 and MAB5490-MW8 antibody pairings were considered non-viable due to the very low signal to noise ratio.

Supplementary Figure 2. Optimisation of antibody concentrations for use in the 'total soluble mutant HTT' assays.

Optimisation of antibody concentrations for assays that detect 'total soluble mutant HTT' were performed on cortical lysates from 2 month old zQ175 mice that have the greatest concentration of soluble mutant HTT in the age range under investigation. **(A)** For HTRF, the donor antibody concentration was kept constant at 1 ng / well, and the acceptor antibody concentration was titrated from 1 ng / well to 40 ng / well. The maximum concentration prior to saturation was chosen as optimal (arrow). The zQ175 heterozygous lysates were diluted with age-matched wild type lysate. **(B)** For AlphaLISA, the biotinylated donor antibody was titrated from 0.1 – 100 nM per well and the acceptor antibody concentration remained constant at 20 µg / mL. The maximum concentration prior to the hook was selected as optimal (arrow). **(C)** For MSD, the capture antibody was 2 mg/mL ± 15% and the detection antibody was titrated from 0.15 µg / mL – 4.5 µg / mL. The maximum concentration prior to saturation was selected as optimal (arrow). The change in fluorescent signal is denoted as ΔF%. WT = wild-type.

Supplementary Figure 3. Titration of mutant HTT in zQ175 lysates for optimisation of the 'total soluble mutant HTT' assays.

Optimisation of antibody concentrations for assays that detect 'total soluble mutant HTT' were performed on cortical lysates from 2 month old zQ175 mice that have the greatest concentration of soluble mutant HTT in the age range under investigation. The antibody concentrations were as determined in Supplementary Fig. 2 and are indicated with an arrow for the HTRF titration matrices. Two fold serial dilutions of cortical lysates from zQ175 heterozygotes were performed by diluting with age-matched wild type lysate and were (**A)** 1.25 – 10 µL for HTRF, **(B)** 0.08 - 10 µL for AlphaLISA and **(C)** 1.25 - 20 µL for MSD. For logistical purposes, the assays were subsequently run with 10 µL of cortical or striatal lysate. These graphs indicated whether, at that lysate concentration, a decrease in soluble mutant HTT would fall within the linear range of the assay. The change in fluorescent signal is denoted as ΔF%. WT = wild-type.

Supplementary Figure 2

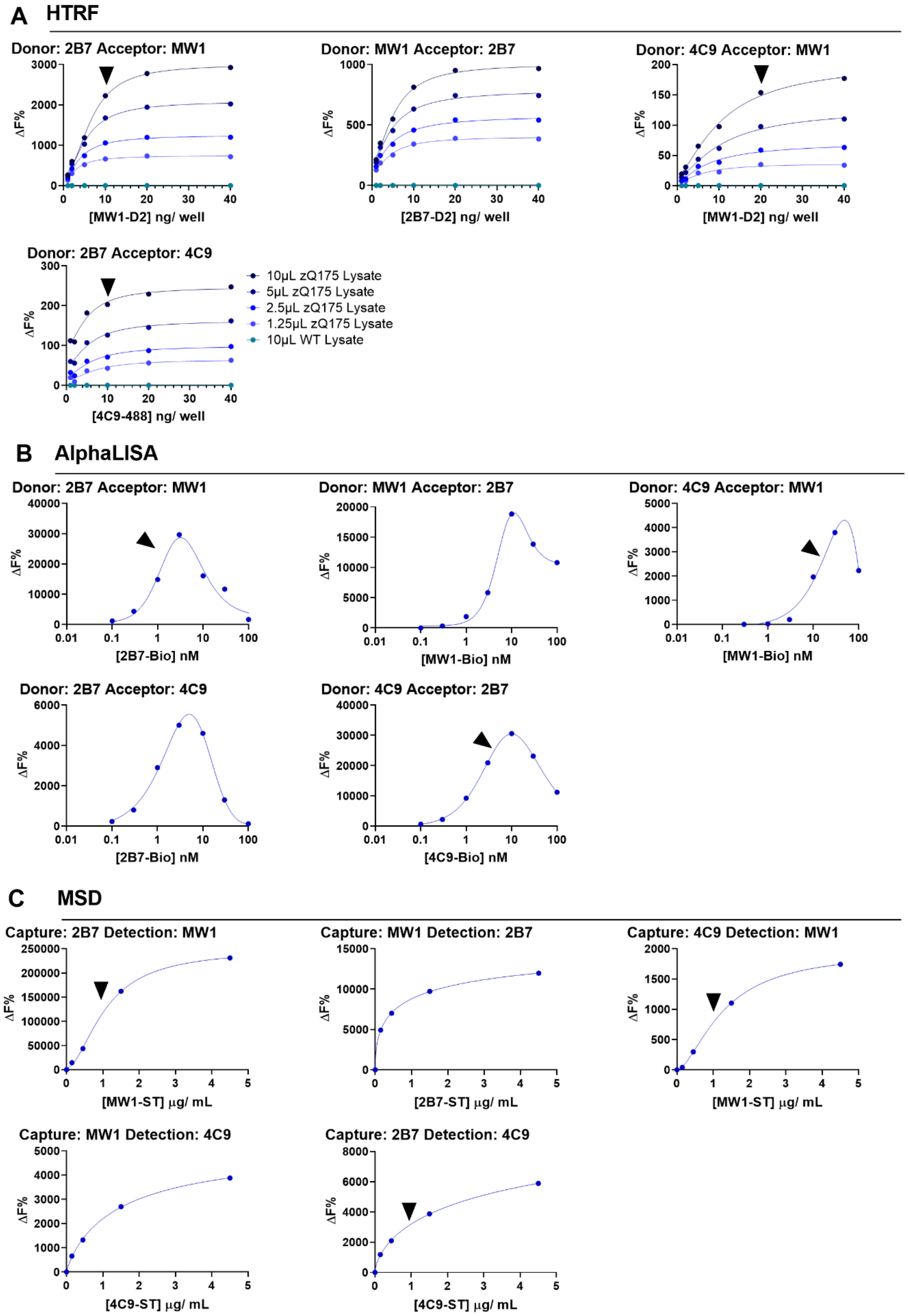

Supplementary Figure 3

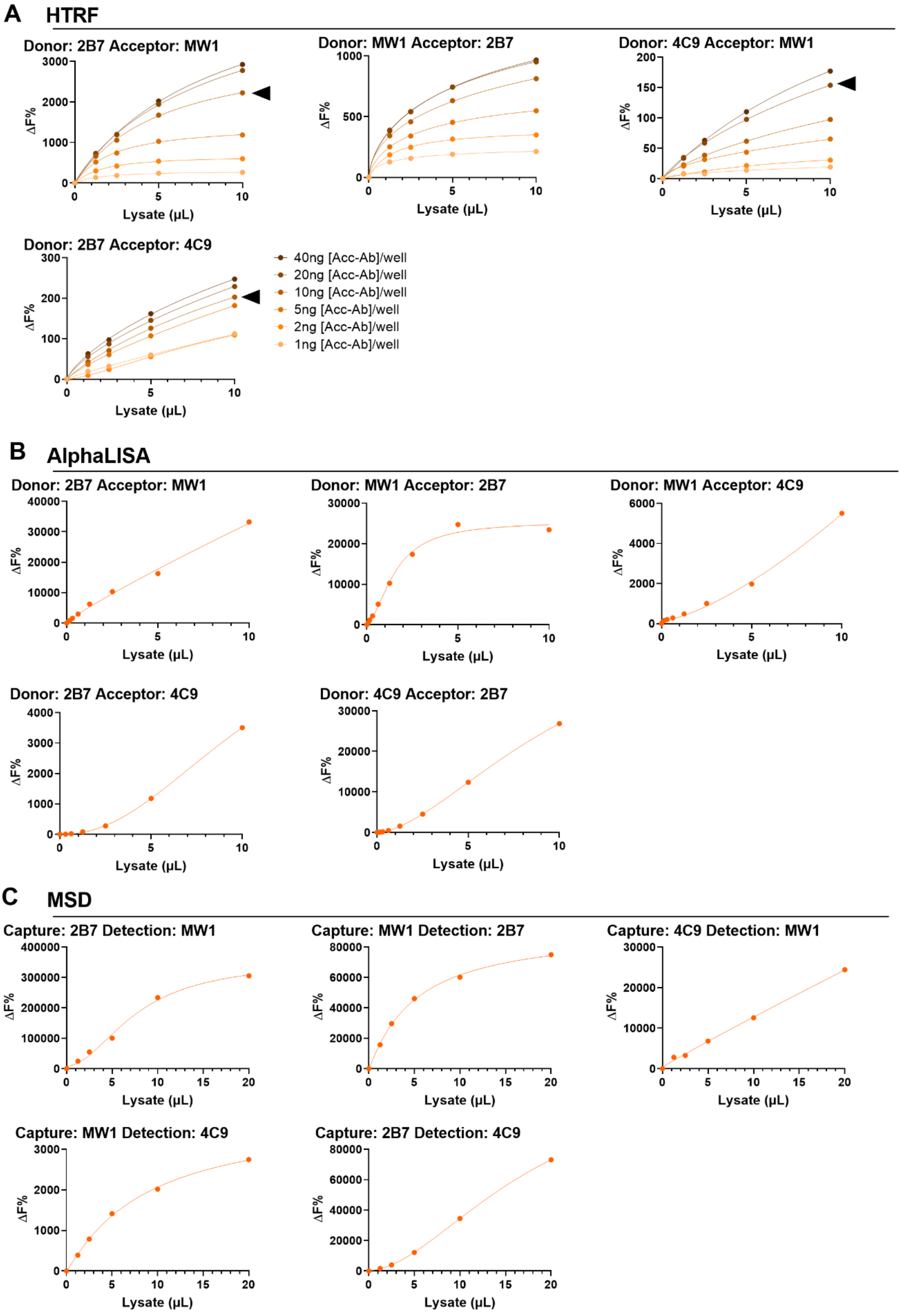

Supplementary Figure 4

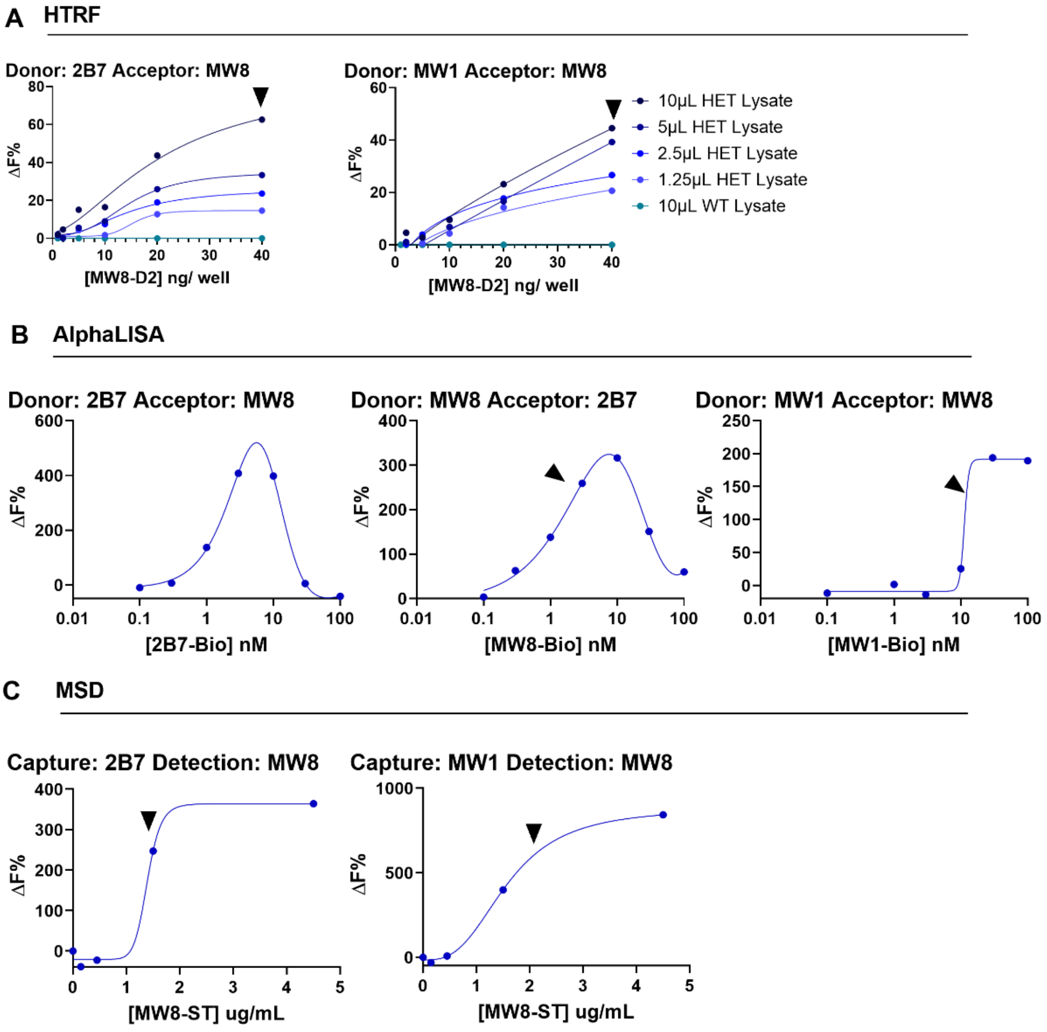

Supplementary Figure 4. Optimisation of antibody concentration for use in the 'soluble exon 1 HTT' assays.

Optimisation of antibody concentrations for assays that detect 'soluble exon 1 HTT' were performed on cortical lysates from 2 month old zQ175 mice that have the greatest concentration of soluble mutant HTT in the age range under investigation. **(A)** For HTRF, the donor antibody concentration was kept constant at 1 ng / well, and the acceptor antibody concentration was titrated from 1 ng / well to 40 ng / well. The maximum concentration prior to saturation was chosen as optimal (arrow). The zQ175 heterozygous lysates were diluted with age-matched wild type lysate. **(B)** For AlphaLISA, the biotinylated donor antibody was titrated from 0.1 – 100 nM per well and the acceptor antibody concentration remained constant at 20 µg / mL. The maximum concentration prior to the hook was selected as optimal (arrow). **(C)** For MSD, the capture antibody was 2 mg/mL ± 15% and the detection antibody was titrated from 0.15 µg / mL – 4.5 µg / mL. The maximum concentration prior to saturation was selected as optimal (arrow). The change in fluorescent signal is denoted as ΔF%. WT = wild-type.

Supplementary Figure 5

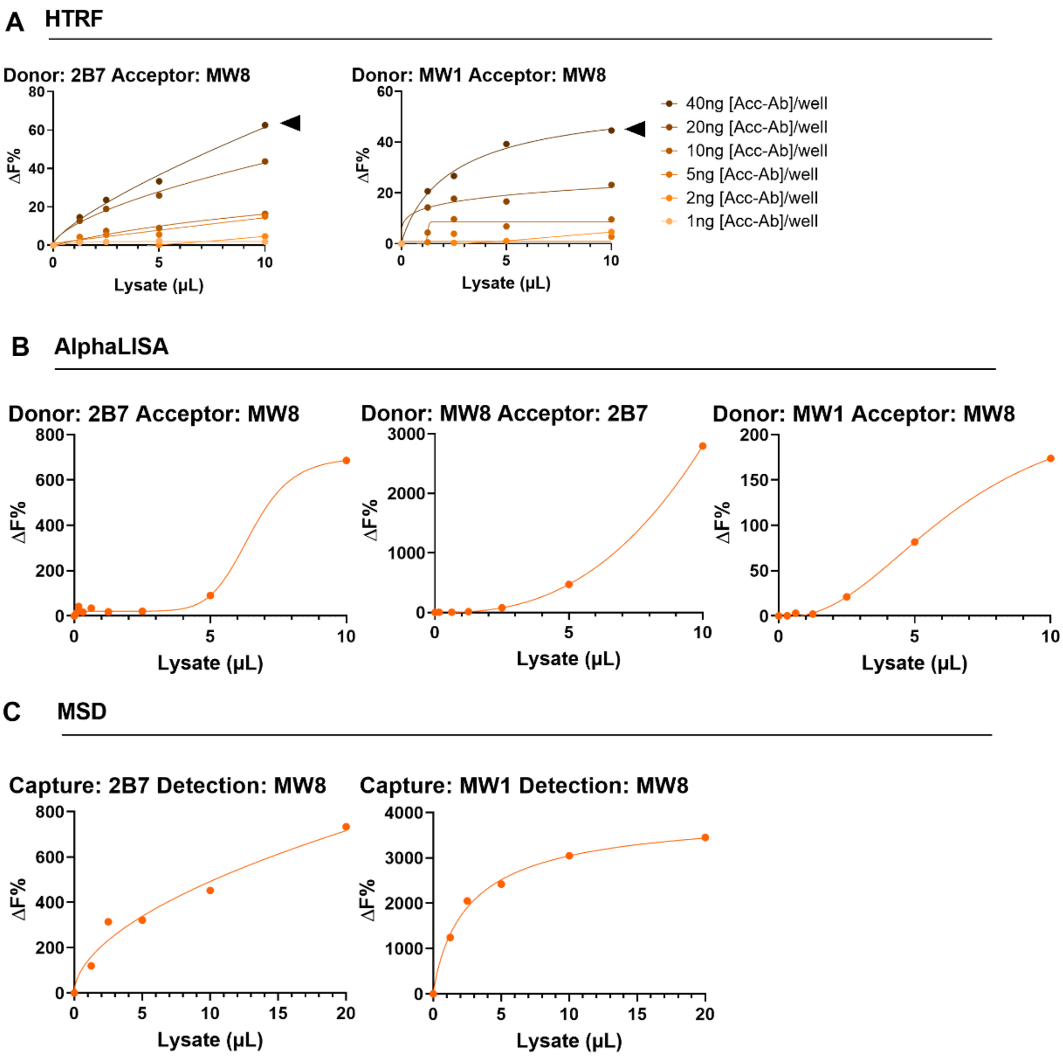

Supplementary Figure 5. Titration of mutant HTT in zQ175 lysates for optimisation of the 'soluble exon 1 HTT' assays.

Optimisation of assays that detect 'soluble exon 1 HTT' were performed on cortical lysates from 2 month old zQ175 mice that have the greatest concentration of soluble mutant HTT in the age range under investigation. The antibody concentrations were as determined in Supplementary Fig. 4 and are indicated with an arrow for the HTRF matrices. Two fold serial dilutions of cortical lysates from zQ175 heterozygotes were performed by diluting with age-matched wild type lysate and were **(A)** 1.25 – 10 μL for HTRF, **(B)** 0.08 - 10 μL for AlphaLISA and **(C)** 1.25 - 20 μL for MSD. For logistical purposes, the assays were subsequently run with 10 μL of cortical or striatal lysate. These graphs indicated whether, at that lysate concentration, a decrease in soluble mutant HTT would fall within the linear range of the assay. The change in fluorescent signal is denoted as ΔF%. WT = wild-type.

Supplementary Figure 6

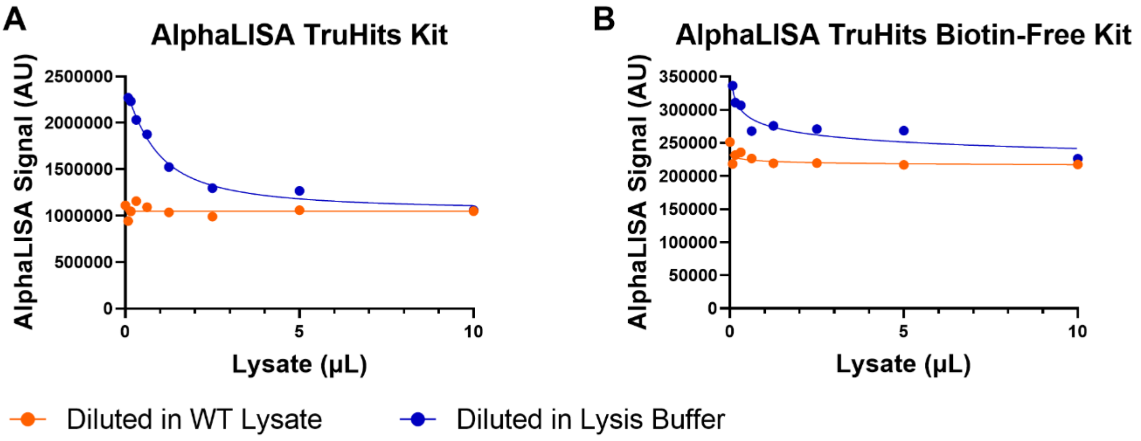

Supplementary Figure 6. Investigation of the effect of endogenous free-biotin on the performance of the AlphaLISA assays. To determine whether endogenous free-biotin might have artificially reduced the true AlphaLISA signal, cortical lysate from 12 month old zQ175 mice was subjected to a seven point two fold serial dilution from 10 – 0.16 µL with either age-matched wild type lysate, or lysis buffer, and tested in the **(A)** AlphaLISA TruHits kit and **(B)** AlphaLISA TruHits Biotin-Free kits. **(A)** With the TruHits kit, dilution with wild type lysate did not alter the AlphaLISA signal, whereas dilution with lysis buffer resulted in a recovery of the signal, indicating that free-biotin was present in the lysate. **(B)** With the TruHits Biotin-Free kit, dilution with the wild type lysate gave a lower AlphaLISA signal than with the TruHits kit. The effect of dilution with lysis buffer was less pronounced and was linked to interference related to the sample, and not to free-biotin within the lysate. WT = wild-type.

Supplementary Figure 7

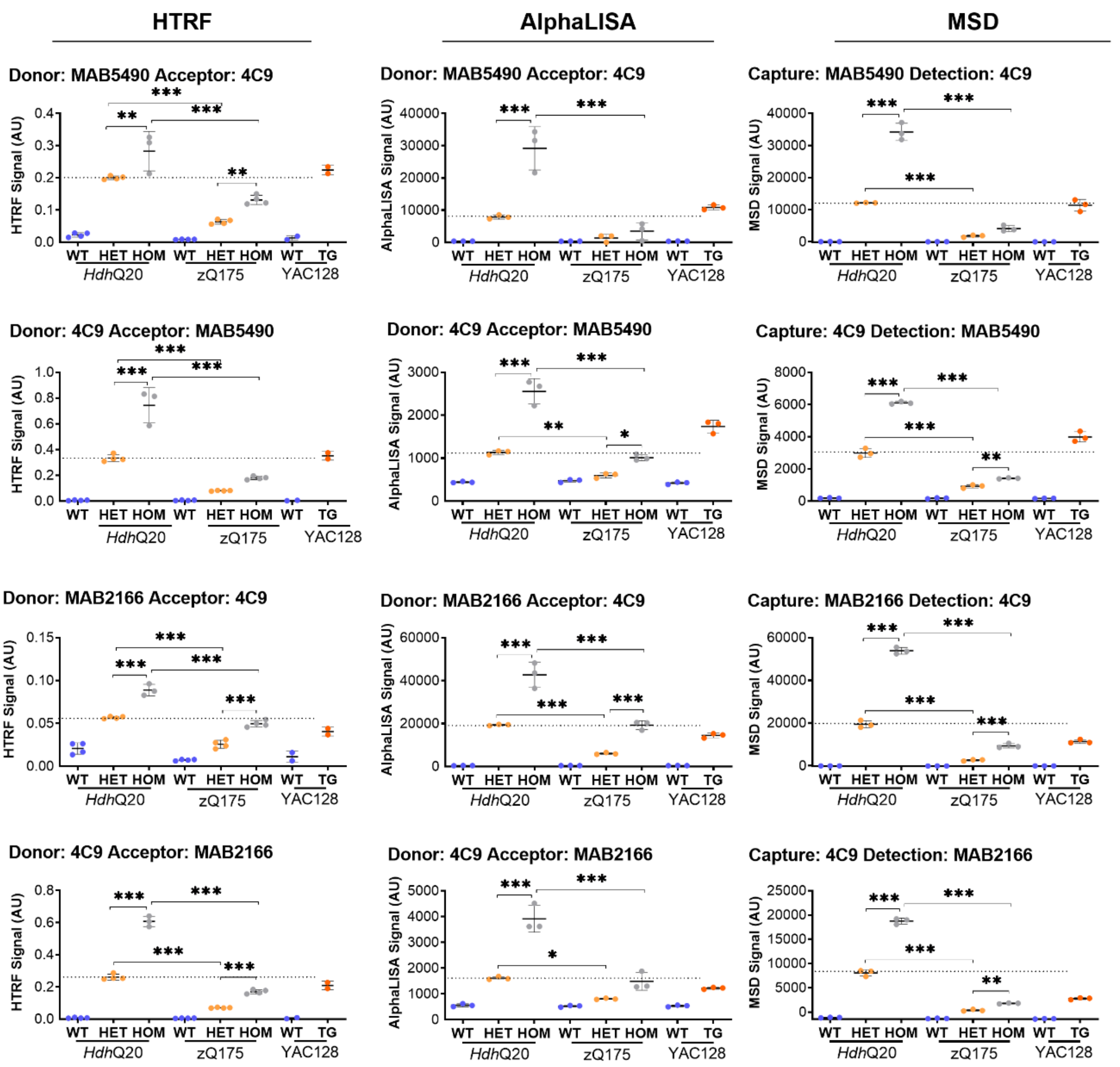

Supplementary Figure 7. Investigation of the effect of polyQ length on antibody combinations that include 4C9 for the detection of 'soluble mutant HTT' (excluding exon 1 HTT).

Antibody pairings of 4C9 with MAB5490 and 4C9 with MAB2166 in both orientations were tested by HTRF, AlphaLISA and MSD using cortical lysates from heterozygous and homozygous *Hdh*Q20, heterozygous and homozygous zQ175 (n = 3 - 4 / genotype) and YAC128 (n = 2 -3 / genotype) mice together with their respective wild type littermates at 2 months of age (n = 2 - 4 / genotype). The length of the polyQ repeat in zQ175 mice dramatically interfered with the signal in all assays on all three platforms. Statistical analysis was two-way ANOVA with Bonferroni *post hoc* correction, mean ± SEM. *$p \leq 0.5$, ** $p \leq 0.01$, ***$p \leq 0.001$. The test statistic, degrees of freedom and p values for the ANOVA are provided in Supplementary Table 13. WT = wild-type (blue), HET = heterozygote (orange), HOM = homozygote (grey). Dotted line = signal in *Hdh*Q20 heterozygous lysates.

Supplementary Figure 8. Optimisation of antibody concentrations for use in the 'soluble mutant HTT' (excluding exon 1 HTT) assays.

Optimisation of antibody concentrations for assays that detect 'soluble mutant HTT' (excluding exon 1 HTT) were performed on cortical lysates from 2 month old zQ175 mice that have the greatest concentration of soluble mutant HTT in the age range under investigation. **(A)** For HTRF, the donor antibody concentration was kept constant at 1 ng / well, and the acceptor antibody concentration was titrated from 1 ng / well to 40 ng / well. The maximum concentration prior to saturation was chosen as optimal (arrow). The zQ175 heterozygous lysates were diluted with age-matched wild type lysate. **(B)** For AlphaLISA, the biotinylated donor antibody was titrated from 0.1 – 100 nM per well and the acceptor antibody concentration remained constant at 20 µg / mL. The maximum concentration prior to the hook was selected as optimal (arrow). **(C)** For MSD, the capture antibody was 2 mg/mL ± 15% and the detection antibody was titrated from 0.15 µg / mL – 4.5 µg / mL. The maximum concentration prior to saturation was selected as optimal (arrow). The change in fluorescent signal is denoted as ΔF%. WT = wild-type.

Supplementary Figure 9. Titration of mutant HTT in zQ175 lysates for optimisation of the 'soluble mutant HTT' (excluding exon 1 HTT) assays.

Optimisation of assays that detect 'soluble mutant HTT' (excluding exon 1 HTT) were performed on cortical lysates from 2 month old zQ175 mice that have the greatest concentration of soluble mutant HTT in the age range under investigation. The antibody concentrations were as determined in Supplementary Fig. 8 and are indicated with an arrow for the HTRF matrices. Two fold serial dilutions of cortical lysates from zQ175 heterozygotes were performed by diluting with age-matched wild type lysate and were (**A)** 1.25 – 10 µL for HTRF, **(B)** 0.08 - 10 µL for AlphaLISA and **(C)** 1.25 - 20 µL for MSD. For logistical purposes, the assays were subsequently run with 10 µL of cortical or striatal lysate. These graphs indicated whether, at that lysate concentration, a decrease in soluble mutant HTT would fall within the linear range of the assay. The change in fluorescent signal is denoted as ΔF%. WT = wild-type.

Supplementary Figure 8

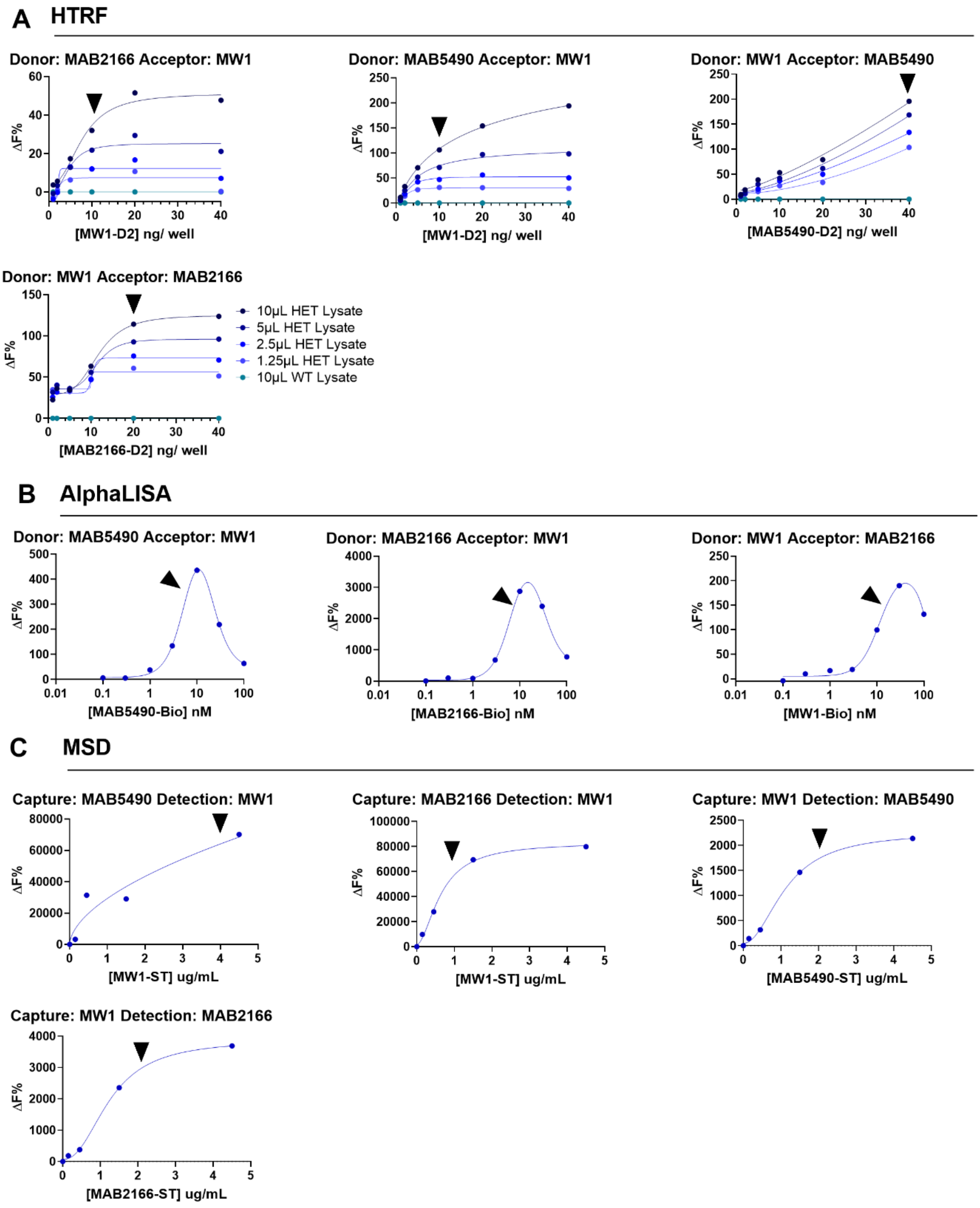

Supplementary Figure 9

A HTRF

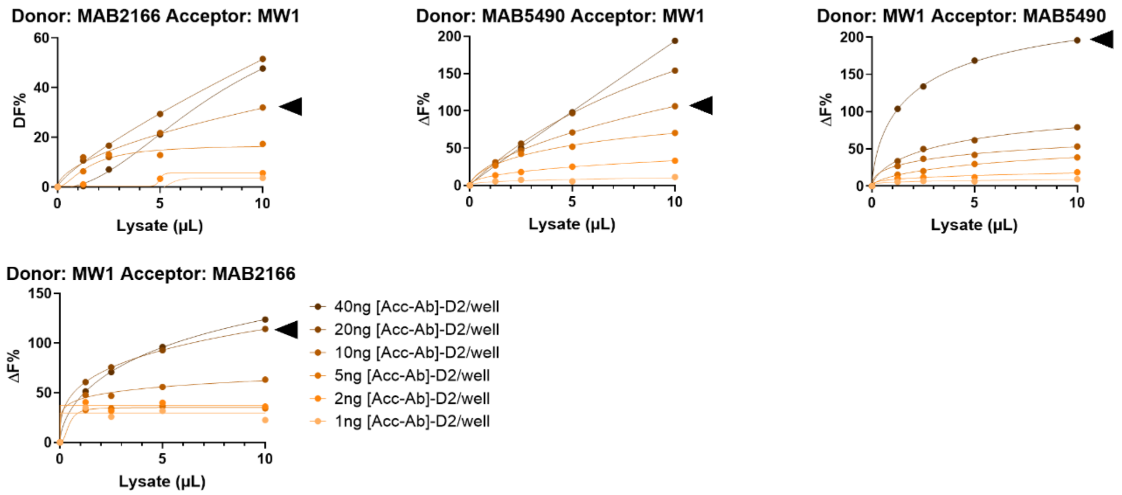

B AlphaLISA

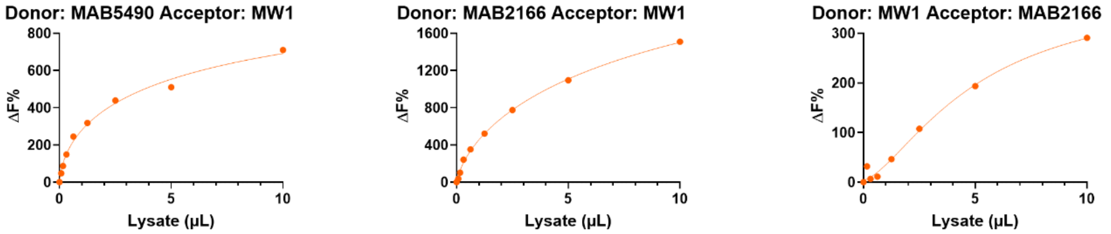

C MSD

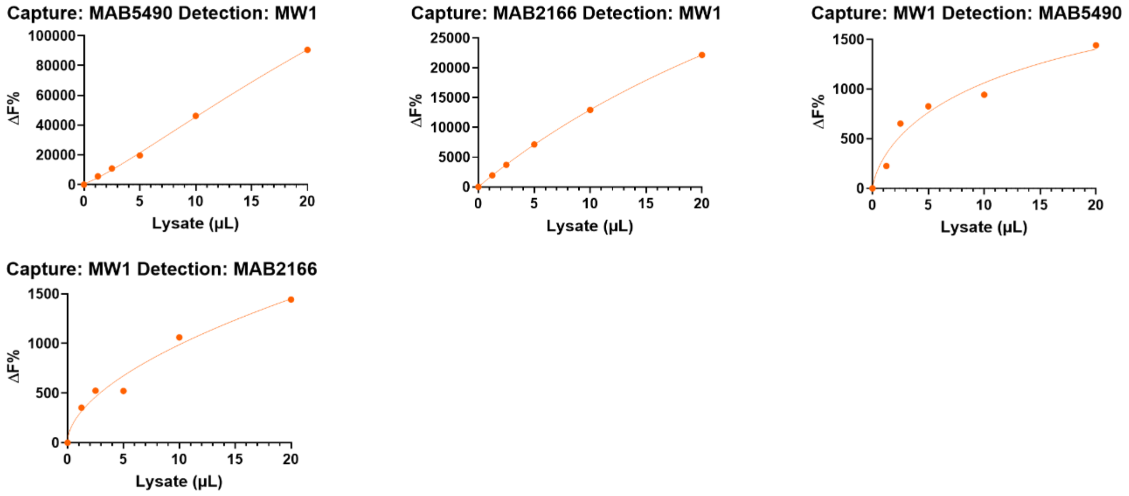

Supplementary Figure 10

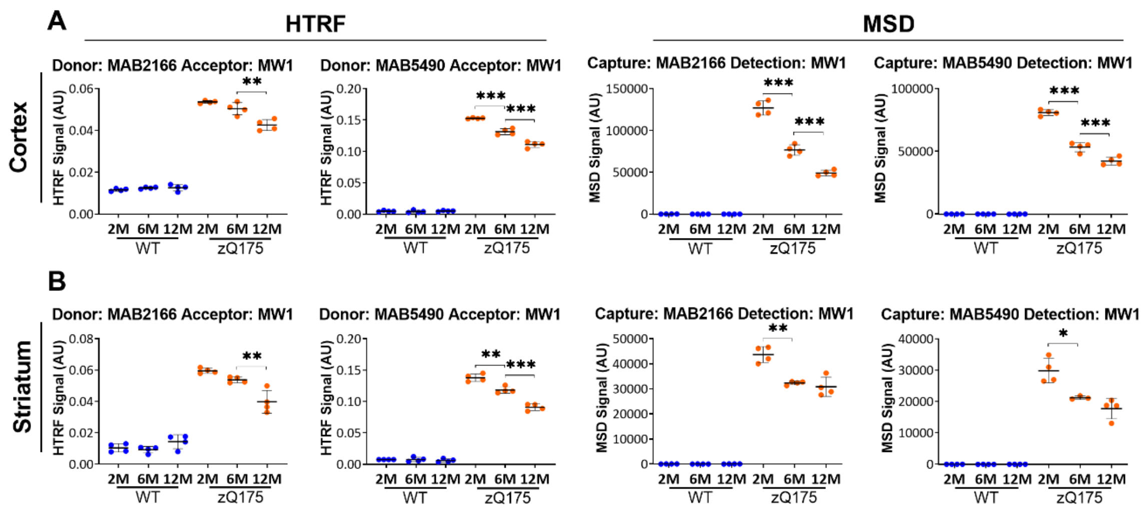

Supplementary Fig. 10. Assessment of the 'soluble mutant HTT' (excluding exon 1 HTT) assays on the HTRF and MSD platforms in cortical and striatal lysates from zQ175 mice at 2, 6 and 12 months of age.

Antibody pairings of MAB2166 or MAB5490 with MW1 were tested on the HTRF and MSD platforms. MAB5490 or MAB2166 were the donor or capture antibody and MW1 was the acceptor or detector in **(A)** cortical and **(B)** striatal lysates from zQ175 and wild type mice at 2, 6, and 12 months of age (n = 4 / genotype). In these assays, the level of 'soluble mutant HTT' (excluding exon 1 HTT) appeared to decrease with disease progression over this age range. This was in contrast to the HTRF and MSD assays in which MW1 was the donor or capture antibody and MAB5490 or MAB2166 were the acceptor or detector antibody for which the level of soluble mutant HTT remained unchanged (Fig. 6). This was also the case for all AlphaLISA assays using combinations of these antibodies (Fig. 6). These inconsistencies could not be accounted for by differences in the lysate titration curves (Supplementary Fig. 9), which, although they were not completely linear, were relatively comparable. Our interpretation of this discrepancy is that the use of MW1 as the donor or capture antibody binds soluble mutant HTT, and then MAB5490 or MAB2166 as the acceptor or detector, identifies the proportion of the soluble mutant HTT comprising HTT fragments that are longer than exon 1 HTT. In contrast, if MAB5490 or MAB2166 are used as the donor or capture antibodies, they bind both soluble and aggregated HTT. Given that the level of aggregated HTT increases with disease progression, and that MW1 does not bind to aggregated HTT, the proportion of soluble HTT detected by MW1, when it is used as the acceptor or detector antibody in this context, consequently decreases. Statistical analysis was one-way ANOVA with Bonferroni *post hoc* correction, mean ± SEM. **p* ≤ 0.5, ** *p* ≤ 0.01, ****p* ≤ 0.001. The test statistic, degrees of freedom and *p* values for the ANOVA are provided in Supplementary Table 8. WT = wild-type (blue), heterozygous zQ175 mice (orange).

Supplementary Figure 11. Optimisation of antibody concentrations for use in the 'HTT aggregation' assays.

Optimisation of antibody concentrations for assays that detect 'aggregated HTT' were performed on cortical lysates from 12 month old zQ175 mice that have the greatest concentration of aggregated HTT in the age range under investigation. **(A)** For HTRF, the donor antibody concentration was kept constant at 1 ng / well, and the acceptor antibody concentration was titrated from 1 ng / well to 40 ng / well. The maximum concentration prior to saturation was chosen as optimal (arrow). The zQ175 heterozygous lysates were diluted with age-matched wild type lysate. **(B)** For AlphaLISA, the biotinylated donor antibody was titrated from 0.1 – 100 nM per well and the acceptor antibody concentration remained constant at 20 µg / mL. The maximum concentration prior to the hook was selected as optimal (arrow). **(C)** For MSD, the capture antibody was 2 mg/mL ± 15% and the detection antibody was titrated from 0.15 µg / mL – 4.5 µg / mL. The maximum concentration prior to saturation was chosen as optimal (arrow). The change in fluorescent signal is denoted as ΔF%. WT = wild-type.

Supplementary Figure 12. Titration of mutant HTT in zQ175 lysates for optimisation of the 'HTT aggregation' assays.

Optimisation of assays that detect 'aggregated HTT' were performed on cortical lysates from 12 month old zQ175 mice that have the greatest concentration of aggregated HTT in the age range under investigation. The antibody concentrations were as determined in Supplementary Fig. 11 and are indicated with an arrow for the HTRF matrices. Two fold serial dilutions of cortical lysates from zQ175 heterozygotes were performed by diluting with age-matched wild type lysate and were (**A)** 1.25 – 10 µL for HTRF, **(B)** 0.08 - 10 µL for AlphaLISA and **(C)** 1.25 - 20 µL for MSD. For logistical purposes, the assays were subsequently run with 10 µL of cortical or striatal lysate. These graphs indicated whether, at that lysate concentration, a decrease in aggregated HTT would fall within the linear range of the assay. The change in fluorescent signal is denoted as ΔF%. WT = wild-type.

Supplementary Figure 11

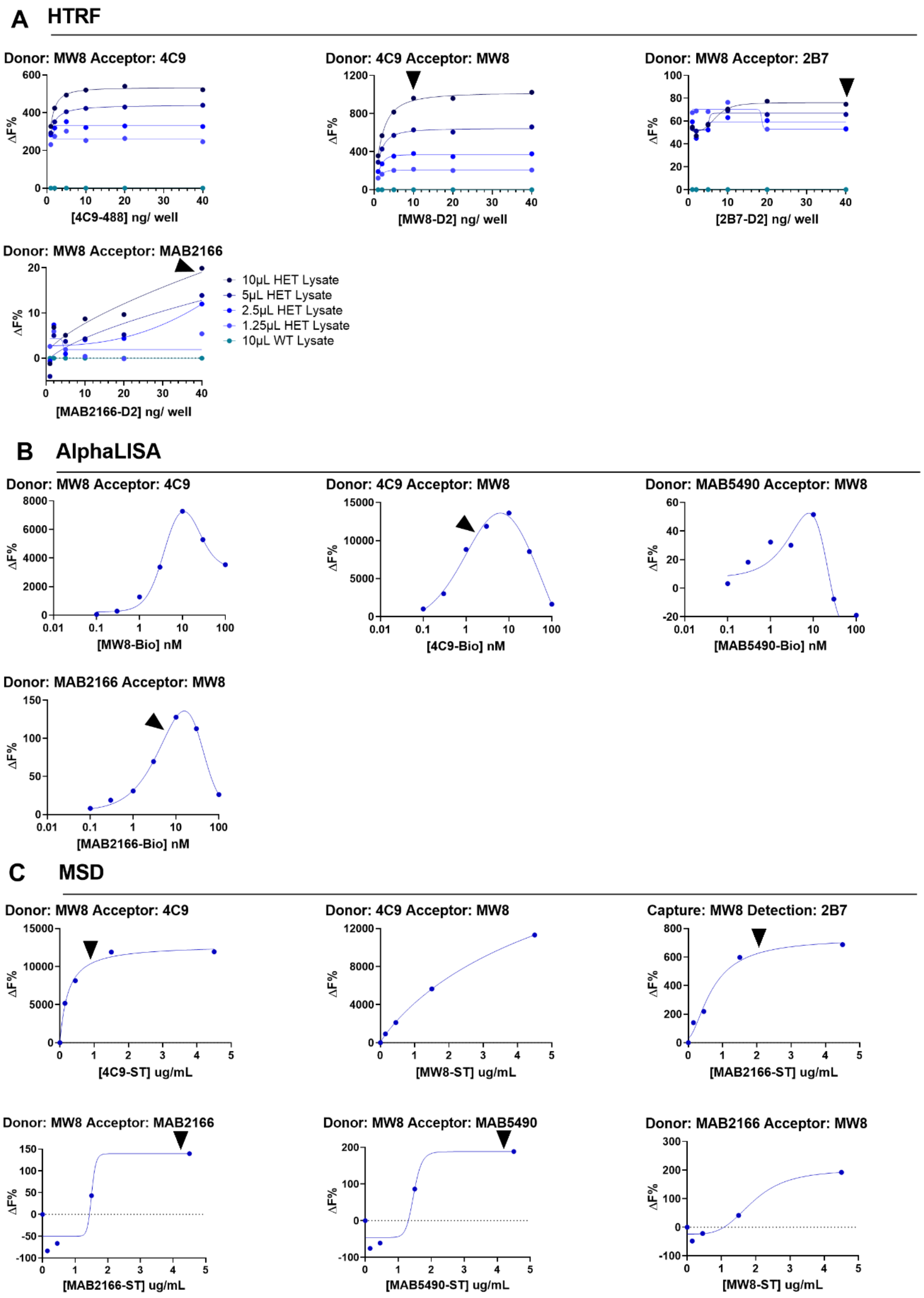

Supplementary Figure 12

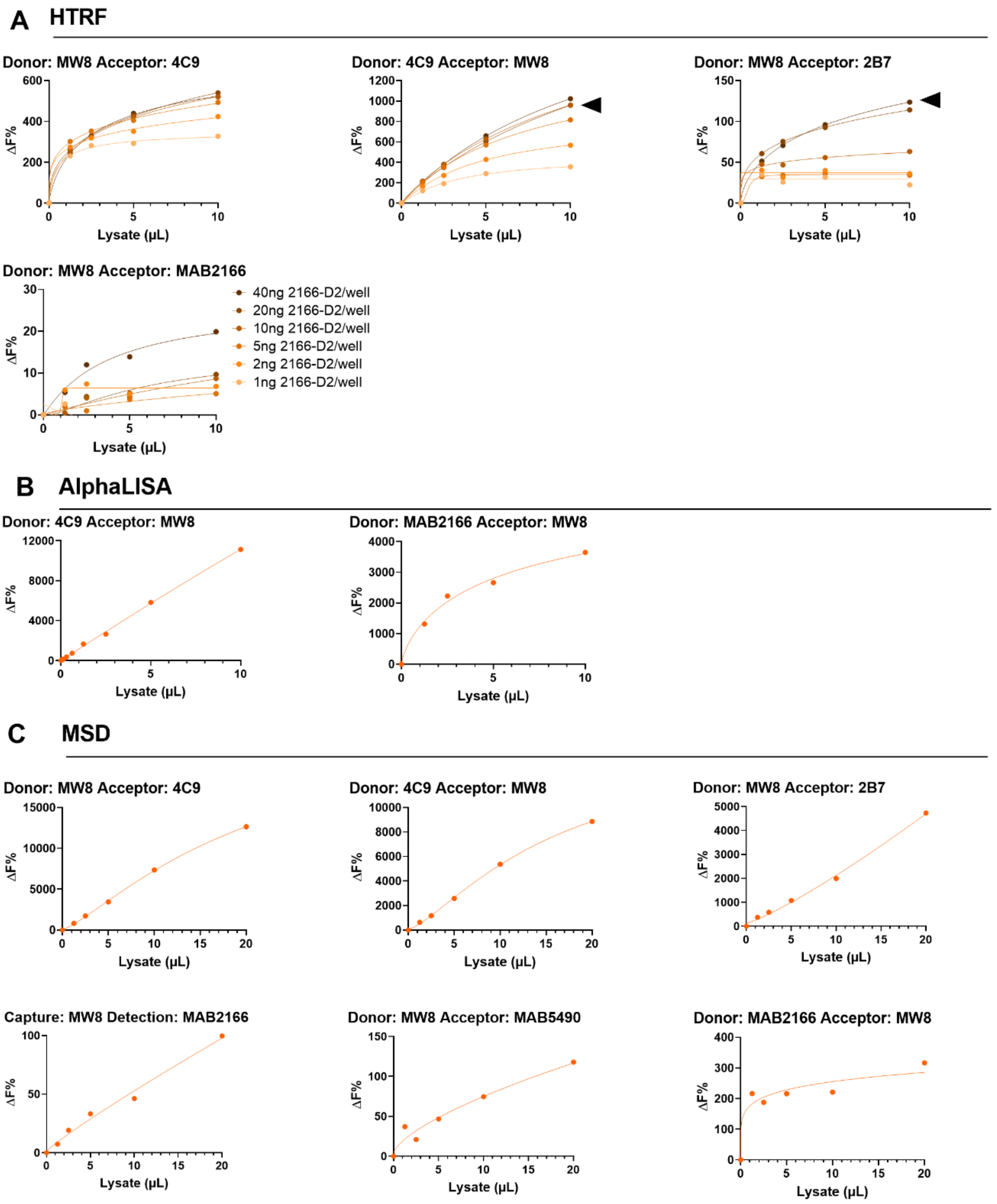

Supplementary Figure 13. Investigation of the effect of polyQ length on assays to detect 'total soluble full-length HTT' (mutant and wild type).

Antibody pairings including 2B7 with MAB4590, 2B7 with MAB2166 and MAB5490 with MAB2166, in both orientation were tested by HTRF, AlphaLISA and MSD using cortical lysates from heterozygous and homozygous *Hdh*Q20, heterozygous and homozygous zQ175 (n = 3 – 4 / genotype) and YAC128 (n = 2 – 3 / genotype) mice together with their respective wild type littermates at 2 months of age. If the length of the polyQ repeat had no effect on assay performance, the signal in YAC128 lysate should be increased (two copies endogenous mouse *Htt* and one copy of mutant human *HTT*), and the signals for all *Hdh*Q20 and zQ175 genotypes should be similar to wild type. The only assays taken forward for optimisation were MAB5490 and MAB2166 antibody pairings in both orientations on the HTRF and AlphaLISA platforms and MAB5490-MAB2166 for MSD, although most of these did not fulfil the above criteria. Statistical analysis was two-way ANOVA with Bonferroni *post hoc* correction, mean ± SEM. *$p \leq 0.5$, ** $p \leq 0.01$, ***$p \leq 0.001$. The test statistic, degrees of freedom and *p* values for the ANOVA are provided in Supplementary Table 14. WT = wild type (blue), HET = heterozygote (orange), HOM = homozygote (grey). Dotted line = signal in *Hdh*Q20 wild-type.

Supplementary Figure 14. Investigation of the effect of polyQ length on assays to detect 'total soluble full-length HTT' (mutant and wild type).

Antibody pairings of D7F7 with 2B7, D7F7 with MAB5490 and D7F7 with MAB2166 in both orientations were tested by HTRF and MSD using cortical lysates from heterozygous and homozygous *Hdh*Q20, heterozygous and homozygous zQ175 and YAC128 mice together with their respective wild type littermates at 2 months of age (n = 3 - 4 / genotype). If the length of the polyQ repeat had no effect on assay performance, the signal in YAC128 lysate should be increased (two copies endogenous mouse *Htt* and one copy of mutant human *HTT*) and the signals for all *Hdh*Q20 and zQ175 genotypes should be similar to wild type. None of the assays fulfilled these criteria. The only assays taken forward for optimisation were the D7F7 antibody pairings with MAB5490 and MAB2166 on the HTRF platform and D7F7-MAB5490 for MSD. Statistical analysis was two-way ANOVA with Bonferroni *post hoc* correction, mean ± SEM. *$p \leq 0.5$, ** $p \leq 0.01$, ***$p \leq 0.001$. The test statistic, degrees of freedom and *p* values for the ANOVA are provided in Supplementary Table 15. WT = wild type (blue), HET = heterozygote (orange), HOM = homozygote (grey). Dotted line = signal in *Hdh*Q20 wild-type.

Supplementary Figure 13

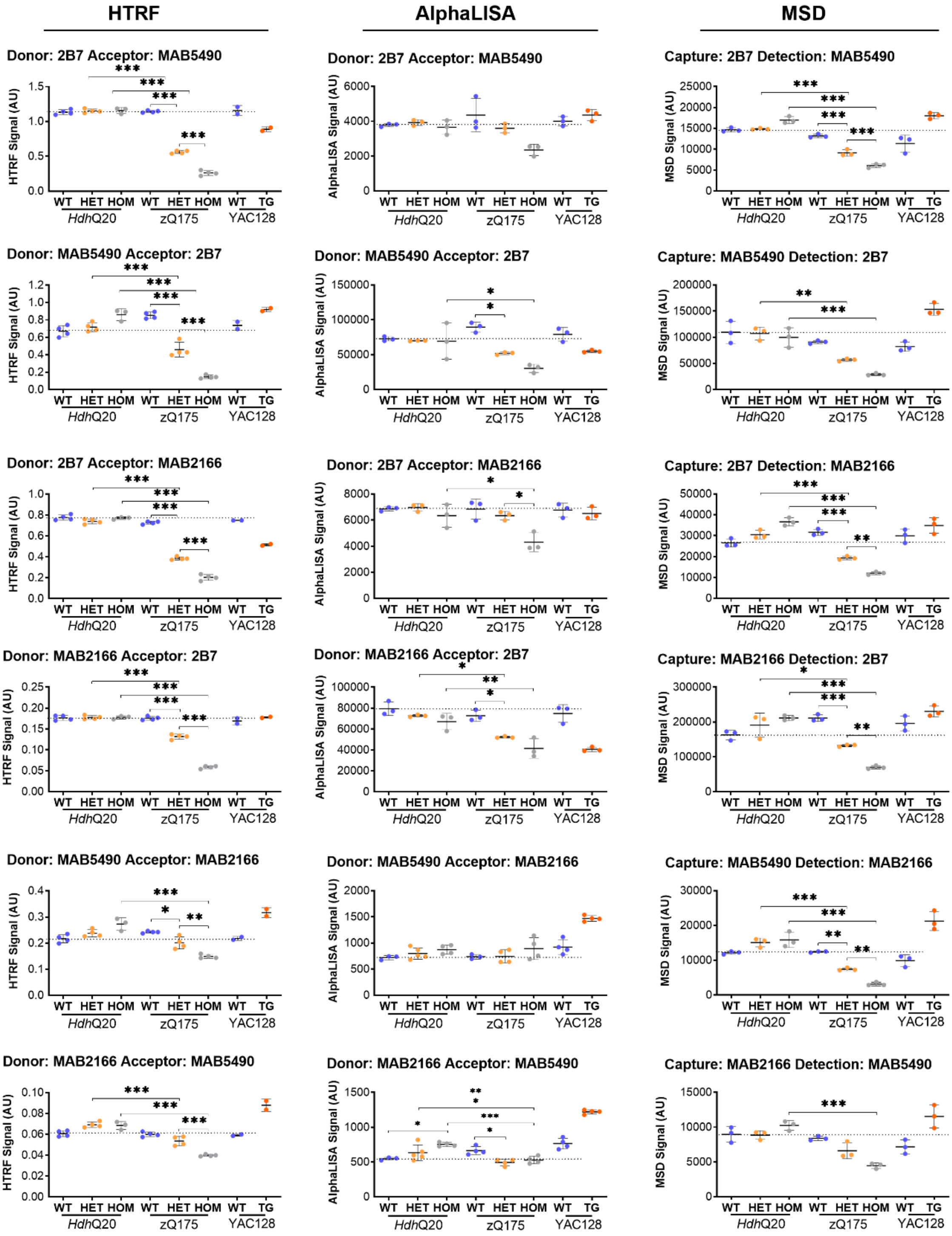

Supplementary Figure 14

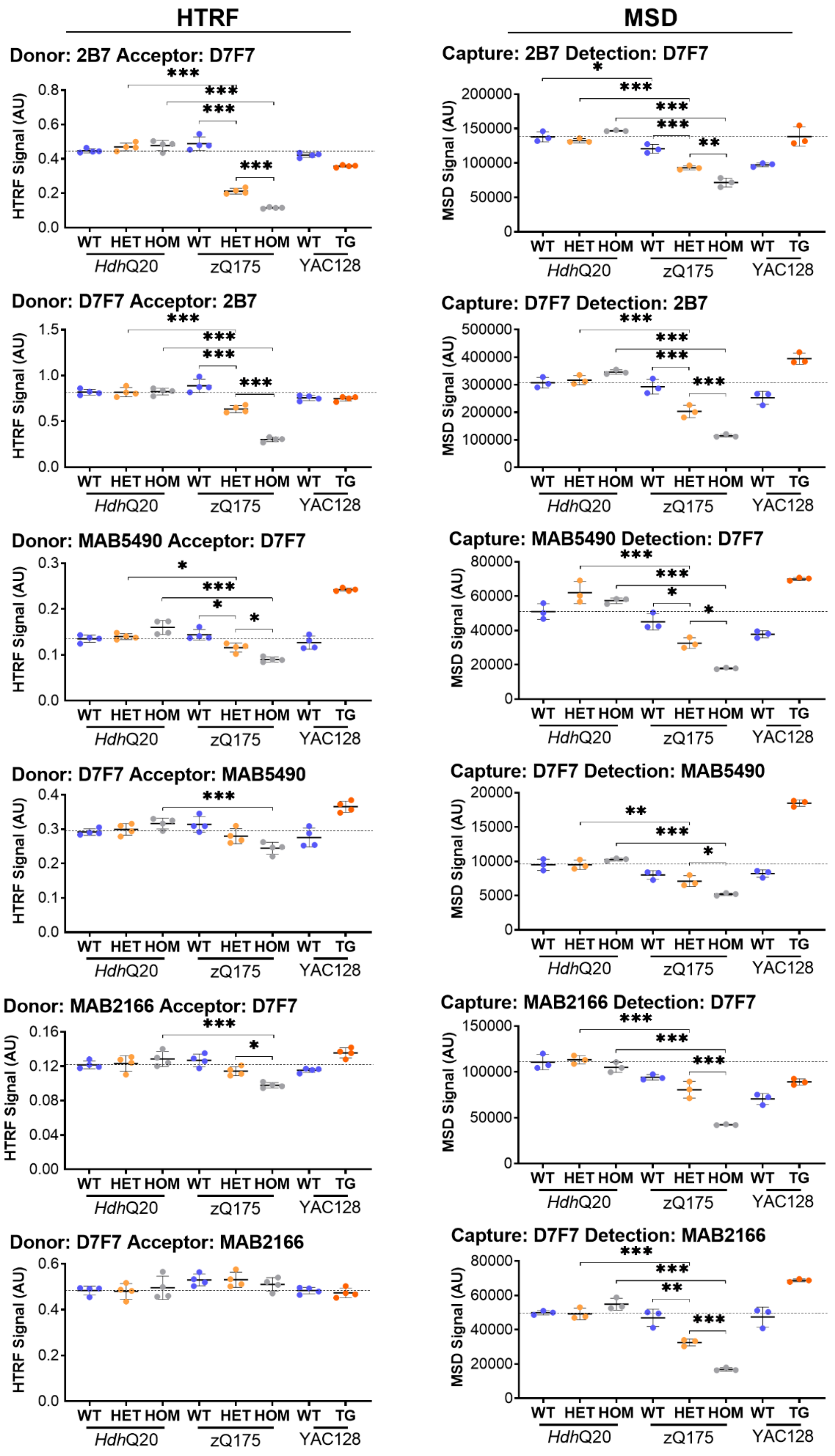

Supplementary Figure 15

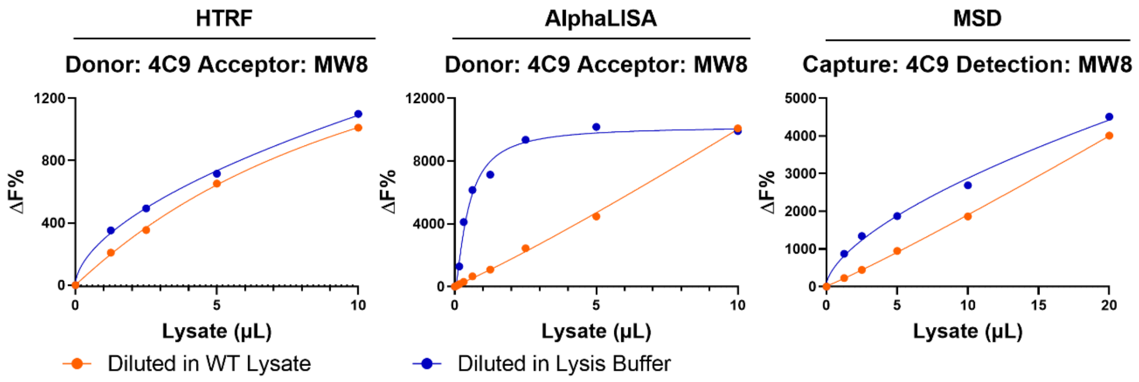

Supplementary Figure 15. Comparison of the performance of the 4C9-MW8 aggregation assay for which serial dilutions of zQ175 lysates have been performed in wild type lysates or lysis buffer.

Two fold serial dilutions of cortical lysates from 12 month old zQ175 heterozygous mice were performed by diluting with either age-matched wild type lysate or with lysis buffer. The dilution series were as used previously: 1.25 – 10 µL for HTRF, 0.08 - 10 µL for AlphaLISA and 1.25 - 20 µL for MSD. The 4C9-MW8 'HTT aggregation' assay was run with antibody concentrations as determined in Supplementary Fig. 11. The performance of the HTRF and MSD assays were relatively comparable, irrespective of whether the dilutions had been prepared with wild type lysate, or with lysis buffer. In contrast, dilution in lysis buffer cannot be used to prepare a titration curve for AlphaLISA assays as their performance is sensitive to the matrix in which the analyte is being measured. The change in fluorescent signal is denoted as ΔF%. WT = wild-type.

Supplementary Figure 16

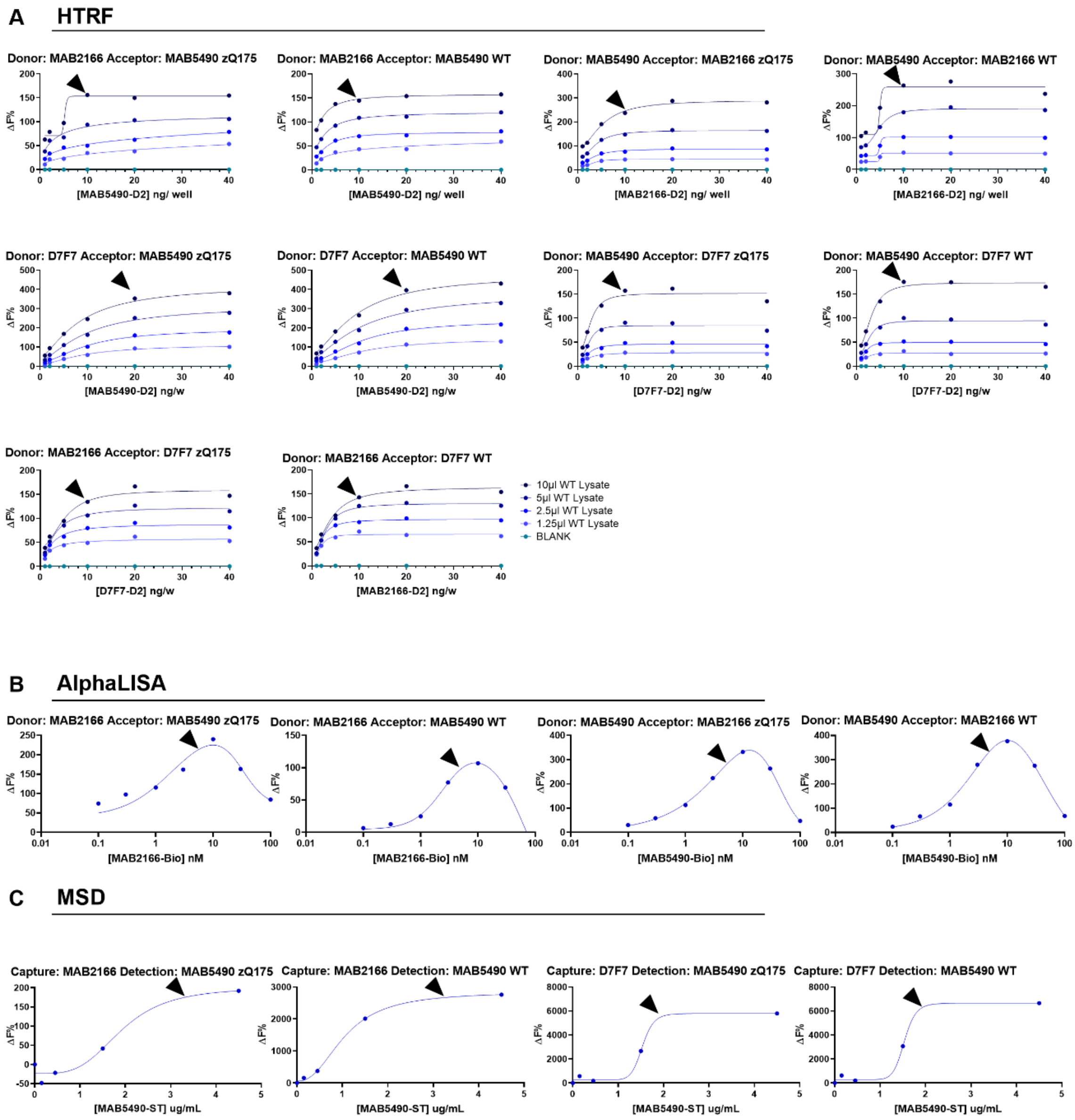

Supplementary Figure 16. Optimisation of antibody concentrations for use in the 'total soluble full-length HTT' (mutant and wild type) assays.

Optimisation of antibody concentrations for assays that detect 'total soluble full-length HTT' were performed on cortical lysates from 2 month old zQ175 and wild-type mice. **(A)** For HTRF, the donor antibody concentration was kept constant at 1 ng / well, and the acceptor antibody concentration was titrated from 1 ng / well to 40 ng / well. The maximum concentration prior to saturation was chosen as optimal (arrow). The zQ175 heterozygous lysates were diluted with lysis buffer. **(B)** For AlphaLISA, the biotinylated donor antibody was titrated from 0.1 – 100 nM per well and the acceptor antibody concentration remained constant at 20 µg / mL. The maximum concentration prior to the hook was selected as optimal (arrow). **(C)** For MSD,

the capture antibody was 2 mg/mL ± 15% and the detection antibody was titrated from 0.15 µg / mL – 4.5 µg / mL. The maximum concentration prior to saturation was selected as optimal (arrow). The change in fluorescent signal is denoted as ΔF%. WT = wild-type.

Supplementary Figure 17

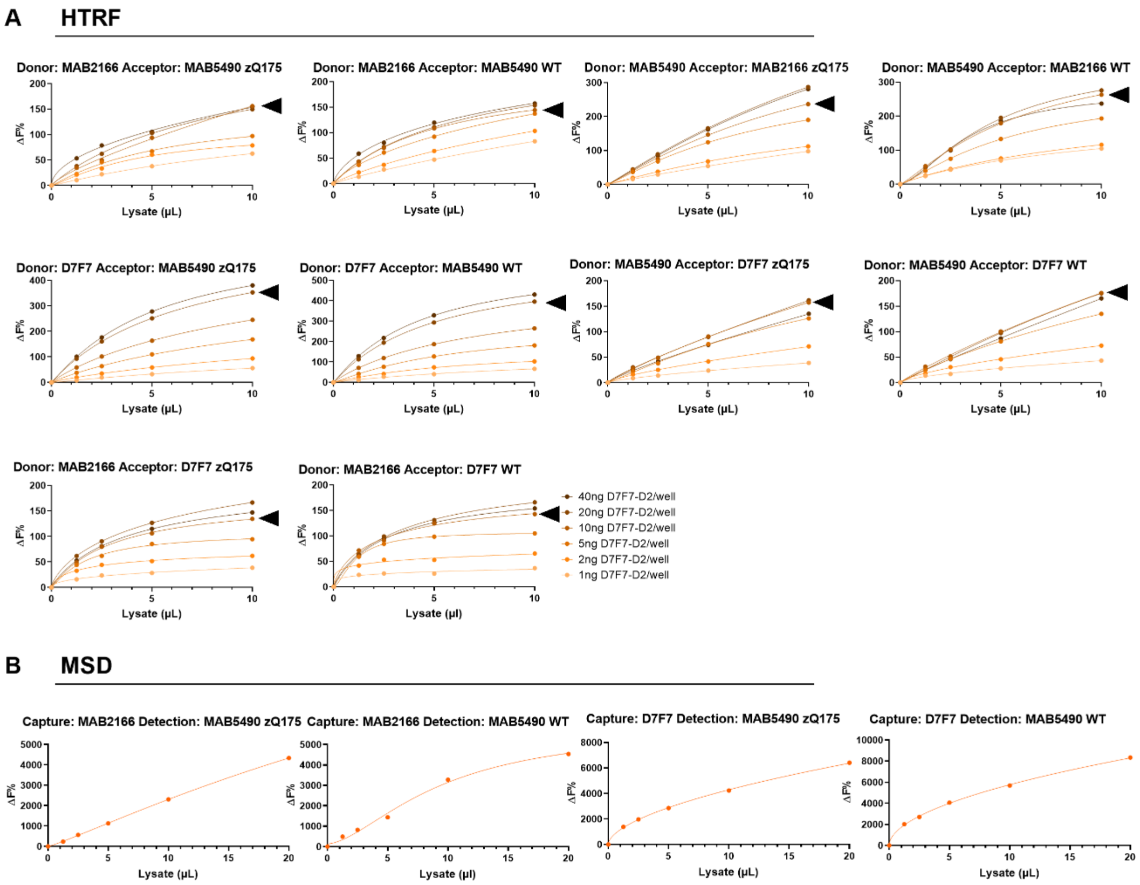

Supplementary Figure 17. Titration of zQ175 or wild type lysates with lysis buffer to optimise conditions for use in the 'total soluble full-length HTT' (mutant and wild type) assays.

Optimisation of assays that detect 'total soluble full-length HTT' were performed on cortical lysates from 2 month old zQ175 and wild-type mice. The antibody concentrations were as determined in Supplementary Fig. 16 and are indicated with an arrow for the HTRF matrices. Two fold serial dilutions of zQ175 heterozygous lysates and wild type lysates were performed by diluting with lysis buffer and were **(A)** 1.25 – 10 µL for HTRF and **(B)** 1.25 - 20 µL for MSD. For logistical purposes, the assays were subsequently run with 10 µL of cortical or striatal lysate. These graphs indicated whether, at that lysate concentration, a decrease in total soluble HTT would fall within the linear range of the assay. The change in fluorescent signal is denoted as ΔF%. WT = wild-type.

Supplementary Table 3. Two-way ANOVA for Figure 2.

	HTRF	AlphaLISA	MSD
Donor/ Capture Ab: 2B7 Acceptor/ Detection Ab: 4C9			
Genotype	F (2, 17) = 1150, P = < 0.0001	F (2, 12) = 50.55, P = < 0.0001	F (2, 12) = 944.8, P = < 0.0001
Model	F (1, 17) = 163.0, P = < 0.0001	F (1, 12) = 12.85, P = 0.0037	F (1, 12) = 2.889, P = 0.1146
Genotype x Model	F (2, 17) = 52.47, P = < 0.0001	F (2, 12) = 6.258, P = 0.0138	F (2, 12) = 11.70, P = 0.0015
Donor/ Capture Ab: 4C9 Acceptor/ Detection Ab: 2B7			
Genotype	F (2, 17) = 162.8, P = < 0.0001	F (2, 12) = 30.94, P = < 0.0001	F (2, 12) = 3692, P = < 0.0001
Model	F (1, 17) = 135.6, P = < 0.0001	F (1, 12) = 0.8154, P = 0.3843	F (1, 12) = 5743, P = < 0.0001
Genotype x Model	F (2, 17) = 46.57, P = < 0.0001	F (2, 12) = 0.2264, P = 0.8007	F (2, 12) = 2050, P = < 0.0001

Ab = antibody.

Supplementary Table 4. One-way ANOVA for Figure 3.

HTRF			
Donor Ab	Acceptor Ab	Cortex	Striatum
4C9	MW1	F (2, 9) = 643.9, P = < 0.0001	F (2, 9) = 177.9, P = < 0.0001
2B7	4C9	F (2, 9) = 64.27, P = < 0.0001	F (2, 9) = 82.53, P = < 0.0001
AlphaLISA			
Donor Ab	Acceptor Ab	Cortex	Striatum
2B7	MW1	F (2, 7) = 49.72, P = < 0.0001	F (2, 8) = 23.92, P = 0.0004
4C9	MW1	F (2, 9) = 325.3, P = < 0.0001	F (2, 8) = 116.7, P = < 0.0001
2B7	4C9	F (2, 9) = 65.31, P = <0.0001	F (2, 9) = 68.45, P = <0.0001
MSD			
Capture Ab	Detection Ab	Cortex	Striatum
2B7	MW1	F (2, 9) = 175.8, P = < 0.0001	F (2, 9) = 42.78, P = <0.0001
4C9	MW1	F (2, 9) = 168.1, P = < 0.0001	F (2, 9) = 0.1747, P = 0.8425
2B7	4C9	F (2, 9) = 388.4, P = < 0.0001	F (2, 9) = 79.36, P = <0.0001

Ab = antibody.

Supplementary Table 5. Two-way ANOVA for Figure 4 A, B.

	HTRF	AlphaLISA	MSD
	Donor Ab: 2B7<br>Acceptor Ab: MW1	Donor Ab: 2B7<br>Acceptor Ab: MW1	Capture Ab: 2B7<br>Detection Ab: MW1
Genotype	F (1, 10) = 5083<br>P = <0.0001	F (1, 10) = 163.5<br>P = <0.0001	F (1, 10) = 554.0<br>P = <0.0001
Model	F (1, 10) = 112.1<br>P = <0.0001	F (1, 10) = 30.15<br>P = 0.0003	F (1, 10) = 1657<br>P = <0.0001
Genotype x Model	F (1, 10) = 117.4<br>P = <0.0001	F (1, 10) = 30.26<br>P = 0.0003	F (1, 10) = 554.8<br>P = <0.0001
	Donor Ab: 2B7<br>Acceptor Ab: 4C9	Donor Ab: 4C9<br>Acceptor Ab: MW1	Capture Ab: 2B7<br>Detection Ab: 4C9
Genotype	F (1, 10) = 572.3<br>P = <0.0001	F (1, 10) = 87.53<br>P = <0.0001	F (1, 10) = 123.3<br>P = <0.0001
Model	F (1, 10) = 8.920<br>P = 0.0137	F (1, 10) = 17.78<br>P = 0.0018	F (1, 10) = 2137<br>P = <0.0001
Genotype x Model	F (1, 10) = 7.121<br>P = 0.0236	F (1, 10) = 18.54<br>P = 0.0015	F (1, 10) = 124.2<br>P = <0.0001
	Donor Ab: 2B7<br>Acceptor Ab: MW8	Donor Ab: MW8<br>Acceptor Ab: 2B7	Capture Ab: 2B7<br>Detection Ab: MW8
Genotype	F (1, 10) = 153.2<br>P = <0.0001	F (1, 10) = 27.26<br>P = 0.0004	F (1, 10) = 75.69<br>P = <0.0001
Model	F (1, 10) = 87.40<br>P = <0.0001	F (1, 10) = 27.24<br>P = 0.0004	F (1, 10) = 121.8<br>P = <0.0001
Genotype x Model	F (1, 10) = 92.69<br>P = <0.0001	F (1, 10) = 27.36<br>P = 0.0004	F (1, 10) = 92.68<br>P = <0.0001
	Donor Ab: MW1<br>Acceptor Ab: MW8	Donor Ab: MW1<br>Acceptor Ab: MW8	Capture Ab: MW1<br>Detection Ab: MW8
Genotype	F (1, 10) = 28.71<br>P = 0.0003	F (1, 10) = 15.30<br>P = 0.0029	F (1, 10) = 190.7<br>P = <0.0001
Model	F (1, 10) = 36.10<br>P = 0.0001	F (1, 10) = 13.85<br>P = 0.0040	F (1, 10) = 325.5<br>P = <0.0001
Genotype x Model	F (1, 10) = 42.51<br>P = <0.0001	F (1, 10) = 15.26<br>P = 0.0029	F (1, 10) = 244.9<br>P = <0.0001

Ab = antibody.

Supplementary Table 6. One-way ANOVA for Figure 4 C.

Capture Ab	Detection Ab	HTRF
2B7	MW1	F (2, 9) = 9.989, P = 0.0052
2B7	M81	F (2, 9) = 169.3, P = <0.0001

Ab = antibody.

Supplementary Table 7. One-way ANOVA for Figure 5.

HTRF			
Donor Ab	Acceptor Ab	Cortex	Striatum
2B7	MW8	F (2, 9) = 20.69, P = 0.0004	F (2, 9) = 64.92, P = <0.0001
MW1	MW8	F (2, 9) = 4.302, P = 0.0489	F (2, 9) = 8.562, P = 0.0083
AlphaLISA			
Donor Ab	Acceptor Ab	Cortex	Striatum
MW8	2B7	F (2, 8) = 430.8, P = <0.0001	F (2, 8) = 146.5, P = <0.0001
MW1	MW8	F (2, 9) = 80.02, P = <0.0001	F (2, 7) = 72.34, P = <0.0001
MSD			
Capture Ab	Detection Ab	Cortex	Striatum
2B7	MW8	F (2, 9) = 57.04, P = <0.0001	F (2, 9) = 51.67, P = <0.0001
MW1	MW8	F (2, 9) = 20.44, P = 0.0005	F (2, 9) = 12.48, P = 0.0025

Ab = antibody.

Supplementary Table 8. One-way ANOVA for Figure 6.

HTRF			
Donor Ab	Acceptor Ab	Cortex	Striatum
MAB5490	MAB2166		
Genotype		F (1, 18) = 3.658, P = 0.0718	F (1, 18) = 1.908, P = 0.1841
Age		F (2, 18) = 2.664, P = 0.0970	F (2, 18) = 0.7286, P = 0.4963
Age x Genotype		F (2, 18) = 0.005961, P = 0.9941	F (2, 18) = 0.4022, P = 0.6747
Donor Ab	Acceptor Ab	Cortex	Striatum
D7F7	MAB5490		
Genotype		F (1, 18) = 18.21, P = 0.0005	F (1, 18) = 39.49, P =<0.0001
Age		F (2, 18) = 9.899, P = 0.0013	F (2, 18) = 28.62, P = <0.0001
Age x Genotype		F (2, 18) = 0.4722, P = 0.6311	F (2, 18) = 0.3659, P = 0.6986
AlphaLISA			
Capture Ab	Detection Ab	Cortex	Striatum
MAB5490	MAB2166		
Genotype		F (1, 16) = 8.705, P = 0.0094	F (1, 18) = 15.27, P=0.0010
Age		F (2, 16) = 0.5328, P = 0.5970	F (2, 18) = 2.456, P=0.1140
Age x Genotype		F (2, 16) = 1.454, P = 0.2628	F (2, 18) = 5.207, P = 0.0164
MSD			
Donor Ab	Acceptor Ab	Cortex	Striatum
D7F7	MAB5490		
Genotype		F (1, 18) = 30.36, P = <0.0001	F (1, 18) = 180.6, P = <0.0001
Age		F (2, 18) = 15.25, P = 0.0001	F (2, 18) = 1.611, P = 0.2273
Age x Genotype		F (2, 18) = 6.057, P = 0.0097	F (2, 18) = 8.559, P = 0.0024
Capture Ab	Detection Ab	Cortex	Striatum
MAB5490	MAB2166		
Genotype		F (1, 18) = 288.5, P=<0.001	F (1, 18) = 80.43, P=<0.001
Age		F (2, 18) = 98.27, P=<0.001	F (2, 18) = 10.10, P=0.0011
Age x Genotype		F (2, 18) = 1.137, P=0.3429	F (2, 18) = 0.6380, P=0.5399

Ab = antibody.

Supplementary Table 9. One-way ANOVA for Figure 7.

HTRF			
Donor Ab	Acceptor Ab	Cortex	Striatum
4C9	MW8	F (2, 9) = 211.0, P = <0.0001	F (2, 9) = 121.9, P = <0.0001
MW8	2B7	F (2, 9) = 47.11, P = <0.0001	F (2, 9) = 17.46, P = 0.0008
AlphaLISA			
Donor Ab	Acceptor Ab	Cortex	Striatum
4C9	MW8	F (2, 9) = 1029, P = <0.0001	F (2, 9) = 240.7, P = <0.0001
MAB2166	MW8	F (2, 9) = 61.66, P = <0.0001	F (2, 9) = 78.21, P = <0.0001
MSD			
Capture Ab	Detection Ab	Cortex	Striatum
MW8	4C9	F (2, 9) = 69.56, P = <0.0001	F (2, 9) = 75.91, P = <0.0001
MW8	2B7	F (2, 9) = 30.52, P = <0.0001	F (2, 9) = 61.45, P = <0.0001
MW8	MAB2166	F (2, 9) = 54.36, P = <0.0001	F (2, 9) = 146.7, P = <0.0001
MW8	MAB5490	F (2, 9) = 60.22, P = <0.0001	F (2, 9) = 14.87, P = 0.0014

Ab = antibody.

Supplementary Table 10. Two-way ANOVA for Figure 8.

HTRF			
Donor Ab	Acceptor Ab	Cortex	Striatum
MAB5490	MAB2166		
Genotype		F (1, 18) = 3.658, P = 0.0718	F (1, 18) = 1.908, P = 0.1841
Age		F (2, 18) = 2.664, P = 0.0970	F (2, 18) = 0.7286, P = 0.4963
Age x Genotype		F (2, 18) = 0.005961, P = 0.9941	F (2, 18) = 0.4022, P = 0.6747
AlphaLISA			
Donor Ab	Acceptor Ab	Cortex	Striatum
MAB5490	MAB2166		
Genotype		F (1, 16) = 8.705, P = 0.0094	F (1, 18) = 15.27, P=0.0010
Age		F (2, 16) = 0.5328, P = 0.5970	F (2, 18) = 2.456, P=0.1140
Age x Genotype		F (2, 16) = 1.454, P = 0.2628	F (2, 18) = 5.207, P = 0.0164
MSD			
Capture Ab	Detection Ab	Cortex	Striatum
D7F7	MAB5490		
Genotype		F (1, 18) = 30.36, P = <0.0001	F (1, 18) = 180.6, P = <0.0001
Age		F (2, 18) = 15.25, P = 0.0001	F (2, 18) = 1.611, P = 0.2273
Age x Genotype		F (2, 18) = 6.057, P = 0.0097	F (2, 18) = 8.559, P = 0.0024

Ab = antibody.

Supplementary Table 11. One-way ANOVA for Supplementary Figure 1.

Donor/ Capture Antibody	Acceptor/ Detection Antibody	HTRF	AlphaLISA	MSD
2B7	MW1	F (2, 9) = 695.8<br>P = <0.0001	F (2, 6) = 50.53<br>P = 0.0002	F (2, 6) = 3.435<br>P = 0.1013
	4C9	F (2, 9) = 184.3<br>P = <0.0001	F (2, 6) = 106.6<br>P = <0.0001	F (2, 6) = 758.9<br>P = <0.0001
	MW8	F (2, 9) = 33.71<br>P = <0.0001	F (2, 6) = 9.381<br>P = 0.0142	F (2, 6) = 716.4<br>P = <0.0001
MW1	2B7	F (2, 9) = 266.6<br>P = <0.0001	F (2, 6) = 46.02<br>P = 0.0002	F (2, 6) = 231.0<br>P = <0.0001
	4C9	F (2, 9) = 0.2550<br>P = 0.7803	F (2, 6) = 92.40<br>P = <0.0001	F (2, 6) = 29.72<br>P = 0.0008
	MW8	F (2, 9) = 0.00044<br>P = 0.9996	F (2, 9) = 23.56<br>P = 0.0003	F (2, 6) = 41.86<br>P = 0.0003
	MAB5490	F (2, 9) = 27.49<br>P = 0.0001	F (2, 9) = 3.758<br>P = 0.0651	F (2, 6) = 13.19<br>P = 0.0064
	MAB2166	F (2, 9) = 37.01<br>P = <0.0001	F (2, 9) = 2.235<br>P = 0.1629	F (2, 6) = 4.908<br>P = 0.0546
4C9	2B7	F (2, 9) = 427.0<br>P = <0.0001	F (2, 6) = 750.4<br>P = <0.0001	F (2, 6) = 8.726<br>P = 0.0167
	MW1	F (2, 9) = 165.6<br>P = <0.0001	F (2, 6) = 0.3706<br>P = 0.7051	F (2, 6) = 2.802<br>P = 0.1382
	MW8	F (2, 9) = 533.2,<br>P = <0.0001	F (2, 6) = 66.40<br>P = <0.0001	F (2, 6) = 176.7<br>P = <0.0001
	MAB5490	F (2, 9) = 136.7,<br>P = <0.0001	F (2, 7) = 3.687<br>P = 0.0806	F (2, 6) = 24.60<br>P = 0.0013
	MAB2166	F (2, 9) = 78.45,<br>P = <0.0001	F (2, 9) = 4.816<br>P = 0.0378	F (2, 6) = 3.864<br>P = 0.0835
MW8	2B7	F (2, 9) = 196.7<br>P = <0.0001	F (2, 6) = 293.0<br>P = <0.0001	F (2, 6) = 53.51<br>P = 0.0001
	MW1	F (2, 9) = 1.592<br>P = 0.2558	F (2, 9) = 0.5347<br>P = 0.6034	F (2, 6) = 1.204<br>P = 0.3635
	4C9	F (2, 9) = 109.2<br>P = <0.0001	F (2, 6) = 39.49<br>P = 0.0004	F (2, 6) = 593.6<br>P = <0.0001
	MAB5490	F (2, 9) = 0.1119<br>P = 0.8954	F (2, 7) = 0.6499<br>P = 0.5509	F (2, 6) = 88.08<br>P = <0.0001
	MAB2166	F (2, 9) = 15.41<br>P = 0.0012	F (2, 9) = 0.5735<br>P = 0.5829	F (2, 6) = 181.7<br>P = <0.0001
MAB5490	MW1	F (2, 9) = 17.81<br>P = 0.0007	F (2, 9) = 2.614<br>P = 0.1273	F (2, 6) = 5.600<br>P = 0.0424
	4C9	F (2, 9) = 7.282<br>P = 0.0132	F (2, 8) = 12.88<br>P = 0.0032	F (2, 6) = 14.13<br>P = 0.0054
	MW8	F (2, 9) = 3.687<br>P = 0.0677	F (2, 8) = 10.08<br>P = 0.0065	F (2, 6) = 0.8842<br>P = 0.4607
MAB2166	MW1	F (2, 9) = 22.63<br>P = 0.0003	F (2, 9) = 2.624<br>P = 0.1265	F (2, 6) = 126.3<br>P = <0.0001
	4C9	F (2, 9) = 0.5832<br>P = 0.5779	F (2, 8) = 10.56<br>P = 0.0057	F (2, 6) = 4.188<br>P = 0.0727
	MW8	F (2, 9) = 0.2771<br>P = 0.7642	F (2, 8) = 76.30<br>P = <0.0001	F (2, 5) = 11.02<br>P = 0.0147

Ab = antibody.

Supplementary Table 12. Two-way ANOVA for Supplementary Figure 1.

	HTRF	AlphaLISA	MSD
Donor/ Capture Ab: 2B7 Acceptor/ Detection Ab: MAB5490			
Genotype	F (1, 18) = 2464<br>P = <0.0001	F (1, 18) = 26.04<br>P = <0.0001	F (1, 9) = 151.9<br>P = <0.0001
Age	F (2, 18) = 25.07<br>P = <0.0001	F (2, 18) = 2.334<br>P = 0.1256	F (2, 9) = 3.903<br>P = 0.0602
Genotype x Age	F (2, 18) = 9.909<br>P = 0.0013	F (2, 18) = 0.5612<br>P = 0.5802	F (2, 9) = 1.527<br>P = 0.2686
Donor/ Capture Ab: MAB5490 Acceptor/ Detection Ab: 2B7			
Genotype	F (1, 18) = 274.3<br>P = <0.0001	F (1, 18) = 56.14<br>P = <0.0001	F (1, 9) = 40.95<br>P = 0.0001
Age	F (2, 18) = 1.993<br>P = 0.1653	F (2, 18) = 3.550<br>P = 0.0501	F (2, 9) = 4.916<br>P = 0.0361
Genotype x Age	F (2, 18) = 1.201<br>P = 0.3239	F (2, 18) = 1.386<br>P = 0.2755	F (2, 9) = 2.777<br>P = 0.1150
Donor/ Capture Ab: 2B7 Acceptor/ Detection Ab: MAB2166			
Genotype	F (1, 18) = 3287<br>P = <0.0001	F (1, 18) = 1.667<br>P = 0.2129	F (1, 9) = 119.4<br>P = <0.0001
Age	F (2, 18) = 50.38<br>P = <0.0001	F (2, 18) = 1.947<br>P = 0.1716	F (2, 9) = 2.699<br>P = 0.1207
Genotype x Age	F (2, 18) = 32.00<br>P = <0.0001	F (2, 18) = 0.2905<br>P = 0.7513	F (2, 9) = 1.698<br>P = 0.2367
Donor/ Capture Ab: MAB2166 Acceptor/ Detection Ab: 2B7			
Genotype	F (1, 18) = 255.3<br>P = <0.0001	F (1, 17) = 55.70<br>P = <0.0001	F (1, 9) = 56.44<br>P = <0.0001
Age	F (2, 18) = 0.9222<br>P = 0.4156	F (2, 17) = 0.1115<br>P = 0.8951	F (2, 9) = 1.840<br>P = 0.2139
Genotype x Age	F (2, 18) = 1.389<br>P = 0.2748	F (2, 17) = 2.553<br>P = 0.1072	F (2, 9) = 3.607<br>P = 0.0707
Donor/ Capture Ab: MAB5490 Acceptor/ Detection Ab: MAB2166			
Genotype	F (1, 18) = 68.55<br>P = <0.0001	F (1, 18) = 18.14<br>P = 0.0005	F (1, 9) = 93.64<br>P = <0.0001
Age	F (2, 18) = 36.88<br>P = <0.0001	F (2, 18) = 5.753<br>P = 0.0117	F (2, 9) = 7.617<br>P = 0.0116
Genotype x Age	F (2, 18) = 1.465<br>P = 0.2574	F (2, 18) = 0.2872<br>P = 0.7537	F (2, 9) = 0.04398<br>P = 0.9572
Donor/ Capture Ab: MAB2166 Acceptor/ Detection Ab: MAB5490			
Genotype	F (1, 18) = 13.79<br>P = 0.0016	F (1, 18) = 7.410<br>P = 0.0140	F (1, 9) = 3.387<br>P = 0.0989
Age	F (2, 18) = 0.1354<br>P = 0.8743	F (2, 18) = 0.4878<br>P = 0.6219	F (2, 9) = 3.077<br>P = 0.0959
Genotype x Age	F (2, 18) = 3.473<br>P = 0.0530	F (2, 18) = 0.3690<br>P = 0.6965	F (2, 9) = 1.958<br>P = 0.1968

Ab = antibody.

Supplementary Table 13. Two way ANOVA for Supplementary Figure 7.

	HTRF	AlphaLISA	MSD
Donor/ Capture Ab: MAB5490 Acceptor/ Detection Ab: 4C9			
Genotype	F (2, 17) = 140.1<br>P = <0.0001	F (2, 12) = 45.67<br>P = <0.0001	F (2, 12) = 411.6<br>P = <0.0001
Model	F (1, 17) = 115.9<br>P = <0.0001	F (1, 12) = 58.06<br>P = <0.0001	F (1, 12) = 598.9<br>P = <0.0001
Genotype x Model	F (2, 17) = 22.17<br>P = <0.0001	F (2, 12) = 29.85<br>P = <0.0001	F (2, 12) = 255.9<br>P = <0.0001
Donor/ Capture Ab: 4C9 Acceptor/ Detection Ab: MAB5490			
Genotype	F (2, 17) = 161.9<br>P = <0.0001	F (2, 12) = 168.6<br>P = <0.0001	F (2, 12) = 1184<br>P = <0.0001
Model	F (1, 17) = 178.2<br>P = <0.0001	F (1, 12) = 127.3<br>P = <0.0001	F (1, 12) = 1414<br>P = <0.0001
Genotype x Model	F (2, 17) = 61.96<br>P = <0.0001	F (2, 12) = 58.16<br>P = <0.0001	F (2, 12) = 508.1<br>P = <0.0001
Donor/ Capture Ab: 4C9 Acceptor/ Detection Ab: MAB2166			
Genotype	F (2, 17) = 287.3<br>P = <0.0001	F (2, 12) = 214.5<br>P = <0.0001	F (2, 12) = 1448<br>P = <0.0001
Model	F (1, 17) = 226.6<br>P = <0.0001	F (1, 12) = 102.3<br>P = <0.0001	F (1, 12) = 1760<br>P = <0.0001
Genotype x Model	F (2, 17) = 16.92<br>P = <0.0001	F (2, 12) = 31.44<br>P = <0.0001	F (2, 12) = 707.7<br>P = <0.0001
Donor/ Capture Ab: MAB2166 Acceptor/ Detection Ab: 4C9			
Genotype	F (2, 17) = 1285<br>P = <0.0001	F (2, 12) = 111.1<br>P = <0.0001	F (2, 12) = 1386<br>P = <0.0001
Model	F (1, 17) = 1189<br>P = <0.0001	F (1, 12) = 81.03<br>P = <0.0001	F (1, 12) = 2165<br>P = <0.0001
Genotype x Model	F (2, 17) = 415.6<br>P = <0.0001	F (2, 12) = 34.02<br>P = <0.0001	F (2, 12) = 740.5<br>P = <0.0001

Ab = antibody.

Supplementary Table 14. Two-way ANOVA for Supplementary Figure 13.

	HTRF	AlphaLISA	MSD
Donor/ Capture Ab: 2B7 Acceptor/ Detection Ab: MAB5490			
Genotype	F (2, 17) = 394.3<br>P = <0.0001	F (2, 12) = 8.424<br>P = 0.0052	F (2, 12) = 29.38<br>P = <0.0001
Model	F (1, 17) = 1517<br>P = <0.0001	F (1, 12) = 2.607<br>P = 0.1323	F (1, 12) = 505.3<br>P = <0.0001
Genotype x Model	F (2, 17) = 438.3<br>P = <0.0001	F (2, 12) = 6.136<br>P = 0.0146	F (2, 12) = 103.6<br>P = <0.0001
Donor/ Capture Ab: MAB5490 Acceptor/ Detection Ab: 2B7			
Genotype	F (2, 17) = 40.16<br>P = <0.0001	F (2, 12) = 11.66<br>P = 0.0015	F (2, 12) = 12.10<br>P = 0.0013
Model	F (1, 17) = 120.2<br>P = <0.0001	F (1, 12) = 6.262<br>P = 0.0278	F (1, 12) = 61.22<br>P = <0.0001
Genotype x Model	F (2, 17) = 113.4<br>P = <0.0001	F (2, 12) = 9.212<br>P = 0.0038	F (2, 12) = 6.435<br>P = 0.0126
Donor/ Capture Ab: 2B7 Acceptor/ Detection Ab: MAB2166			
Genotype	F (2, 17) = 320.7<br>P = <0.0001	F (2, 12) = 11.81<br>P = 0.0015	F (2, 12) = 16.24<br>P = 0.0004
Model	F (1, 17) = 1352<br>P = <0.0001	F (1, 12) = 10.16<br>P = 0.0078	F (1, 12) = 189.5<br>P = <0.0001
Genotype x Model	F (2, 17) = 286.3<br>P = <0.0001	F (2, 12) = 4.589<br>P = 0.0331	F (2, 12) = 133.1<br>P = <0.0001
Donor/ Capture Ab: MAB2166 Acceptor/ Detection Ab: 2B7			
Genotype	F (2, 17) = 272.1<br>P = <0.0001	F (2, 12) = 20.05<br>P = 0.0001	F (2, 12) = 12.38<br>P = 0.0012
Model	F (1, 17) = 757.5<br>P = <0.0001	F (1, 12) = 38.20<br>P = <0.0001	F (1, 12) = 44.26<br>P = <0.0001
Genotype x Model	F (2, 17) = 283.0<br>P = <0.0001	F (2, 12) = 3.874<br>P = 0.0503	F (2, 12) = 52.36<br>P = <0.0001
Donor/ Capture Ab: MAB5490 Acceptor/ Detection Ab: MAB2166			
Genotype	F (2, 17) = 2.519<br>P = 0.1101	F (2, 17) = 3.121<br>P=0.0701	F (2, 12) = 9.873<br>P = 0.0029
Model	F (1, 17) = 46.07<br>P = <0.0001	F (1, 17) = 0.01076<br>P=0.9186	F (1, 12) = 167.9<br>P = <0.0001
Genotype x Model	F (2, 17) = 40.90<br>P = <0.0001	F (2, 17) = 0.2497<br>P=0.7819	F (2, 12) = 53.32<br>P = <0.0001
Donor/ Capture Ab: MAB2166 Acceptor/ Detection Ab: MAB5490			
Genotype	F (2, 17) = 12.67<br>P = 0.0004	F (2, 18) = 2.855<br>P=0.0838	F (2, 12) = 4.574<br>P = 0.0334
Model	F (1, 17) = 144.7<br>P = <0.0001	F (1, 18) = 9.087<br>P= 0.0074	F (1, 12) = 60.61<br>P = <0.0001
Genotype x Model	F (2, 17) = 41.05<br>P = <0.0001	F (2, 18) = 13.00<br>P=0.0003	F (2, 12) = 17.43<br>P = 0.0003

Ab = antibody.

Supplementary Table 15. Two-way ANOVA for Supplementary Figure 14.

	HTRF	MSD
Donor/ Capture Ab: 2B7 Acceptor/ Detection Ab: D7F7		
Genotype	F (2, 18) = 112.4, P = <0.0001	F (2, 12) = 26.53, P = <0.0001
Model	F (1, 18) = 394.0, P = <0.0001	F (1, 12) = 331.1, P = <0.0001
Genotype x Model	F (2, 18) = 155.5, P = <0.0001	F (2, 12) = 48.35, P = <0.0001
Donor/ Capture Ab: D7F7 Acceptor/ Detection Ab: 2B7		
Genotype	F (2, 18) = 83.45, P = <0.0001	F (2, 12) = 22.37, P = <0.0001
Model	F (1, 18) = 133.8, P = <0.0001	F (1, 12) = 198.1, P = <0.0001
Genotype x Model	F (2, 18) = 87.15, P = <0.0001	F (2, 12) = 53.95, P = <0.0001
Donor/ Capture Ab: MAB5490 Acceptor/ Detection Ab: D7F7		
Genotype	F (2, 18) = 4.913, P = 0.0198	F (2, 12) = 12.61, P = 0.0011
Model	F (1, 18) = 49.59, P = <0.0001	F (1, 12) = 174.5, P = <0.0001
Genotype x Model	F (2, 18) = 31.94, P = <0.0001	F (2, 12) = 27.66, P = <0.0001
Donor/ Capture Ab: D7F7 Acceptor/ Detection Ab: MAB5490		
Genotype	F (2, 18) = 3.126, P = 0.0684	F (2, 12) = 4.238, P = 0.0405
Model	F (1, 18) = 10.23, P = 0.0050	F (1, 12) = 106.4, P = <0.0001
Genotype x Model	F (2, 18) = 14.01, P = 0.0002	F (2, 12) = 13.63, P = 0.0008
Donor/ Capture Ab: MAB2166 Acceptor/ Detection Ab: D7F7		
Genotype	F (2, 18) = 5.460, P = 0.0140	F (2, 12) = 26.19, P = <0.0001
Model	F (1, 18) = 17.52, P = 0.0006	F (1, 12) = 183.0, P = <0.0001
Genotype x Model	F (2, 18) = 14.65, P = 0.0002	F (2, 12) = 51.57, P = <0.0001
Donor/ Capture Ab: D7F7 Acceptor/ Detection Ab: MAB2166		
Genotype	F (2, 18) = 0.02876, P = 0.9717	F (2, 12) = 40.01, P = <0.0001
Model	F (1, 18) = 7.501, P = 0.0135	F (1, 12) = 179.5, P = <0.0001
Genotype x Model	F (2, 18) = 0.6745, P = 0.5218	F (2, 12) = 23.68, P = <0.0001

Ab = antibody.
